# A distinct isoform of lymphoid enhancer binding factor 1 (LEF1) epigenetically restricts EBV reactivation to maintain viral latency

**DOI:** 10.1371/journal.ppat.1011873

**Published:** 2023-12-19

**Authors:** B. J. H. Ward, Kanchanjunga Prasai, Danielle L. Schaal, Jian Wang, Rona S. Scott

**Affiliations:** 1 Department of Microbiology and Immunology, Louisiana State University Health Sciences Center-Shreveport, Shreveport, Louisiana, United States of America; 2 Feist-Weiller Cancer Center, Louisiana State University Health Sciences Center-Shreveport, Shreveport, Louisiana, United States of America; 3 Center for Applied Immunology and Pathological Processes, Louisiana State University Health Sciences Center-Shreveport, Shreveport, Louisiana, United States of America; Wistar Institute, UNITED STATES

## Abstract

As a human tumor virus, EBV is present as a latent infection in its associated malignancies where genetic and epigenetic changes have been shown to impede cellular differentiation and viral reactivation. We reported previously that levels of the Wnt signaling effector, lymphoid enhancer binding factor 1 (LEF1) increased following EBV epithelial infection and an epigenetic reprogramming event was maintained even after loss of the viral genome. Elevated LEF1 levels are also observed in nasopharyngeal carcinoma and Burkitt lymphoma. To determine the role played by LEF1 in the EBV life cycle, we used in silico analysis of EBV type 1 and 2 genomes to identify over 20 Wnt-response elements, which suggests that LEF1 may bind directly to the EBV genome and regulate the viral life cycle. Using CUT&RUN-seq, LEF1 was shown to bind the latent EBV genome at various sites encoding viral lytic products that included the immediate early transactivator BZLF1 and viral primase BSLF1 genes. The LEF1 gene encodes various long and short protein isoforms. siRNA depletion of specific LEF1 isoforms revealed that the alternative-promoter derived isoform with an N-terminal truncation (ΔN LEF1) transcriptionally repressed lytic genes associated with LEF1 binding. In addition, forced expression of the ΔN LEF1 isoform antagonized EBV reactivation. As LEF1 repression requires histone deacetylase activity through either recruitment of or direct intrinsic histone deacetylase activity, siRNA depletion of LEF1 resulted in increased histone 3 lysine 9 and lysine 27 acetylation at LEF1 binding sites and across the EBV genome. Taken together, these results indicate a novel role for LEF1 in maintaining EBV latency and restriction viral reactivation via repressive chromatin remodeling of critical lytic cycle factors.

## Author summary

EBV-positive cancers are directly linked to viral latency established by epigenetic silencing of the viral genome. EBV usurps the host epigenetic machinery to silence its own genome for regulation of its viral life cycle. In turn, such epigenetic manipulation by EBV also affects host gene expression profiles resulting in oncogenic phenotypes and cellular states supportive of latent infection. Understanding how the EBV life cycle is regulated epigenetically by host transcription factors during latent infection may help identify new areas of therapeutic focus in treating EBV-associated cancers. LEF1 is a Wnt-signaling factor possessing intrinsic histone deacetylase activity involved in the determination of cell fate, stemness, differentiation, and motility. In this study, we determined that LEF1 binds the EBV genome during latency, regulating viral gene expression and chromatin acetylation. Depleting specific isoforms of LEF1 revealed the N-terminal truncated ΔN LEF1 isoform mediates repression of viral gene expression via deacetylation of the latent genome. Our data indicate for the first time that the Wnt-signaling effector LEF1 is involved in the maintenance of EBV latency via suppression of viral lytic gene expression.

## Introduction

Epstein-Barr virus (EBV) is a lymphotropic and epitheliotropic gammaherpesvirus that establishes a lifelong infection in its human host [1–3]. In common with all *Herpesviridae* family members, EBV displays a biphasic life cycle characterized by lytic production of progeny virions and distinct periods of transcriptionally repressed latency [4,5]. EBV latency and reactivation is highly regulated by host transcriptional factors and cellular differentiation in B cells and epithelial cells. EBV lytic replication initiates following infection of differentiated oral epithelial cells or in latently infected B lymphocytes undergoing plasma cell terminal differentiation [6,7]. Additionally, EBV reactivation may occur as a response to cellular stress including danger signals and hypoxic conditions [8,9]. Lytic gene expression occurs as a temporal cascade of viral gene subsets resulting in production of progeny virions. EBV immediate early (IE) genes *BZLF1* and *BRLF1* are transactivated by host differentiation-induced transcription factors such as PRDM1 and KLF4 [10–12]. Viral IE genes then facilitate expression of the early gene (E) products [13]. Early genes encode the viral replication machinery required for rolling-circle amplification of the viral genome [14,15]. BMRF1 gene product EA-D encodes the viral processivity factor. Viral genome replication then facilitates late gene (L) expression of viral structural proteins and assembly of new virions [16]

EBV-associated malignancies such as Burkitt lymphoma (BL), Hodgkin’s lymphoma (HL), Diffuse large B-cell lymphoma (DLBCL), post-transplant lymphoproliferative disease (PTLD), nasopharyngeal carcinoma (NPC) and gastric carcinoma (GC) are linked to viral latency [17–22]. Following initial infection of the oral epithelium viral double-stranded DNA enters the host nucleus in an epigenetically naïve state [6,7]. EBV uses the host epigenetic machinery to establish latency and regulate life cycle phases via reversible silencing of lytic gene expression [23,24]. The latent EBV genome becomes highly methylated and chromatinized with repressive histone modifications including H3K9me3 and H3K27me3 restricting DNA access to the transcriptional machinery [24,25]. However, the immediate early gene promoter of BZLF1 (Zp) typically exhibits a low level of CpG methylation [26,27]. Rather, the BZLF1 promoter is regulated by repressive histone marks during viral latency which can be rapidly erased and rewritten with histone acetylation to induce BZLF1 expression [23,28,29]. The observation that BZLF1 silencing can be reversed with HDAC inhibitor treatments such as trichostatin A and sodium butyrate illustrate the important role of histone acetylation in regulation of the viral life cycle [30–32]. BZLF1 expression, and therefore EBV latency, is also regulated by the balance between host repressive and activating transcription factors [33–37]. BZLF1 transactivator (Z) is a pioneer transcription factor that can preferentially binds methylated DNA on lytic gene promoters to induce EBV lytic replication [38]. Thus, host epigenetic mechanisms are therefore crucial for establishing viral latency and facilitating viral reactivation. Viral exploitation of host epigenetic factors is a recognized mechanism contributing to the EBV oncogenic potential [39]. EBV perturbation of the host epigenome may not only enhance survival and proliferation but also promote an undifferentiated cellular phenotype supportive of viral latency.

We previously demonstrated that telomerase-immortalized normal oral keratinocytes (NOK) latently infected with EBV increased expression of the host Wnt-signaling transcription factor, lymphoid enhancer binding factor 1 (LEF1) [40]. The EBV-infected NOK showed resistance to differentiation and a LEF1-dependent increase in cellular invasiveness suggesting that EBV latent infection reprogrammed cells with a basal cell, wound-healing cellular phenotype [40,41]. Importantly, increased LEF1 is also observed in latent-EBV-associated cancers including Burkitt lymphoma, nasopharyngeal carcinoma, and gastric carcinoma [42–44]. LEF1 is an important survival and proliferation factor regulating cellular stemness, renewal, lineage determination, epithelial to mesenchymal transitioning (EMT), and motility [45,46]. LEF1 is typically expressed in precursor and progenitor cells early in development. In lymphocytes, LEF1 is expressed in pre-B cells and mature T cells, but not mature B lymphocytes [47–49]. In the epidermis, LEF1 is expressed in embryonic and neonatal keratinocytes and fibroblasts. LEF1 expression is not typically detected in adult skin except for expression in bulge stem cells in hair follicles [50,51]. LEF1 acts as either a transcriptional activator or repressor depending on the context of Wnt-Beta-catenin signaling [52,53]. Transcriptional repression by LEF1 is associated with several co-repressors including members of the Groucho/Transducin-like Enhancer of Split (Gro/TLE) family and recruitment of repressive chromatin remodelers such as histone deacetylases (HDAC) [54]. LEF1 also possesses an intrinsic HDAC activity to directly modify histone acetylation patterns [55]. Transcriptional activation is associated with beta-catenin binding to LEF1 facilitating the displacement of co-repressors proteins in response to Wnt signaling. The architecture of the LEF1 gene gives rise to multiple isoforms through alternative promoter derived transcription and alternative splicing of messenger RNA (mRNA) [56,57]. An amino-terminal truncated LEF1 (ΔN LEF1) expressed from an alternative promoter lacks the beta-catenin binding domain rendering ΔN LEF1 a potent repressive isoform [56–58]. All LEF1 isoforms share a C-terminal DNA binding domain with the possibility of competition among LEF1 isoforms for genomic binding sites. Accordingly, full-length and truncated LEF1 have been shown to display functional antagonism in the transcriptional regulation of target genes [56,58].

LEF1 transcriptional activation of the Notch signaling pathway has been shown to suppress viral reactivation in the closely related gammaherpesvirus Kaposi Sarcoma virus [59]. However, the role of LEF1 in the life cycle of EBV has not been examined. Using an 8 bp consensus recognition site for LEF1, we identified multiple potential binding sites on the EBV Akata genome. Thus, we hypothesized that LEF1 associates directly with the viral genome in latently infected cells to restrict lytic gene expression. Using CUT&RUN-seq combined with siRNA-mediated LEF1 knockdown, we demonstrate that LEF1 binds to the latent EBV genome and that the ΔN LEF1 isoform transcriptionally represses EBV reactivation in latently infected epithelial cells. Additionally, we provide evidence that LEF1 promotes the maintenance of viral latency via deacetylation of the EBV genome. This study identifies a direct role for LEF1 in the regulation of the EBV biphasic life cycle and provides critical insight into how EBV interacts with factors of the Wnt-signaling pathway in latently infected cells.

## Results

### LEF1 binds the EBV genome in latently infected Burkitt lymphoma and epithelial cell lines

**Fig 1:**
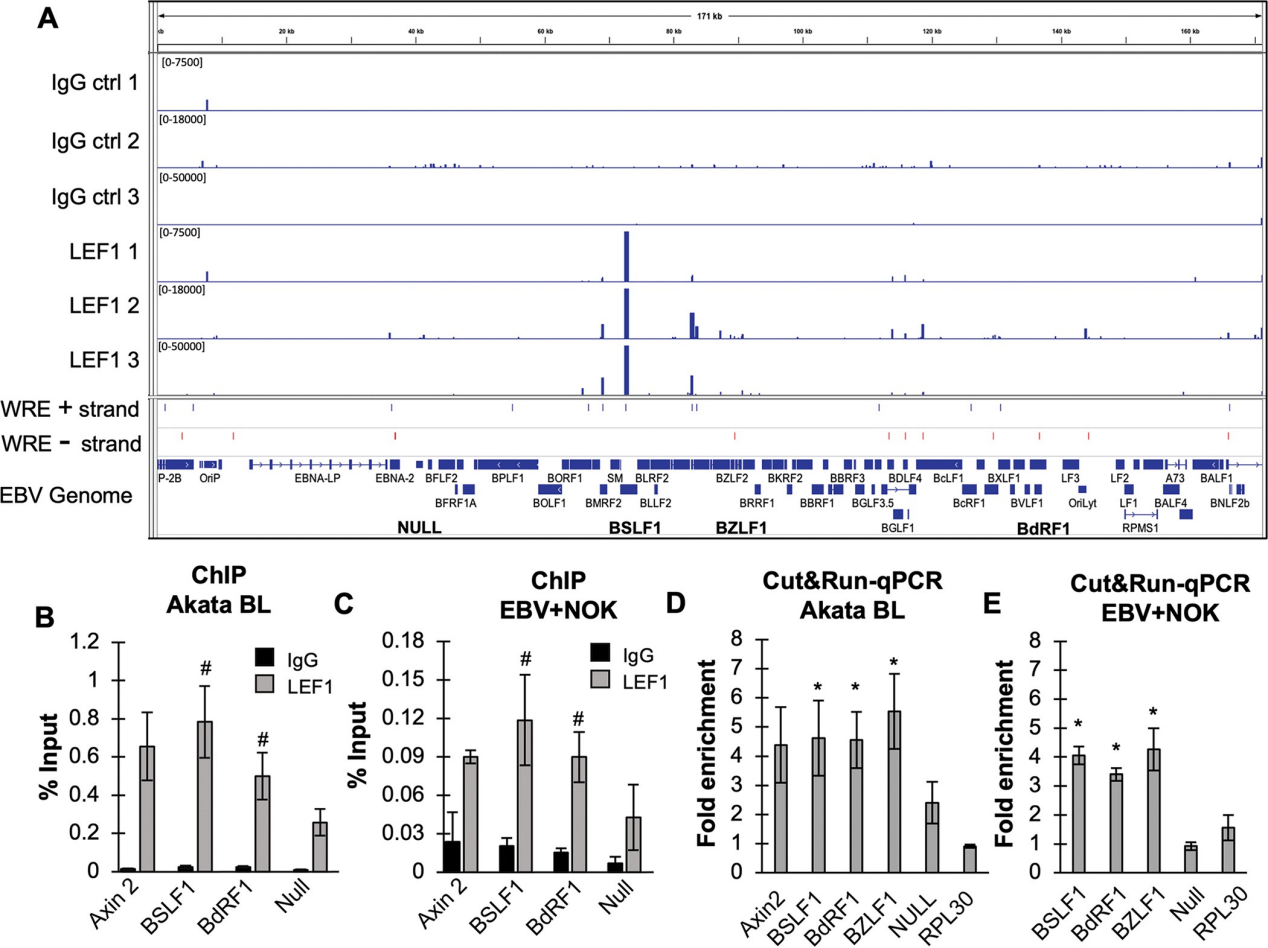
LEF1 binds the EBV genome in latently infected BL and epithelial cells. **(A)** CUT&RUN-seq analysis of LEF1-EBV genome occupancy in latently infected Akata BX1 BL. Shown are the peak maps of IgG controls (tracks 1–3) and LEF1 peaks (tracks 4–6) from triplicate experiments. The predicted WRE (CTTTGWWS) motif is shown on the plus DNA strand (track 7, blue) and minus DNA strand (track 8, red). The gene annotation for the EBV Akata genome (KC 207813) is shown on the bottom track. EBV regions analyzed are indicated in bold letters. **(B)** ChIP-qPCR validation of LEF1 binding sites in Akata BX1 BL and **(C)** EBV+NOK. Primers used are specific for human Axin2 (positive control), EBV lytic genes BSLF1 and BdRF1, and a viral DNA region lacking predicted LEF1 binding sites, LEF1 Null. Shown are the mean and standard deviation of the mean from two independent experiments. Black bars represent IgG percent input; grey bars represent LEF1 percent input^#^, P< 0.1 for Akata BX-1 ChIP and P< 0.06 for EBV+NOK. CUT&RUN-qPCR validation of LEF1 binding sites in **(D)** Akata BX1 BL and **(E)** EBV+NOK. Primers used are specific for human Axin2 (positive control), EBV lytic gene BSLF1, BdRF1, and BZLF1. LEF1 Null on viral DNA (n = 4 Akata BX1; n = 3 EBV+ NOK) and human RPL30 promoter (n = 2 Akata BX1; n = 3 EBV+NOK) were used as negative controls. Fold enrichment (LEF1relative to IgG) is presented as mean and error bars represent the standard error of the mean calculated from four independent experiments for Akata BX1 and three independent experiments for EBV+NOK. Significance testing was assessed by Student’s t-test compared LEF1 enrichment relative to the Null region *, P < 0.05.

Elevated levels of LEF1 have been observed in various EBV-positive cancers such as Burkitt lymphoma and nasopharyngeal carcinoma, as well as following EBV latent infection of the human telomerase-immortalized normal oral keratinocyte cell line (NOK) [40,48]. LEF1 is a member of the TCF/LEF1 family of transcription factors that regulate gene expression in response to Wnt signaling [60,61]. The LEF1/TCF family bind DNA through a conserved high mobility group domain (HMG box) at DNA elements known as Wnt response elements (WRE) [62–64]. Analysis of the EBV Akata type 1 genome (KC_207813) for the 8-bp WRE consensus motif (5’-CTTTGWWS-3’) revealed 25 potential LEF1/TCF binding sites across the EBV genome (Fig 1A). Further analysis of EBV type 1 and type 2 reference genomes showed conservation of 22 WRE motifs in regions encoding both lytic and latent gene products (S1 Fig). We also identified an extended WRE motif (5’WTYYCTTTGATSTT3’) present at BSLF1 in each of the EBV genomes analyzed [64]. To determine whether LEF1 engaged the latent EBV genome, we performed chromatin immunoprecipitation-coupled quantitative PCR (ChIP-qPCR) in EBV+NOK and Akata BX1 BL cell lines. LEF1 occupancy was examined at BSLF1 (viral primase) and BdRF1 (viral capsid scaffold) using primers flanking predicted WRE motifs at these sites (S1 Table). A region lacking a WRE element on the EBV genome (Null) was assessed as a negative control, while LEF1 binding to the AXIN2 promoter served as a positive control [65]. In Akata BX1 BL and EBV+NOK cell lines, LEF1 enrichment was observed at both BSLF1 and BdRF1 regions encoding WRE elements comparable to the LEF1 enrichment detected at the AXIN2 promoter (Fig 1B and 1C), while EBV DNA region lacking a WRE motif showed reduced LEF1 enrichment. To examine LEF1 binding across the Akata EBV genome, a Cleavage Under Targets and Release Using Nuclease assay (CUT&RUN-seq) was performed on native chromatin without fixation [66]. DNA regions bound by LEF1 were labeled using a LEF1 antibody and targeted with a Protein A-G micrococcal nuclease fusion (pAG-MNase) to cleave and release DNA fragments bound to LEF1. Following next generation sequencing, EBV DNA fragments between 50 and 300 bp were analyzed using the Skene CUT&RUN pipeline and peak calling algorithm [66]. As expected from the ChIP results, robust enrichment of LEF1 was observed at BSLF1; however, no peak was detected at BdRF1 (Fig 1A). Several other LEF1 binding peaks were repeatedly detected in replicate experiments with approximately 7 peaks aligning to predicted WRE motifs (S1 Table). LEF1 binding was also detected in regions that had degenerate WRE motifs (S1 Table). For example, LEF1 binding to the BZFL1 promoter mapped to a degenerate WRE (5’-CTTT**A**AAG-3’). To further validate LEF1 binding at BZFL1 and BdRF1, quantitative PCR was used to quantify the enrichment of LEF1 binding at BZLF1 and BdRF1 WRE regions in CUT&RUN assays. Statistically significant increases in LEF1 enrichment were observed at BSLF1, BdRF1, and BZLF1 relative to the LEF1 null region in both Akata BX1 BL and EBV+NOK (Fig 1D and 1E). Together, these results indicated that LEF1 bound various sites across the Akata EBV genome in two latently infected cell lines.

### Various LEF1 isoforms are expressed in EBV latently infected cell lines

**Fig 2:**
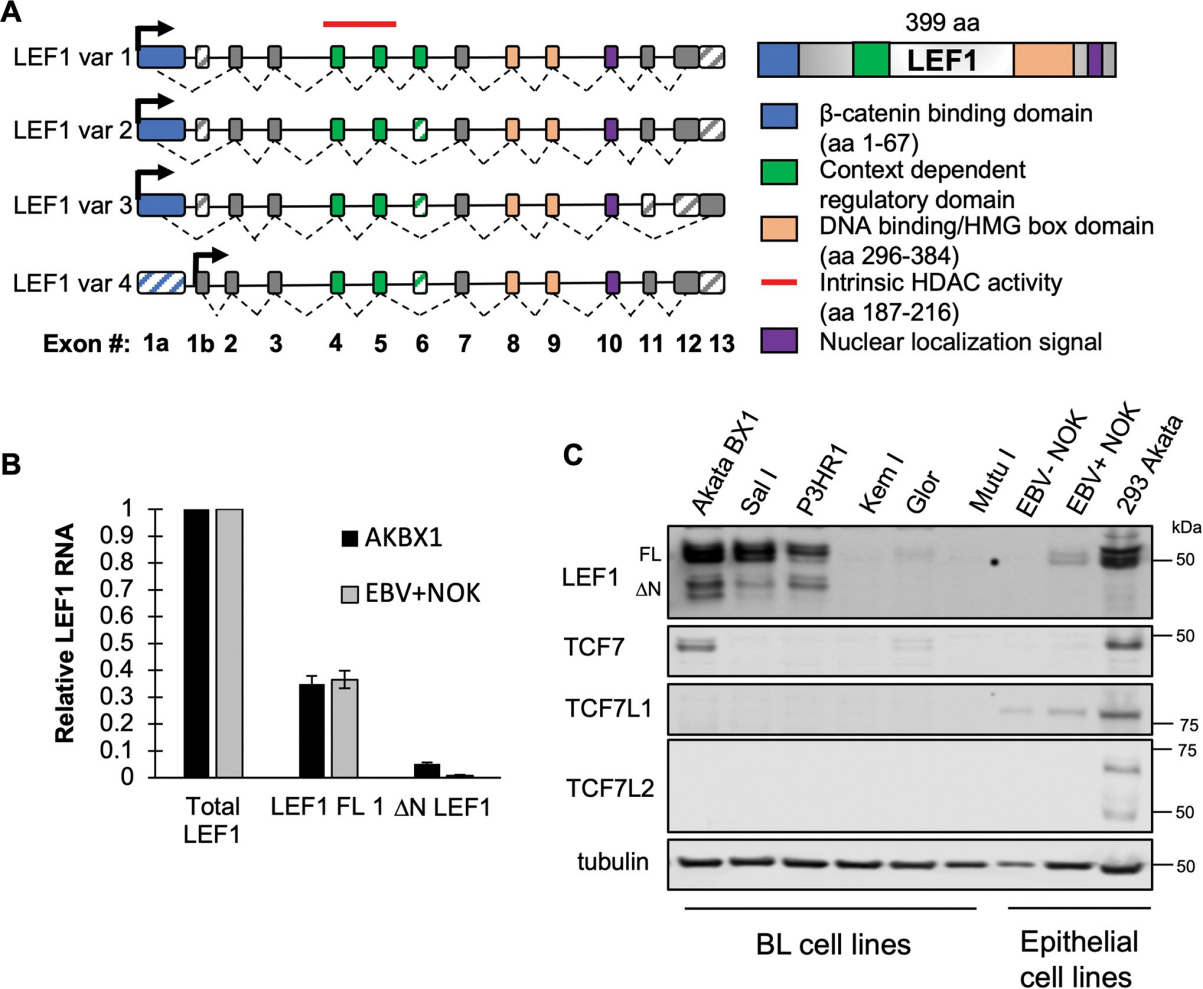
LEF1 full-length and short isoforms are expressed in latently infected EBV-positive cell lines. **(A)** Schematic of LEF1 genomic architecture depicting the exons highlighted in correlation to coded functional domains: beta-catenin binding domain (blue); context dependent regulatory domain (green); HMG BOX DNA binding domain (tan); Nuclear localization signal (purple); intrinsic HDAC domain (red line). Black arrows indicate promoter/transcriptional start sites. Exon splicing of 4 LEF1 transcriptional variants is depicted by hashed lines and exon inclusion is indicated by solid filled exons, while exons excluded from the transcript variant is indicated in a striped pattern. **(B)** RT-qPCR analysis of LEF1 variant 1 and variant 4 (ΔN LEF1) transcript levels relative to total LEF1 transcripts in latently infected Akata BX1 (black bars, n = 4) and EBV+NOKs (gray bars, n = 3). Shown are the mean and standard error of the mean. **(C)** Comparison of LEF1/TCF family protein levels using equivalent cell number (500,000 cells) in a panel of latently infected BL and epithelial cell lines. Immunoblots were probed for LEF1, TCF7/TCF1, TCF7L1/TCF3, TCF7L2/TCF4. Alpha tubulin was used as loading control.

The LEF1/TCF family of transcription factors encode five conserved domains: an amino-terminal beta-catenin binding domain, a context dependent regulatory domain, the high-mobility group domain (HMG)/DNA binding domain, a ‘basic tail’ nuclear localization signal (NLS), and a C-clamp in the carboxy terminus (Fig 2A). The genomic structure of four major variants is shown (Fig 2A) [56,67,68]. All LEF1 isoforms retain the HMG box/ DNA binding domain and capacity to bind DNA. Using reverse transcription quantitative PCR (RT-qPCR), expression of the full-length LEF1 variant 1 and the ΔN truncated LEF1 variant 4 RNA (Fig 2B) was detected in both Akata BX1 BL and EBV+NOK cell lines (Fig 2B). LEF1 protein was detected in Akata BX1 BL and EBV+NOK that included full-length LEF1 isoforms 1–3 at approximately 50 kDa and the N-terminal truncated (ΔN) / isoform 4 of LEF1 at approximately 37 kDa (Fig 2C). EBV+NOK exhibited a 100-fold increase in LEF1 protein vs. uninfected control (Fig 2C) [40]. Although the expression pattern of LEF1 isoforms was similar between the EBV+NOK and Akata BX1 BL cell lines, total LEF1 protein in EBV+NOK was approximately 20% of that observed in Akata BX1 BL (Fig 2C). We extended our analysis to include several EBV+ BL and 293 EBV cell lines to discover that LEF1 was not expressed uniformly in all EBV+BL. AkataBX1 and Salina BL had high levels of LEF1 protein followed by P3HR1 cl16 and Glor BL. Mutu-I and Kem-I had very low to undetectable levels of LEF1 protein. RNA levels of total LEF1 mRNA mirrored the protein levels in each of the BL cell lines (S4 Fig). We also examined TCF transcription factors protein levels in panel of EBV+ cell lines. TCF proteins levels were also variably detected across EBV+ BL cell lines, while EBV-positive epithelial cells were more similar in their TCF/LEF1 expression patterns. TCF7/TCF1 was detected in Akata BX1 BL, Glor, EBV+NOK, and 293 Akata. TCF7L1/TCF3 was detected in parental NOK, EBV+NOK, and 293 Akata (Fig 2C). TCF7L2/TCF4 was only detected in 293 Akata. We focused this study on Akata BX1 BL and EBV+NOK as both cell lines showed LEF1 binding to the EBV genome.

### LEF1 represses EBV lytic gene expression in an isoform-dependent manner in EBV-positive epithelial cells

**Fig 3:**
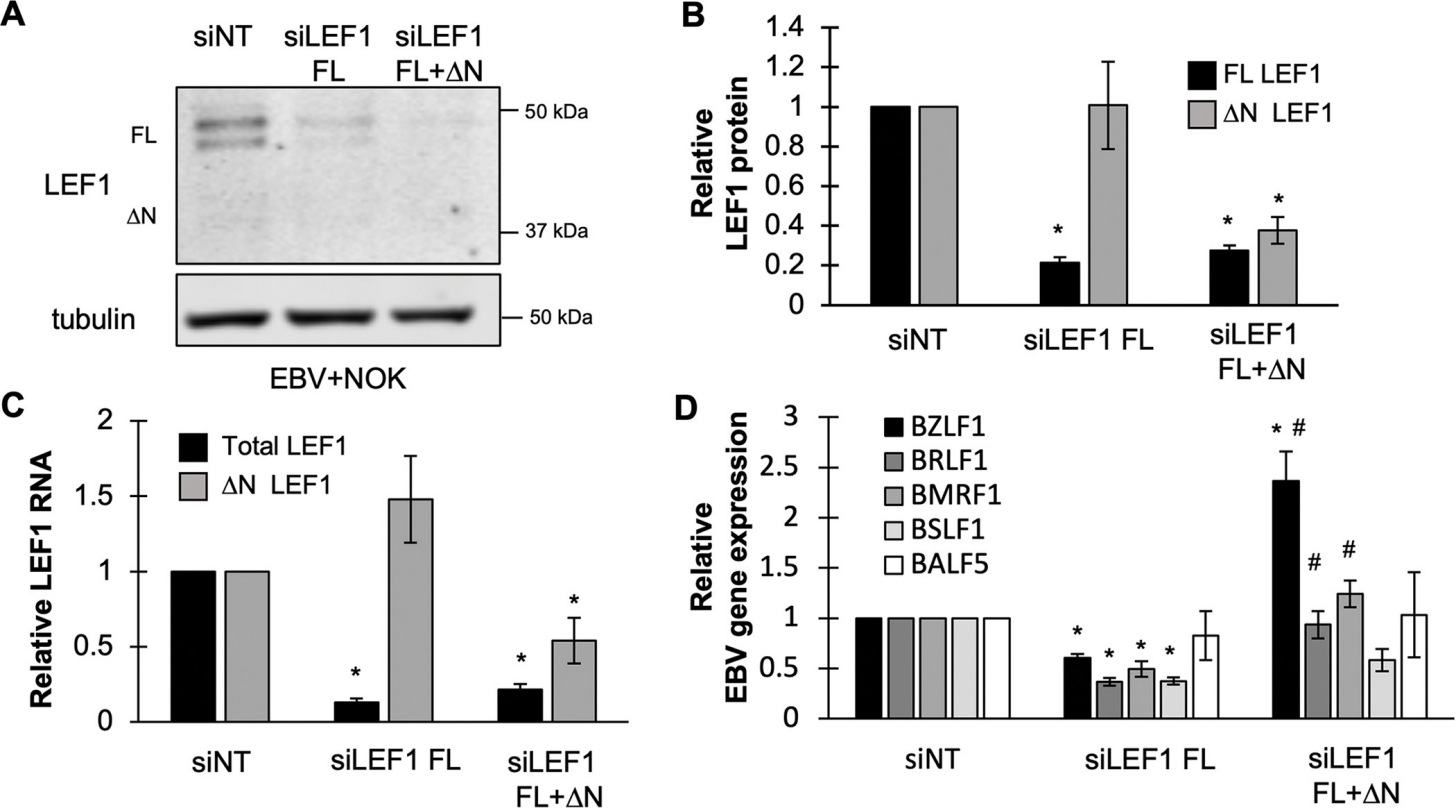
LEF1 represses lytic gene expression in an isoform-dependent manner in EBV-positive epithelial cells. **(A**) A representative immunoblot image of LEF1 protein levels in EBV+NOKs 48 hours after transfection with two independent siRNA constructs that target only full-length (siLEF1 FL) or all LEF1 isoforms (siLEF1 FL+ΔN LEF1). **(B)** Densitometry analysis of LEF1 protein levels 48 hours after siRNA transfection (n = 3). Shown are the mean and standard error of the mean. Significance was calculated using Student’s t-test. * P <0.05. **(C)** Total LEF1 transcripts (black bars) or ΔN LEF1 transcript (gray bars) were measured by RT-qPCR 48 hours after transfection of EBV+NOK with indicated siRNAs. Shown are the mean and standard error of the mean calculated from four to five independent experiments. Significance was calculated using Student’s t-test. * P <0.05 **(D)**. Analysis of EBV lytic gene transcripts levels by RT-qPCR in EBV+NOK transfected with siRNA) and induced to differentiate for 48 hours with 1.5 mM calcium and 10% serum. Shown are the mean and standard error of the mean for BZLF1 (black bars, n = 4), BRLF1 (dark gray bars, n = 3), BMRF1 (gray bars, n = 5), BSLF1 (light gray bars), and BALF5 (white bars, n = 4). Shown are the mean with error bars representing the standard error of the mean from three to five independent experiments. Significance was calculated using the Student’s t test. * is compared to the siRNA control, P < 0.01; # indicates significance of samples relative to siLEF1 FL treatment, P<0.05.

To determine the effect of LEF1 on the EBV life cycle, siRNA knockdown of LEF1 expression was performed in EBV+NOK. EBV reactivation of lytic gene expression was analyzed following calcium and serum induced differentiation. Two sets of exon specific siRNAs were used to specifically deplete either full-length LEF1 isoforms 1, 2, and 3 (siLEF1 FL) or all LEF1 isoforms including alternative-promoter derived N-terminal truncated ΔN LEF1 (siLEF1 FL + ΔN). The specificity of the siRNA targeting was validated by immunoblotting and RT-qPCR. Transfection with siRNAs targeting only full-length LEF1 isoforms reduced total LEF1 RNA transcripts and protein by approximately 80% compared to non-target controls, while ΔN LEF1 RNA levels remained intact (Fig 3A–3C). SiRNA targeting all LEF1 isoforms (siLEF1 FL + ΔN) depleted both total LEF1 and ΔN LEF1 (isoform 4) protein levels by at least 65% compared with initial levels (Fig 3A–3C).

To determine whether LEF1 binding represses EBV lytic gene transcription, we analyzed the expression of viral immediate early and early lytic genes in the context of LEF1 knockdown. We selected the following EBV genes based on confirmed LEF1 occupancy within or near coding regions and *cis* elements. The IE transactivators BZLF1/BRLF1 have a LEF1 binding site at the 3’ end of the gene and a second binding site near the BZLF1 TATA box; the viral primase BSLF1 contains a LEF1 binding site in its coding region; and the viral polymerase processivity factor BMRF1 has a LEF1 binding site in its 3’UTR. BALF5 was included as a viral lytic gene lacking a WRE motif and evidence of LEF1 binding. BALF5 transactivation has been shown to be mediated indirectly by R interacting with the cellular transcription factors USF and E2F rather than by direct DNA binding [69]. Following LEF1 depletion, EBV+NOK cells were treated with calcium and serum for 48 hours to induce lytic gene expression. Transcript levels were analyzed via RT-qPCR and normalized to the siNT control. Surprising, depletion of only LEF1 full-length isoforms, without depletion of the ΔN LEF1 isoform, showed a statistically significant decrease in BZLF1, BRLF1, BMRF1, and BSLF1 transcript levels compared to the siNT control (Fig 3D). Knockdown of all LEF1 isoforms showed the opposite trend with a statistically significant increase in BZLF1 transcripts compared to levels in the nontarget controls (Fig 3D). BRLF1, and BMRF1 also significantly increased when all LEF1 isoforms were depleted relative treatment with the siRNA targeting only full-length LEF1, while BSLF1 transcript levels remained suppressed compared to the siNT control. BALF5 transcript levels appeared not to change between non-target controls and either knockdown condition (Fig 3D). This observation was consistent with LEF1 acting in an isoform dependent manner as a repressor of EBV lytic gene expression and promoting maintenance of EBV latency.

### ΔN LEF1 antagonizes latent EBV reactivation in latently infected epithelial cells

**Fig 4:**
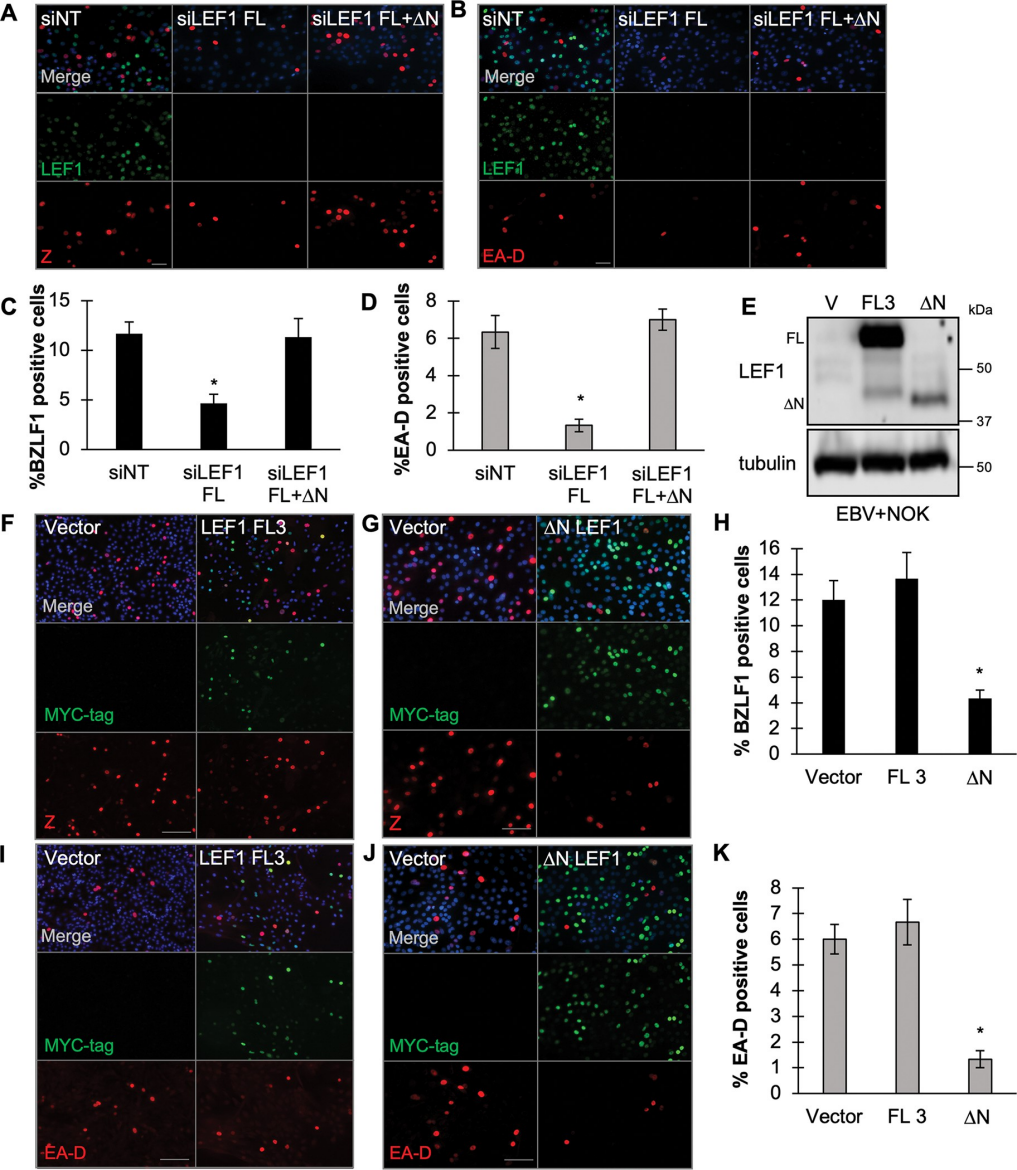
ΔN LEF1 antagonizes latent EBV reactivation in latently infected epithelial cells. **(A)** Immunofluorescence analysis in differentiation induced EBV+NOKs cells following the indicated siRNA transfection (siNT, siLEF1 FL, or siLEF1 FL+ΔN). Cells were treated with calcium and serum for 48 hours to induce differentiation. Immunofluorescence co-staining detected BZLF1 (red), LEF1 (green) and merge/Hoeschst (blue), and **(B)** EA-D (red), LEF1 (green) merge/Hoeschst (blue) (n = 3). **(C)** Quantification of the number of BZLF1 positive or **(D)** EA-D positive cells in each transfection condition (n = 3). **(E)** Forced expression of LEF1 full-length isoform 3 (FL3) or ΔN LEF1 isoform in EBV+NOK. Cells were treated with calcium and serum for 48 hours to induce differentiation. Shown is an immunoblot comparing the LEF1 protein levels in EBV+NOK stably transfected with pCMV6 vector, pCMV6 LEF1 FL3 or pCMV6 ΔNLEF1. **(F-H)**. BZLF1 positivity in EBV+NOK transfected with **(F)** vector control and pCMV6 LEF1 V3, or **(G)** vector control and pCMV6 ΔN LEF1. Cells were treated with calcium and serum for 48 hours to induce differentiation. Immunofluorescence co-staining detected BZLF1 (red), LEF1-myc tag (green) merge/Hoeschst (blue). **(H)** Percent of cells positive for BZLF1 calculated using 6 images from 3 independent experiments. Percentage of positive cells was calculated as the ratio of signal-positive cells to individual nuclei (Hoeschst). **(I-J).** EBV EA-D positivity in EBV+NOK transfected with **(I)** vector control and pCMV6 LEF1 FL3, or **(J)** vector control and pCMV6 ΔN LEF1. Cells were treated with calcium and serum for 48 hours to induce differentiation. Immunofluorescence co-staining detected EA-D (red), LEF1-myc tag (green) merge/Hoeschst (blue). **(K)** Percent of cells positive for EA-D calculated using 6 images from 2 independent experiments. Percentage of positive cells was calculated as the ratio of signal-positive cells to individual nuclei (Hoeschst). Scale bars represent 100 μm. Statistical significance was calculated with the Student’s t test. *, P < 0.05.

We next evaluated whether ΔN LEF1 was involved in the repression of EBV reactivation at the cell level. LEF1 isoforms were selectively depleted or overexpressed in the EBV+NOK cell line. The number of Z and EA-D positive cells was quantified using immunofluorescence. Selective depletion of LEF1 had no effect on spontaneous EBV reactivation (S2 Fig). We next examined, induction of viral reactivation with calcium and serum for 48 hours. When only the full-length LEF1 isoforms were depleted, the number of Z and EA-D positive cells decreased by 2.5-and 5-fold, respectively (Fig 4A–4D). In contrast, siRNA knockdown of all LEF1 isoforms, including the ΔN LEF1 isoform, restored the viral reactivation efficiency to slightly higher levels than observed in the non-target siRNA controls (Fig 4A–4D).

To demonstrate that ΔN LEF1 specifically inhibited EBV reactivation, EBV+NOK cell lines were generated that expressed a c-terminal myc tagged versions of ΔN LEF1 or full-length LEF1 isoform 3 ectopically. Overexpression of the full-length LEF1 isoform 3 and ΔN LEF1 isoform was confirmed by immunoblot (Fig 4E). Viral reactivation was analyzed after 48 hours of calcium and serum treatment in three independent experiments. Under control conditions, approximately 12% of EBV+NOK cells showed EBV reactivation being positive for BZLF1 and approximately 6% expressed EA-D. Forced expression of the full-length LEF1 isoform 3 had no effect on the percentage of cells reactivating EBV as determined by Z and EA-D protein detection (Fig 4F, 4H, 4I and 4K). In contrast, ectopic expression of ΔN LEF1 significantly reduced the number of Z and EA-D positive cells to approximately 4% and 1% positive cells, respectively (Fig 4G, 4H, 4J and 4K). In addition, we observed that EBV reactivation (Z and EA-D detection) was exclusive to cells lacking ectopically expressed, myc-tagged ΔN LEF1 protein (Fig 4G and 4J). We also compared the reactivation efficiency after transient transfection of full-length isoform 1 (FL1) to the ΔN isoform 4 (S3 Fig). Despite a low transfection efficiency, we observed that the number of LEF1 FL1+/Z+ double positive cells was 2-fold greater than BZLF1 positive cells expressing the ΔN LEF1 isoform 4 (S3 Fig). Overall, these results suggested that overexpression of ΔN LEF1 likely competed with full-length isoforms for viral genome binding and represses EBV lytic gene expression for maintenance of viral latency.

### LEF1 interference of EBV reactivation is independent of epithelial differentiation

**Fig 5:**
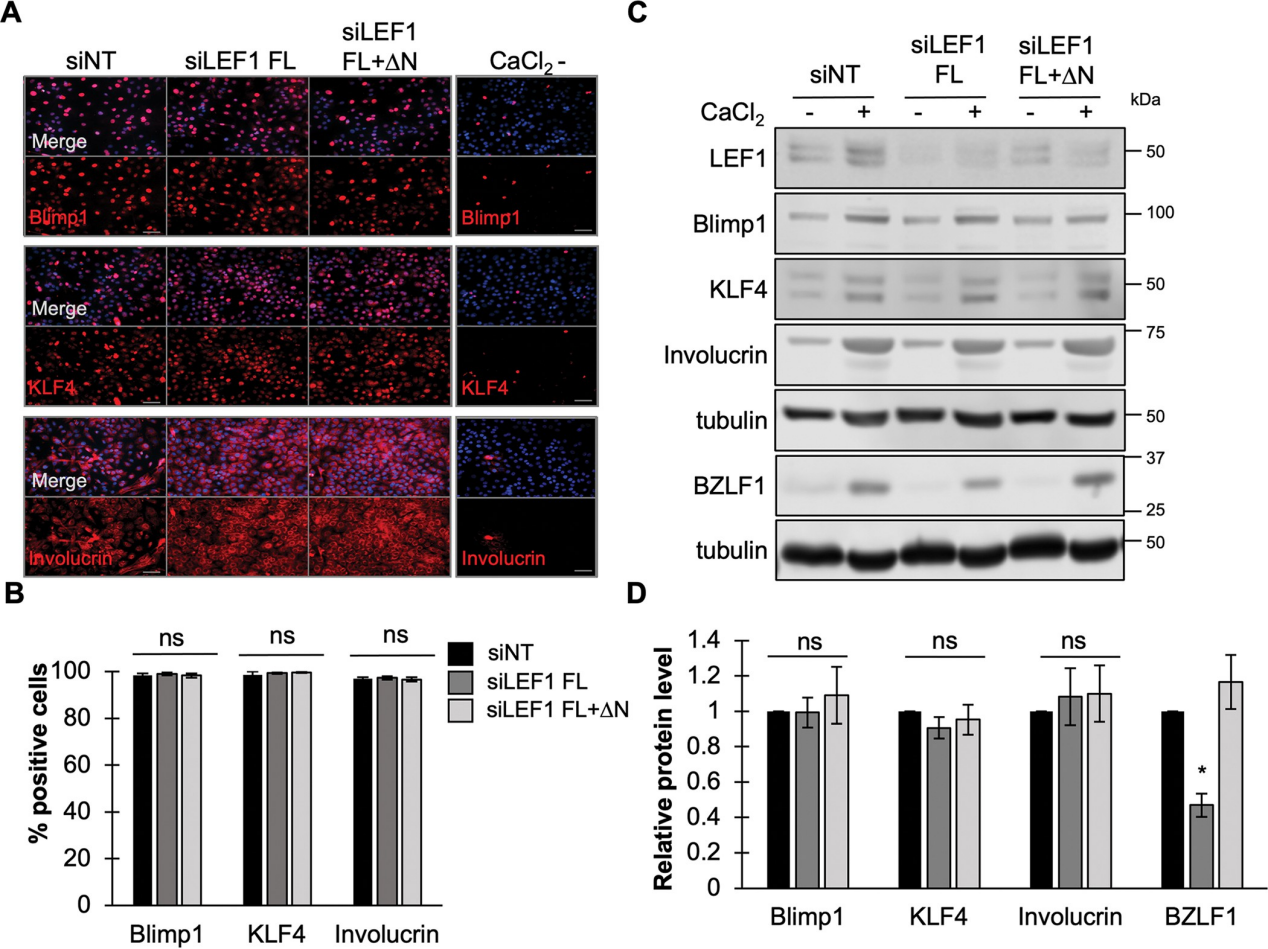
Loss of LEF1 does not alter host responsiveness to differentiation in EBV+ epithelial cells. **(A)** Immunofluorescence analysis of Blimp1/PRDM1, KLF4 or Involucrin in EBV+NOK following transfected with siNT, siLEF1 FL, or siLEF1 FL+ΔN and induced for 48 hours with calcium and serum to induce differentiation. An untreated control was also included (CaCl_2_-). Top panel detects Blimp1/PRDM1, middle panel detects KLF4, and bottom panel detects involucrin with differentiation markers shown in red and Hoeschst in blue. Scale bars represent 100 μm. **(B)** Percent of cells positive for Blimp1/PRDM1, KLF4, or Involucrin cell in each siRNA condition: siNT (black bars), siLEF1 FL (dark gray bars) and siLEF1 FL+ΔN (light gray bars). Shown are the mean with error bars depicting the standard error of the mean. 3 images were manually counted from three independent experiments. Percentage of positive cells was calculated as the ratio of signal-positive cells to individual nuclei (Hoeschst). Significance was calculated by Student’s t test. *, P < 0.05; ns, not significant **(C)** Western blot analysis of EBV+NOK transfected with indicated siRNA following 48 hours of treatment with calcium and serum to induce differentiation. Immunoblots were probed using antibodies directed against LEF1, Blimp1/PRDM1, KLF4, involucrin, BZLF1 and alpha tubulin. **(D)** Densitometry analysis for Blimp1/PRDM1, KLF4, Involucrin, and BZLF1 protein levels in the indicated transfection conditions: siNT (black bars) siLEF1 FL (dark gray bars) and siLEF1 FL+ΔN (light gray bars). Shown are the mean values relative to the untreated siNT control. Error bars depict the standard error of the mean from four independent experiments. Significance was calculated by Student’s t test; *, P < 0.05; ns, not significant.

Spontaneous lytic reactivation of EBV occurs following differentiation of B cells to plasma cells and has also been observed within differentiated strata of the oral epithelium [7,70]. Viral immediate early promoters Zp and Rp are sensitive to transcription factors associated with host differentiation including Kruppel-like factor 4 (KLF4) and B lymphocyte induced maturation protein 1/PR domain zinc finger protein 1 (Blimp1/PRDM1) in B lymphocytes and epithelial cells [10,12]. LEF1 regulates several cellular processes in lymphocytes and epithelium that include cellular proliferation, renewal, differentiation, motility and invasiveness of epithelial cells [71]. As EBV reactivation is tuned to differentiation signals, we examined whether perturbations in LEF1 altered the host cell differentiation response to calcium/serum treatment. The protein levels of KLF4, BLIMP1, and the early differentiation marker involucrin were quantified by immunofluorescence and immunoblot analysis [72,73]. Over 95% of treated EBV+NOK were positive for KFL4, Blimp1/PRDM1, and involucrin indicating a robust stimulation of differentiation (Fig 5A and 5B). In either LEF1 knockdown condition, EBV+NOK showed no significant change in KLF4, Blimp1/PRDM1, or involucrin cell positivity (Fig 5A and 5B) or protein levels (Fig 5C and 5D) relative to the NT control. However, BZLF1 (Z) transcript and protein levels were reduced only when full-length LEF1 isoforms were depleted, with a full recovery of BZLF1 (Z) transcript and protein levels observed when both full-length and ΔN LEF1 isoforms were depleted (Figs 3D, 5C and 5D). In addition, EBV+NOK ectopically expressing the ΔN LEF1 isoform showed no change in the percentage of cells positive for KLF4, Blimp1/PRDM1, and involucrin relative to vector control following calcium/serum treatment (S5 Fig). Taken together, these results indicated that the calcium-induced differentiation response was not dependent on LEF1.

### ΔN LEF1 isoform engages the latent EBV genome in Akata BL cells

**Fig 6:**
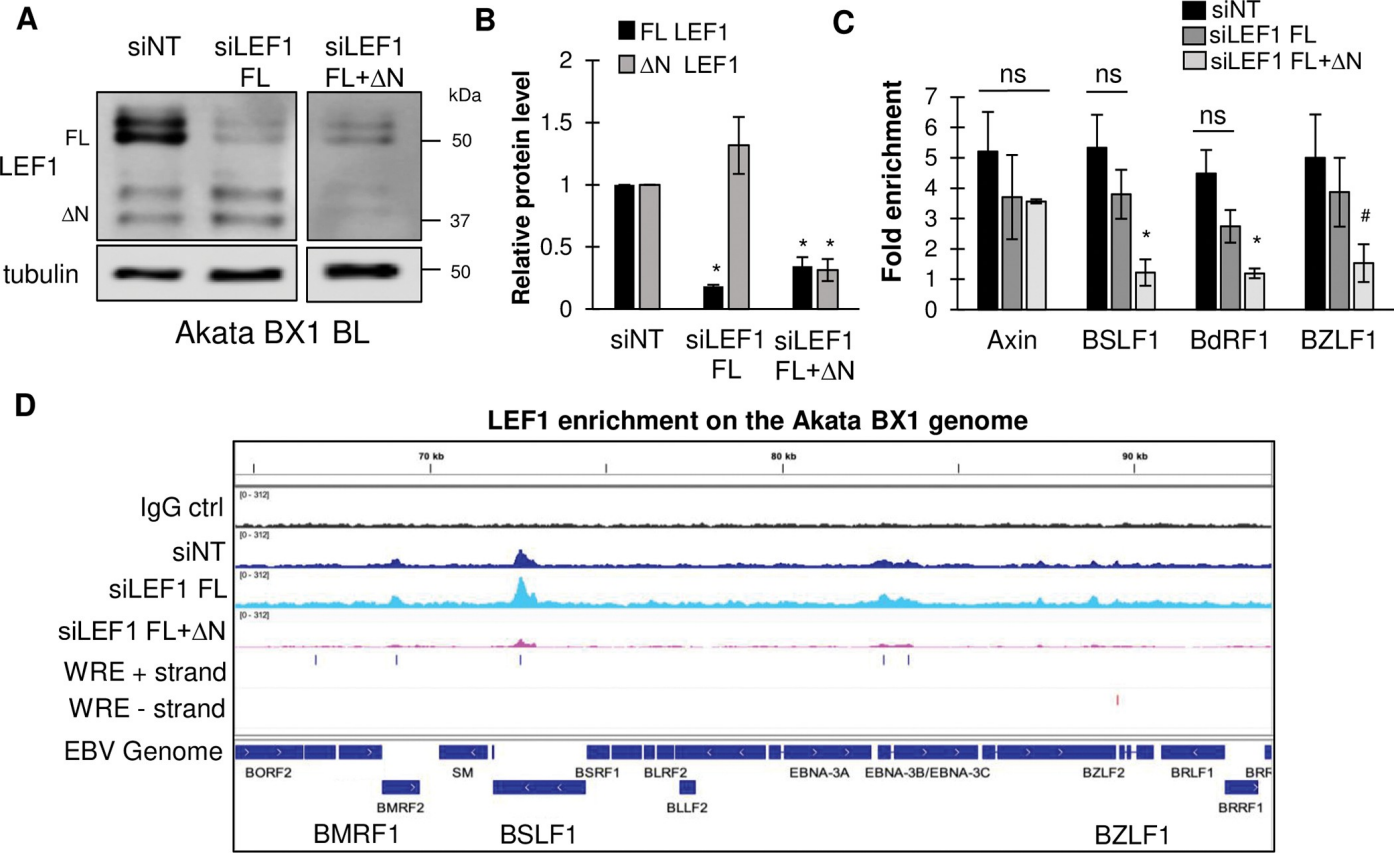
ΔN LEF1 engages the of latent EBV genome in Akata B cells. **(A)** Western blot confirmation of LEF1 isoform knockdown in Akata BX1 BL samples utilized for CUT&RUN-seq analysis. Immunoblots were probed with antibodies specific to LEF1 and alpha tubulin **(B)** Densitometry analysis of LEF1 protein levels following the indicated siRNAs. Shown is the mean relative to the siNT controls for LEF1 isoform 1 (black bars) and the ΔN LEF1 (gray bars from two to five independent experiments **(C)** CUT&RUN-qPCR using primers specific for LEF1 binding sites Axin2 as a positive control, BSLF1, BdRF1, and BZLF1. Shown is the mean with error bars representing the standard error of the mean from four independent experiments. Significance was calculated by Student’s t test; *, P < 0.05; ^#^, P< 0.54; ns, not significant **(D)** Representative LEF1 binding tracks from CUT&RUN-seq in Akata BX1 BL following transfection with siNT (dark blue), siLEF1 FL (light blue), or siLEF1 FL+ΔN (pink) (n = 2). IgG control is shown in black. The predicted WRE (CTTGWWS) motifs are marked on the plus DNA strand (blue) and minus DNA strand (red). The EBV genomic region is shown on the bottom track.

CUT&RUN was performed in Akata BX1 BL to analyze LEF1 binding profiles following isoform specific siRNA depletion. In the Akata BX1 BL, electroporation of siLEF1 FL siRNA depleted full-length LEF1 protein levels (50 kDa) by 80% without affecting the ΔN LEF1 (37 kDa) protein levels relative to the siNT control (Fig 6B). Electroporation of the siRNA targeting both full-length and ΔN LEF1 resulted in a decrease of at least a 65% in both full-length and ΔN LEF1 protein (Fig 6A and 6B). CUT&RUN-qPCR was used to quantify the relative LEF1 enrichment at various EBV regions in each knockdown condition (Fig 6C). In the non-target siRNA controls, we observed a 4 to 5-fold enrichment in LEF1 occupancy at BSLF1, BdRF1, BZLF1, and Axin2 sites relative to the IgG controls (Fig 6C). Depletion of only the full-length LEF1 isoforms, without affecting ΔN LEF1 protein levels, resulted in a slight drop in LEF1 enrichment at Axin2, BSLF1, and BdRF1, and BZLF1 compared to the non-target siRNA control; however, this decrease was not statistically significant (Fig 6C). In contrast, knockdown of all LEF1 isoforms resulted in a significant loss in LEF1 binding at BSLF1, BdRF1, and BZLF1 (Fig 6C). LEF1 binding at the cellular Axin2 promoter was not significantly diminished following depletion of all LEF1 (Fig 6C). CUT&RUN sequencing confirmed LEF1 binding across the EBV genome after treatment with the siRNA that only targeted the full-length LEF1 (siLEF1 FL) similar to that observed for the non-target control. Robust LEF1 binding was observed at the BSLF1. When all LEF1 isoforms were depleted, LEF1 binding was further depleted throughout the EBV genome, most evident at BSFL1 (Fig 6D). These results indicated the ΔN LEF1 isoform engaged the latent EBV genome. However, we were unable to determine 1) whether the ΔN LEF1 isoform preferentially bound to the latent EBV genome or 2) whether depletion of full-length LEF1 isoforms allowed for increased occupancy of the ΔN LEF1 isoform on the latent EBV genome.

### LEF1 regulates EBV lytic gene expression in epithelial cells via repressive histone deacetylation

**Fig 7:**
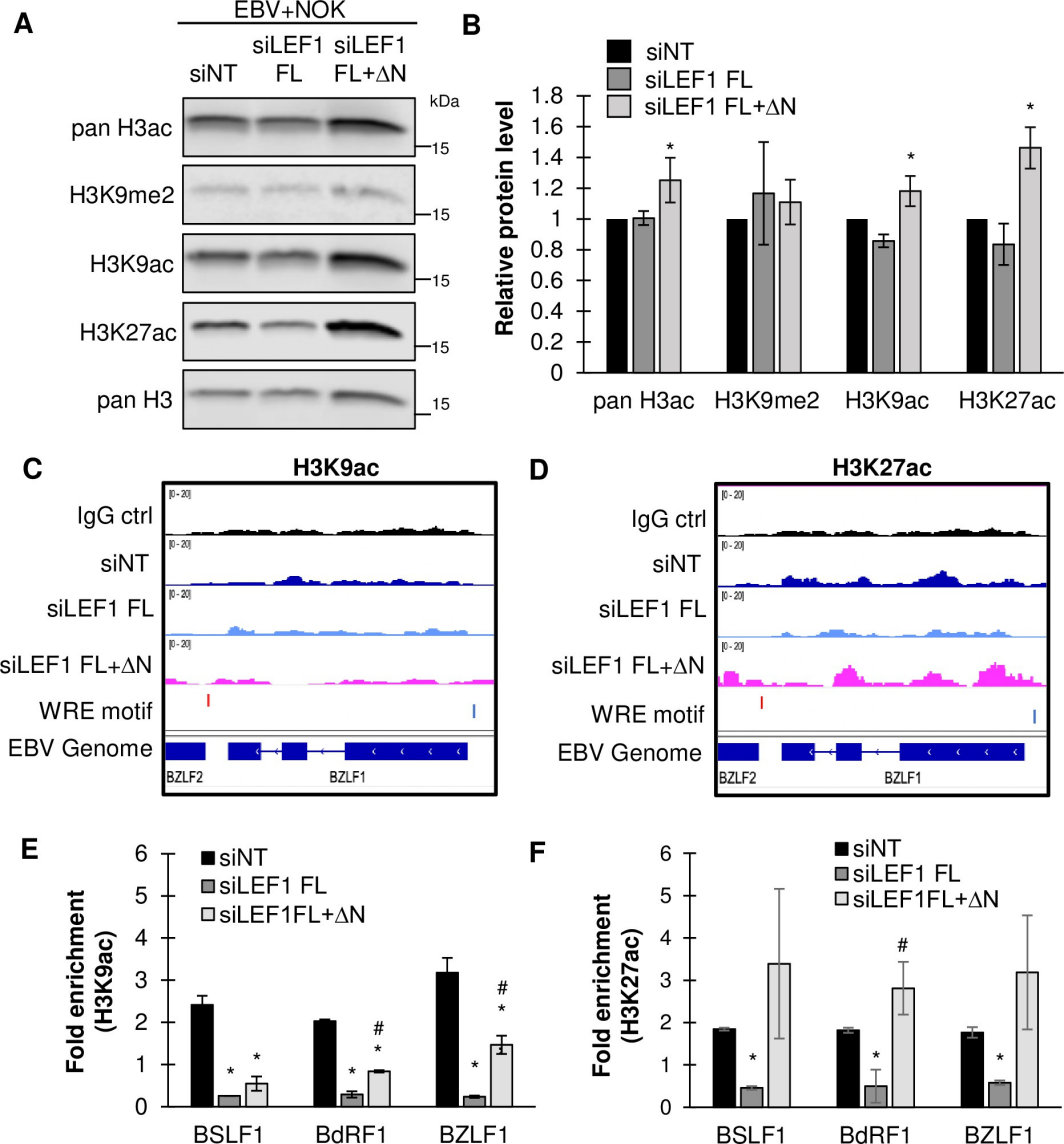
ΔN LEF1 deacetylates the latent viral genome in epithelial cells. **(A)** Western blot analysis of histone acetylation in EBV+NOK transfected with indicated siRNA. Immunoblots were probed using antibodies directed against pan acetylated H3, H3K9me2, H3K9ac, H3K27ac, and pan H3. **(B)** Densitometry analysis of histone acetylation marks was calculated relative to NT control. Shown are the mean with error bars representing the standard error of the mean from three independent experiments. **(C)** H3K9ac and **(D)** H3K27ac directed CUT&RUN-seq in EBV+NOK following transfection with indicated siRNA. Bedgraph tracks from a representative experiment are shown depicting siNT (dark blue), siLEF1 FL (light blue), and siLEF1 FL+ΔN (pink) conditions (n = 3). The WRE motifs for BZLF1 are shown in blue (plus DNA strand, degenerate WRE) near the TATA box and in red (minus DNA strand) at the 3’ end of the gene. **(E)** H3K9ac and **(F)** H3K27ac directed CUT&RUN-qPCR analysis with primers spanning predicted WRE motifs. Shown are the mean with error bars representing the standard error of the mean from two independent experiments. Significance was calculated by Student’s t test. *, P < 0.05 relative to non-target control.^#^, P<0.05 depicts significance between siLEF1 FL and siLEF1 FL+ΔN treated groups.

LEF1 has no intrinsic transactivation domain and thus mediates transcriptional regulation through interaction with several co-factors including the co-activator beta-catenin and co-repressors Groucho/Transducin-like enhancer of split (Gro/TLEs) and Mothers Against DPP Homolog (SMAD) family members [60]. ΔN LEF1 lacks the N-terminal beta-catenin binding domain yet retains conserved sites for co-repressor binding within the CRD and HMG-DBD [74,75]. Importantly, Wnt co-repressors are known to recruit histone deacetylases (HDACs) [76,77]. Also, LEF1 has an intrinsic HDAC activity mapped to a region between amino acids residues 187–216, which is present in all isoforms [55]. It is well established that epigenetic modifications such as histone acetylation and chromatin accessibility regulate the EBV latent/lytic switch. To characterize LEF1 regulated histone acetylation in latently infected EBV+NOK, the abundance of specific histone 3 (H3) acetylation marks was analyzed via immunoblotting following LEF1 knockdown. Depletion of both full-length and ΔN LEF1 isoforms showed a global and statistically significant increase in total acetylated H3 (pan H3 Ac) and the specific H3 lysine acetylation marks, H3K9ac and H3K27ac compared to the siNT control (Fig 7A and 7B). Importantly, knockdown of LEF1 did not alter the abundance of repressive histone mark H3K9me2 indicating LEF1 specifically regulates histone acetylation (Fig 7A and 7B). Interestingly, a slight decrease in the acetylation of H3K9ac and H3K27ac was observed in siLEF1 FL knockdown (retaining ΔN LEF1). Using CUT&RUN, we examined histone acetylation across the latent EBV chromatinized genome in the context of LEF1 knockdown. We observed diminished histone acetylation of the entire viral genome following depletion of only the full-length LEF1 isoforms in EBV+NOK (Figs 7C, 7D and S6). Loss of both ΔN LEF1 and full-length LEF1 increased H3K9 and H3K27 acetylation across the EBV genome (Figs 7C, 7D and S6). We then used CUT&RUN-qPCR to quantify histone acetylation at LEF1 binding sites. A significant decrease in both H3K9ac and H3K27ac at BSLF1, BdRF1, and BZLF1 was observed following siLEF1 FL transfection (Fig 7E and 7F) correlating ΔN LEF1 with the histone deacetylated state of the EBV genome. Knockdown of both full-length and ΔN LEF1 isoforms had distinct effects when profiling H3K9 versus H3K27 acetylation. For H3K9 acetylation, depletion of all LEF1 isoforms slightly increased levels when compared loss of only full-length LEF1 isoforms, but H3K9 levels were not restored to the levels observed in non-target controls (Fig 7E). For H3K27 acetylation, depletion of all LEF1 isoforms increased H3K27 acetylation at or above the levels in the non-target controls (Fig 7F). These results suggest preference of ΔN LEF1 in H3K27 deacetylation, an epigenetic mark associated with promoter and enhancer activity (Fig 7E and 7F). Taken together, these data implicate ΔN LEF1 as a regulator of EBV latency via deacetylation of viral chromatin and subsequent lytic gene silencing.

### LEF1-EBV genome engagement is maintained following lytic cycle induction

**Fig 8:**
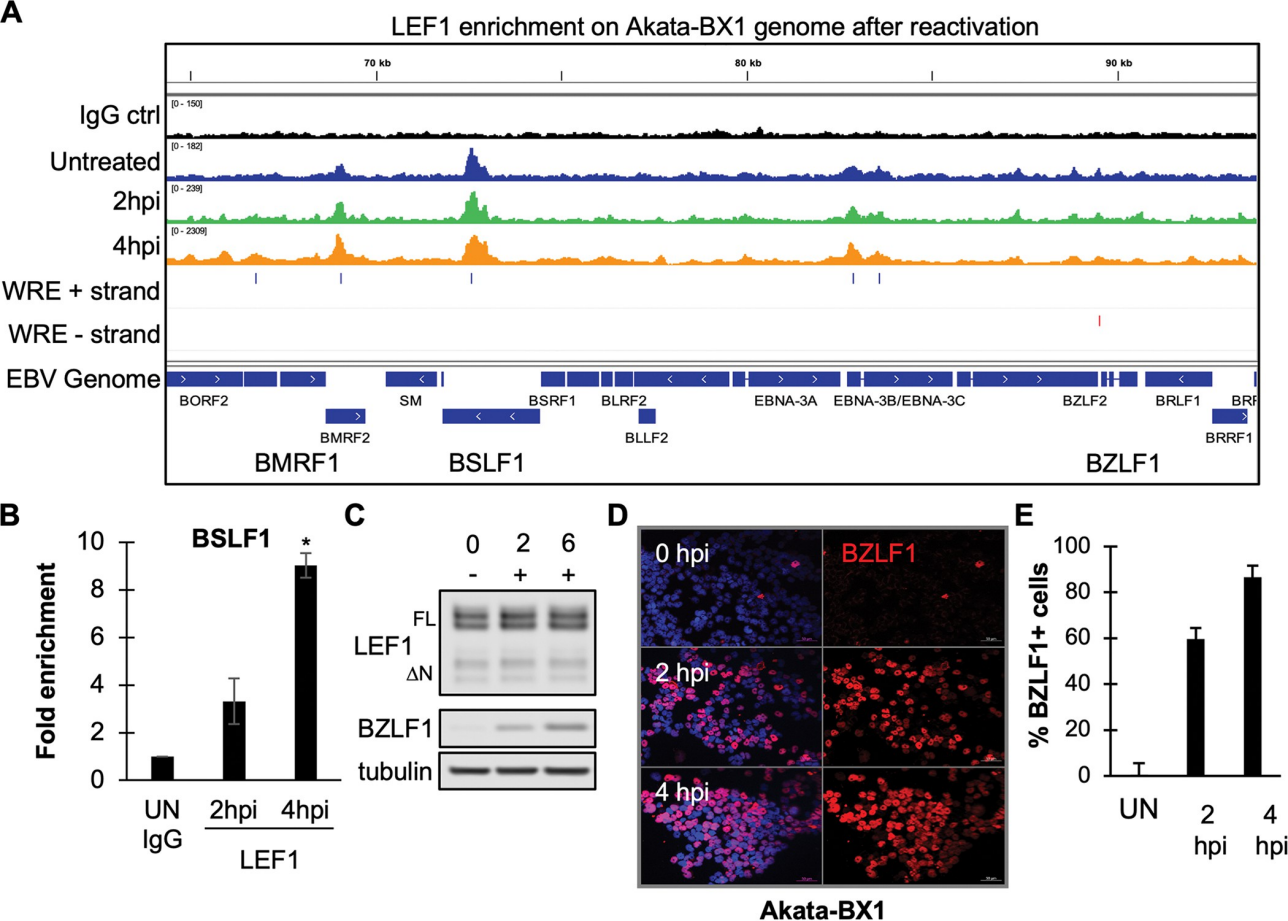
LEF1 genome binding is maintained following EBV reactivation in Akata BL. **(A)** CUT&RUN-seq analysis of LEF1 binding following reactivation of latently infected Akata BX1 BL. Viral reactivation was induced by B cell receptor (BCR) ligation with anti-human IgG. Representative bedgraph tracks are shown for uninduced cells (blue), 2 hours (green), or 4 hours (orange) post treatment. (n = 2). **(B)** Cut&Run-qPCR analysis depicting fold enrichment relative to control IgG for LEF1 binding at BSLF1 at indicated time points. (n = 2) **(C)** Western blot analysis of LEF1 protein levels following EBV reactivation at indicated time points in BCR-ligated Akata BX1.–indicates uninduced cells prior to induction (time 0); + was treated with IgG for 2 and 6 hours post infection (hpi) **(D)** Immunofluorescence analysis of EBV reactivation efficiency at indicated time points, BZLF1 (red), merge/Hoeschst (blue). Scale bars indicate 50 μm. **(E)** Quantification of BZLF1 positive cells at indicated time points.

EBV is capable reactivating in the presence of endogenous LEF1 protein levels despite the potent repressive activity of ΔN LEF1 (Fig 4F–4K). Therefore, we next examined whether LEF1 occupancy on the EBV genome was altered during viral reactivation. Using CUT&RUN-seq, LEF1 occupancy of untreated Akata BX1 BL cells was compared to LEF1 occupancy at early times following viral reactivation induced by B cell receptor ligation. Surprisingly, LEF1 remained associated with the EBV genome up to 4 hours after viral reactivation (Fig 8A). CUT&RUN-qPCR analysis confirmed LEF1 binding at BSLF1. Intriguingly, compared to IgG controls, LEF1 enrichment at BSLF1 increased from 3-fold enrichment in uninduced samples to 9-fold at 4 hours post reactivation. LEF1 protein levels remained unchanged up to 6 hours post induction (Fig 8B and 8C). As a control to ensure efficient viral reactivation was maintained across each experimental condition, BZLF1 cell positivity was examined using immunofluorescence analysis (Fig 8D and 8E). At 2 hours post induction, approximately 70% of the Akata BL cells were BZLF1 positive, increasing to 80% at 4 hours post reactivation (Fig 8D and 8E). These results indicated that EBV reactivation can overcome LEF1 repressive activity even though LEF1 remains bound to the viral genome.

## Discussion

In this study, hTERT-immortalized normal oral keratinocytes (NOK) and Burkitt Lymphoma cells latently infected with EBV were investigated to determine the role of Wnt signaling effector LEF1 in the EBV life cycle. We observed that LEF1 was a transcriptional repressor of viral lytic gene capable of antagonizing viral reactivation in epithelial cells and maintaining the EBV genome in a histone deacetylated state. In addition, we observed that this activity was dependent on an alternative promoter derived isoform of LEF1 lacking the N-terminal beta-catenin binding domain, ΔN LEF1.

LEF1 belongs to the TCF family of transcription factors that share a highly conserved high mobility group box (HMG)/DNA binding domain that interact with the WRE consensus sequences [56].The EBV genome encodes over 20 conserved, putative WRE core motifs between prototype type 1 and type 2 viral strains. Thus, an interplay between the TCF/LEF1 transcription factors may coordinate viral gene expression directly through binding of the viral genome or indirectly through host gene regulation affecting cell cycle and differentiation. LEF1 was detected in various latently infected BL and epithelial cell lines in the presence or absence of other TCF proteins (Fig 2C). Whether the other TCFs also bind the EBV genome has yet to be investigated. Here we demonstrate that LEF1 engages several WRE motifs across the EBV genome that were close matches to the WRE core consensus sequence.

LEF1 binding on the EBV genome occurred mostly within exons/open reading frames of genes. In addition, LEF1 binding was observed at the TATA box region of BZLF1 and at the 3’ end of the gene which also overlaps with BRLF1. The most robust binding occurred within the BSLF1 open reading frame, which encodes an extended WRE consensus sequence. The ΔN LEF1 shares a DNA binding domain with full-length isoforms [62] and siRNA depletion of full-length LEF1 confirmed that the N-terminal truncated ΔN LEF1 isoform bound the EBV genome. Interestingly, DNA binding profiles of ΔN LEF1 were similar in both pattern and enrichment level as observed when all isoforms were present, suggesting that ΔN LEF1 may be the predominant isoform binding the EBV genome (Fig 6C and 6D). However, our study cannot exclude the possibility that depletion of the LEF1 full-length isoforms or overexpression of ΔN LEF1 skewed the steady state levels towards ΔN LEF1 binding to the EBV genome.

We have specifically identified the ΔN LEF1 isoform as a negative regulator of EBV lytic gene expression in the EBV+NOK (Fig 3), evidenced by the suppression of the immediate early genes BZFL1 and BRLF1 and early genes BMRF1 and BSLF1. An early gene encoding the viral polymerase BALF5, not occupied by LEF1, was not transcriptionally affected in any of the knockdown conditions (Fig 3D), indicating that ΔN LEF1 repressive activity associated with DNA binding in the vicinity of the affected genes. In the Akata BX1 BL cell line, we have been unable to recapitulate the repressive effects of ΔN LEF1 on EBV reactivation. This may be due to incomplete depletion of all LEF1 isoforms following knockdown. Some remnant LEF1 binding was still evident in the Akata BX1 BL cells following knockdown of all LEF1 isoforms (Fig 6C and 6D). In addition, LEF1 function may be dependent on the genetic and epigenetic cellular context. Expression of TCF7 protein (TCF1) is evident in Akata BX1 BL and whether TCF7 (TCF1) counters the repressive effects of ΔN LEF1 needs to be further investigated. In addition, BL carry a hallmark chromosomal translocation resulting in the overexpression of the c-Myc transcription factor, a negative regulator of the EBV latent/lytic switch [78]. C-Myc is a known transcriptional target of LEF1; however, crosstalk has also been shown where c-Myc activates the expression of LEF1 [79]. How the dysregulated expression of c-Myc in BL affects LEF1 will need to be further examined.

LEF1 is expressed as multiple isoforms with distinct functions associated between full-length and truncated LEF1 isoforms. Full-length isoforms mediate growth and Wnt/beta-catenin responsiveness, while ΔN LEF1 acts as a dominant negative feedback switch and is associated with genes involved in stemness [80]. In Wnt signaling pathway, LEF1 interactions with beta-catenin transcriptionally activate Wnt regulated genes. LEF1 also mediates transforming growth factor beta (TGFB) signaling responses by interacting with the SMAD family of transcriptional factors [47]. Based on the known functions of ΔN LEF1, repression of EBV reactivation may be related to a lack of beta-catenin (an activator of LEF1) recruitment to the EBV genome. In EBV latently infected cells, increased beta-catenin levels are observed [81–83]. Recent studies with human herpes simplex virus 1 have shown that beta-catenin is required for efficient productive replication [84]. In addition, LEF1 interactions with other transactivators may also be involved. TGF-beta is a potent inducer of EBV reactivation mediated by SMAD binding to the BZLF1 promoter [85]. LEF1 interaction with SMADs is mediated by the HMG box, which is present in ΔN LEF1. Whether LEF1 interactions with beta-catenin and/or SMADs is required for EBV lytic replication needs to be further examined.

LEF1 repressive transcriptional activity is also potentiated by interaction with negative regulators Groucho/TLE that recruit HDACs to facilitate chromatin condensation [86]. In addition, LEF1 shares homology with HDAC 8 [87–89] and has been shown to possess an intrinsic HDAC domain [55]. ΔN LEF1 retains both the Groucho/TLE interaction and intrinsic HDAC domains [53,55,56]. H3K9 and H3K27 acetylation are epigenetic marks associated with transcriptionally active promoters and enhancers, respectively [90–92]. Histone acetylation increases chromatin accessibility facilitating the recruitment of transcription complexes to genomic and viral DNA [93]. HDAC inhibitors are potent inducers of EBV reactivation via increased histone acetylation of the immediate early promoters [94]. When full-length LEF1 isoforms were depleted, without affecting ΔN LEF1 levels, histone H3K9 and H3K27 acetylation decreased globally and at regions on the EBV genome compared to non-target controls (Fig 7B–7F). Depletion of all LEF1 isoforms, including ΔN LEF1, resulted in a predominant increase in H3K27 acetylation globally and across the EBV genome. The H3K9me2 repressive histone methylation mark was unaffected by ΔN LEF1 depletion, indicating a specific role for LEF1 in regulating chromatin acetylation. Similar effects on histone acetylation were observed in CD8+T from TcfF7/Lef1 knockout mice with specific increases in H3K27 and H3K9 acetylation observed not only at Tcf7/Lef1 target genes but also at several hundred non-target sites [55]. Mutation of the Tcf7 HDAC domain increased H3K27ac when compared to wild-type control. Thus, the increase in histone acetylation across of the EBV genome following depletion of LEF1 supports a role for ΔN LEF1 in epigenetically maintaining the EBV genome in a condensed, histone deacetylated state.

LEF1 DNA binding can induce a sharp DNA bend and form DNA loops. LEF1 looping was shown to occur at COX2 and MMP13 genes where LEF1 binding to 3’ regions interacted with transcription factors (AP1, NF-kB) in the gene promoter [95–97]. Similar LEF1 DNA looping may occur at BZLF1 where LEF1 binding was evident at the 3’end and promoter regions, known to be occupied by factors that also interact with LEF1 (SMADs). Such LEF1 interactions may influence chromatin looping between BZLF1 and OriLyt required for viral reactivation [78]. Moreover, LEF1 may cooperate with CTCF, a known regulator of chromatin loop formation important for establishing distinct 3-dimensional genome conformations in EBV latency types I and III [98]. CTCF also associates with the BZLF1 promoter and within BSLF1 in proximity to LEF1 binding motifs [99–101]. Such LEF1 looping interactions could influence the 3-dimensional conformation of the EBV genome enforcing transcriptional control during latency and reactivation [78].

Induction of viral reactivation in Akata BX1 BL appeared to not to affect LEF1 binding to the EBV genome (Fig 8). This observation was similar to CTCF remaining bound to the EBV genome at early time points post reactivation, supporting the notion that LEF1 binding may not be sufficient for repression of EBV reactivation [102]. Preliminary findings suggest that LEF1 protein levels decrease at later times post reactivation without affecting LEF1 transcript levels. Thus, LEF1 may acquire post-translational modifications that disrupt its repressive activity during EBV reactivation.

## Conclusion

**Fig 9:**
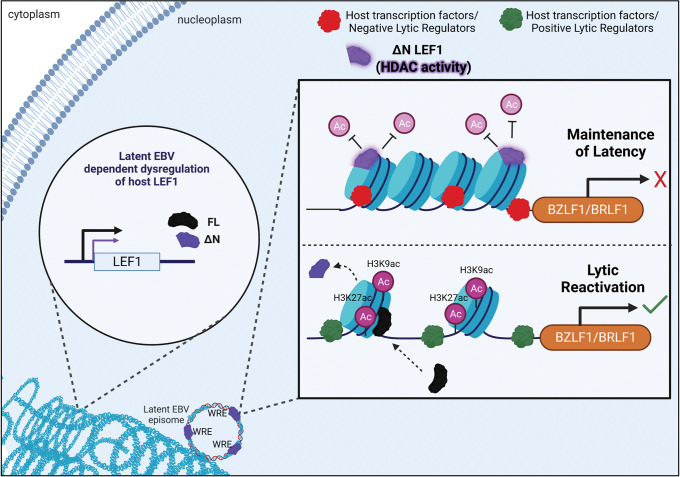
ΔN LEF1 maintains Epstein-Barr virus latency via repressive deacetylation of viral chromatin. EBV manipulates host epigenetic machinery to both regulate the viral life cycle and alter cellular function. Increased protein levels of the Wnt responsive transcription factor LEF1 are observed in EBV latent infections as well as in various EBV-associated cancers harboring latent EBV. LEF1 gene encodes various protein isoforms that includes a full-length LEF1 isoforms (black protein, FL) and an alternative promoter derived N-terminal truncation (purple protein, ΔN) that lacks the beta-catenin binding domain. The EBV genome encodes over 20 Wnt response elements that serve as binding sites for TCF/LEF1 transcription factors. Although several host transcription factors act as negative (red protein) or positive (green protein) effectors of viral reactivation, here we identify LEF1 as an epigenetic regulator of viral reactivation that aids in the maintenance of latency. ΔN LEF1 engages these sites on the latent EBV and represses immediate early (IE) genes BZLF1 and BRLF1 expression and viral reactivation (magnification square). The overabundance of ΔN LEF1 maintains the IE promoter in a histone deacetylated state (H3K9/H3K27) (light magenta circles/upper panel) enforcing the maintenance of EBV latency. Genetic ablation of LEF1 promotes H3K9/H3K27 hyperacetylation and chromatin accessibility of the viral genome to viral and host transcriptional regulators.

The findings presented provide evidence that host transcription factor LEF1 acts as a negative regulator of EBV lytic cycle reactivation in both latently infected B lymphocytes and epithelial cells. Here we show that N-terminal truncated LEF1, ΔN LEF1, binds latent EBV genomes and mediates transcriptional silencing of critical lytic genes and deacetylation of viral chromatin (modeled in **Fig 9**). Recognition of LEF1 as a regulator of the EBV life cycle will provide greater understanding of latent EBV-associated malignancies and the processes by which the virus establishes and maintains persistence within its host.

## Materials and methods

### Cell culture

Human telomerase reverse transcriptase (hTERT)-immortalized normal oral keratinocytes (NOK; gifted by Karl Munger) [103] and EBV+NOK were maintained in 1x Keratinocyte serum free medium (Gibco #10724–011) supplemented with human recombinant EGF and bovine pituitary extract (Gibco # 37000–015). EBV+NOK cell line was infected with the recombinant EBV (Akata BX1) as previously described [27] and maintained via addition of 50 μg/mL G418 sulfate (neomycin). For viral reactivation and cellular differentiation, KSFM was supplemented with 10% FBS and 1.2 mM calcium chloride. The Akata BX1 Burkitt lymphoma (BL) cell line carries a recombinant EBV where BXLF1 is replaced with a GFP/Neomycin resistance cassette [104]. The Akata BX1 BL cell line was grown in RPMI 1640 with glutamine (Corning # 10-040-CV) supplemented with 10% fetal bovine serum (FBS) and 350 μg/mL G418 sulfate. For viral reactivation, 5x10^5^ cells/mL AkataBX1 BL cells were induced by addition of 100 μg/mL goat anti-human IgG (Jackson ImmunoResearch Affinipure F(ab^’^)_2_ 109-006-003) in RPMI1640 supplemented with 10% serum. All cell lines were maintained in a humidified 37°C, 5% CO2 incubator.

### ChIP-qPCR

2 x 10^6^ Akata BX1 or 80% confluent EBV+NOK were fixed for 7 minutes in 1% formaldehyde and quenched with 125 mM Glycine for 5 minutes at room temperature. Cell nuclei were isolated by lysis in buffer containing 5 mM PIPES pH 8.0, 85 mM KCl, 0.5% NP 40, 1X Halt proteinase inhibitor cocktail and 1mM PMSF, pelleted via centrifugation at 1000 rpm for 10 minutes at 4°C, resuspended in 500 μL nuclei lysis buffer (50 mM Tris-HCL pH 8.0, 10 mM EDTA pH 8.0, 1% SDS, 1X Halt proteinase inhibitor cocktail and 1mM PMSF) and sonicated using a Diagenode Biorupter Plus for 10 cycles (30 sec ON/30 sec OFF; high setting) at 4°C. Sonication was continued for a total of 50 cycles. Sonication efficiency was analyzed using of 50 μL of clarified sonicated chromatin; a 5μL aliquot was used as input control. 950 μL ChIP dilution buffer (950μL) (0.01% SDS, 1.1% Triton-X-100, 1.2 mM EDTA pH 8.0, 16.7 mM Tris-HCl pH 8.1, 167 mM NaCl, 1X Halt proteinase inhibitor cocktail and 1mM PMSF) was combined with 50 μL clarified sonicated chromatin and incubated with 1 μg antibody overnight at 4°C. Immunoprecipitation was performed by adding 20 μL Dynabead Protein G (Invitrogen) magnetic beads to each sample and incubating at 4°C for 2 hours. Beads were washed sequentially in RIPA (150 mM NaCl), RIPA (500 mM NaCl), and RIPA 500mM LiCl. Both IP and input samples were re-suspended in 100 μL 1XTE with 1% SDS, 0.2M NaCl, and 1mg/mL proteinase K, and incubated at 55°C for 2 hours followed by overnight incubation at 65°C to de-crosslink samples. DNA was purified via phenol:chloroform:isoamylalcohol (25:24:1) extraction and isolated by overnight ethanol precipitation at -20°C with 2μg added as a carrier. DNA pellets from IP samples were re-suspended in 50 μL 1XTE and input DNA pellets were dissolved in 250 μL 1XTE. ChIP/Input ratios were calculated based on purified input DNA representing 2% of the chromatin in each IP reaction.

### CUT&RUN

CUT&RUN experiments [66] were carried out using the CUT&RUN Assay kit (#86652) from Cell Signaling Technology (protocol no. 1884). LEF1 analysis was performed with native chromatin (no formaldehyde crosslinking), while histone acetylation analysis included formaldehyde crosslinking for 90 seconds with 0.1% formaldehyde followed by quenching with 135 mM Glycine for 7 minutes. Concanavalin A magnetic beads were used to capture 150,000 Akata BX1 BL or 250,000 EBV+NOKs per sample; the cells were then incubated with 500 ng primary antibody in 100 μL antibody buffer for 12 hours. Input samples were generated by the lysis of equivalent cell numbers and DNA was isolated after lysis as performed for IP samples. For LEF1 knockdown samples and in EBV reactivation experiments, 0.2 mg donkey-anti rabbit secondary antibody (A16037, Thermo) was added and incubated 1 hour at 4°C following the primary antibody. Samples were washed as indicated for the primary antibody. Binding of pAG-micrococcal nuclease, DNA digestion, and diffusion was followed as specified in the protocol. *S*. *cerevisiae* Spike-In DNA (50 pg/sample) was added with the STOP buffer for sample normalization, and enriched chromatin fractions were collected after incubating at 37°C for 20 min, followed by proteinase K treatment as indicated. DNA was purified by phenol:chloroform:isoamylalcohol (25:24:1) extraction and isolated by ethanol precipitation incubated at -80°C for 1 hour with 2 μg glycogen added as carrier. DNA was dissolved in 50 μL 0.1× TE (1 mM Tris-HCl pH 8.0, 0.1mM EDTA). For qPCR analysis, 1 μL of IP or input samples were loaded per well/reaction. Antibodies used for LEF1, H3K9ac, H3K27ac, and BZLF1 are listed in the S2 Table.

### CUT&RUN-qPCR

Using the 50 μL enriched chromatin CUT&RUN sample as starting material, nucleosomal fragments were further enriched by performing DNA fragment size exclusion prior to qPCR analysis. SPRIselect beads (Beckman Coulter) were used to perform a “Right-Side Size Selection”. SPRIselect beads (0.7x volume) were added to each sample (35 μL beads) to bind DNA fragments larger than 300 bp. Beads were pelleted and supernatant containing fragments less than 300 bp was combined with 1.2x volume beads (55 μL beads). Pelleted beads were washed with 180 μL 85% ethanol. DNA was eluted in 20 μL water. DNA samples were amplified on a 7500 FAST Applied Biosystems thermocycler using Luna Universal qPCR master mix (NEB) and 300 nM primers in each reaction (S3 Table). DNA (1 μL) was added to 14 μL master mix per reaction (15 μL total). Thermocycling parameters were performed with an initial denaturation at 95°C for 3 minutes, followed by 40 cycles of denaturation at 95°C for 15 seconds and annealing/extension at 60°C for 1 minute. Ct values derived from antibody immunoprecipitations were compared to those derived from IgG controls or siNT conditions to calculate fold enrichment 2^^-(expCT-ctrlCT)^.

### CUT&RUN library preparation, sequencing, and analysis

Libraries were prepared with the NEBNext Ultra II DNA Library Prep Kit for Illumina (New England Biolabs) with protocol modifications described in [105]. Samples were indexed with NEBNext Multiplex Oligos for Illumina (Dual Index Primers Set 1, E7600). An aliquot of 4–6 ng CUT&RUN DNA was processed for each sample. DNA was end repaired and adaptors were ligated. To clean up the reaction, 1.75× volume of Agencourt AMPure XP beads was added to retain short ligation products. PCR amplification was performed for 15 cycles. The resulting libraries were purified with 1.2× volume of AMPure beads then analyzed and quantified by Tapestation (D1000 screen Tape). Libraries were normalized to 4 nM and pooled. The library pool was denatured and diluted to approximately 12 pM and spiked with 10% PhiX as an internal control. Illumina paired-end sequencing (80x80 cycles) was performed either on the MiSeq with the MiSeq Reagent kit v3 (150 cycles) or on the Nextseq 500 platform with NextSeq 500/550 High Output Kit v2 (150 cycles). Paired-end reads were aligned to the EBV Akata reference genome KC_207813 using Bowtie 2 (version 2.2.4). Mapped reads are shown in the S4 Table. For identification of LEF1 binding, DNA fragments between 50 and 300 bp in length were analyzed as per https://github.com/peteskene. For peak calling, Bedtools (2.21.0) was used. Thresholds were set at 40 except for IgG control and LEF1 in experiment 3 (Fig 1), which was set at 150. Cut-and-Run sequencing data is available at Gene expression Omnibus (Accession number GSE245534).

### siRNA knockdown

NOKs were seeded at 1.5 × 10^5^ cells/well in 12 well plates. Twenty-four hours post-seeding, cells were transfected with 20 nM siRNA and 6.3 μL/mL Dharmafect 1 (Dharmacon/Horizon) in a total volume of 500 μL KSFM for 6 hours. Forty-eight hours post siRNA transfection, cells were harvested or treated with differentiation media (KSFM, 10% FBS, 1.2 mM CaCl_2_) for an additional 48 hours. siLEF1 FL (siGENOME, D-015396-01) targeted exon 1a depleting only full-length LEF1 isoforms. siLEF1FL+ΔN (SiGENOME, D-015396-04) targeted exon 2 depleting all LEF1 isoforms. Non-targeting siRNA control (siGENOME, D-001210-05) was used. Akata BX1 cells were transfected via electroporation with either siLEF1 or NT siRNA using the Neon transfection system (Life Technologies), according to the manufacturer’s instructions. Briefly, cells were harvested, washed with PBS, and resuspended in Neon buffer R at a density of 1 x 10^7^ cells/mL. Neon 100 μL electroporation tips were used for each reaction with electrolytic buffer 2. siRNA was added to the buffer R/cell suspension for a final concentration of 20 nM (1 x 10^6^ BX1 per 100 μL R buffer at siRNA 20 nM). Electroporation was performed with the following settings: 1375 V and 3 pulses lasting 10 milliseconds. Electroporated cells were placed into 2 mL complete media (RPMI 10% FBS) and incubated at 37°C with a 5% CO_2_ atmosphere. Cells were harvested for CUT&RUN or immunoblotting analysis 24 hours post electroporation.

### Immunofluorescence microscopy

Cells (1 × 10^5^ cells/well)l were seeded onto microscope coverslips for 24–96 hours based on experimental design. Cells were fixed for 20 minutes with ice cold 4% PFA, permeabilized with 0.1% Triton X-100 for 10 minutes, and blocked at room temperature in 5% goat serum/PBS for 30 minutes. Primary antibody (1:100) was incubated overnight in 2.5% goat serum/PBS blocking buffer at 4°C. Slides were thoroughly washed in PBS and a secondary antibody was added (1:1000 anti-rabbit Alexa Fluor 647 (Thermo) and 1:1000 anti-mouse Alexa Fluor 546 (Thermo Fisher) for 1 hour at room temperature. Coverslips were mounted using Prolong Glass Antifade (Thermo Fisher) and cured overnight at room temperature. Immunofluorescence microscopy was performed using the Zeiss AxioObserver Z1 inverted fluorescent microscope with Apotome and Zen software. Six random images were taken using a 20X objective and were quantified manually using via ImageJ/Fiji software analysis. Raw Microscope image files were deposited in Dryad [106].

### Dryad DOI

https://doi.org/10.5061/dryad.9zw3r22n8

### Reverse transcription quantitative PCR (RT-qPCR)

Total cellular RNA was isolated via STAT-60 homogenization and chloroform/isopropanol isolation. RNA (1 μg) was used to generate cDNA using LunaScript-RT Master Mix (NEB) as directed by the manufacturer. Reaction time was increased to 1 hour for detection of ΔN LEF1 expression. RT-qPCR was performed on a 7500 FAST Applied Biosystems thermocycler using Luna Universal qPCR master mix (NEB), 50 ng cDNA, and 300 nM primers in each 15 μL reaction. Thermocycling parameters were an initial denaturation at 95°C for 10 min followed by 40 cycles of denaturation at 95°C for 15 seconds and annealing/extension at 60°C for 1 minute. Relative RNA levels were determined using standard curve analysis based on serially diluted cDNA derived from clonal EBV-positive (E+cl) NOK cells, Akata BX1 cells, or the clonal uninfected cell line as required. The cellular housekeeping gene Peptidylprolyl isomerase A/cyclophillin A (PPIA/CypA) was used as a normalization control. Negative controls included reverse transcriptase-negative reactions and water as template. Primers are listed in S3 Table.

### LEF1 overexpression analysis

pCMV6-A-puro (ORIGENE PS100025) vector was used for expression of LEF1 variants. LEF1 variant 3 (NM_001130714) and variant 4 (NM_001166119) encoding DNA fragments were shuttled from pCMV6-entry vector-neo (PS100001) by digestion with BamH I and Pme I restriction enzymes and introduced at the same restriction sites in pCMV6-A-puro. 5 × 10^5^ EBV+NOK cells were seeded at in 6 well plates. The next day cells were transfected with 2 μg DNA using Lipofectamine 3000 reagents (2 μL Lipofectamine 3000/μg DNA, Thermo). Cells were placed under selection for 72 hours in 1 μg/mL puromycin. Puromycin was removed and G418 sulfate/neomycin (50 μg/mL) was added to aid in maintenance of the EBV genome. Stably transfected cells were seeded onto coverslips and differentiated as described above. For transient LEF1 expression,5 × 10^5^ cells/well were seeded onto microscope coverslips (size # 1 circular) in a 12-well plate 24 hrs prior to DNA transfection. Each well was incubated with Fugene transfection reagent (3:1 reagent/DNA) and 1.5 μg, pCMV6 LEF1 variant 1 or pCMV6 LEF1 variant 4 plasmid DNA for 6 hours. Cells were allowed to recover for 24 hours and then incubated for 48 hours in KSFM 10%FBS 1.2 μM CaCl_2_ to promote differentiation. Immunofluorescence staining was performed as previously described to analyze expression of BZLF1 and myc-tagged LEF1 isoforms.

### Western blot

Protein lysates were collected in 100 μL RIPA buffer (150 mM NaCl, 150 mM Tris pH 8.0, 0.5% deoxycholate, 1.0% NP40, 0.1% SDS) and combined with an equal volume of 2X SDS loading buffer. Samples were boiled for 3 minutes at 95°C and quenched on ice. Lysate (25 μ) was loaded in 12% Tris-tricine gels and run at constant voltage (90V/3.5hrs). Gels were then transferred overnight at 30 volts onto a 0.22-micron nitrocellulose membrane (Millipore) in 15% methanol transfer buffer. Histone proteins and histone acetylation immunoblots were performed using 15% Tris-glycine gels, 200V/30min electrophoresis and 90V/70min transfer times. Fluorescent western blotting was used for detection. Membranes were blocked with Odyssey Blocking Buffer (LI-COR) at room temperature for 1 hour before incubating with the indicated primary antibodies overnight at 4°C. Following 4 TBST washes, Odyssey secondary antibodies (goat anti-rabbit IRDye 800CW and/or goat anti-mouse IRDye 680RD; dilution 1:15000) were applied for 1 hour at room temperature. After 4 Tris-buffered Saline 0.1% Tween-20 (TBST) washes, blots were imaged using an Odyssey DLx Infrared Imaging System (LI-COR). Scan resolution of the instrument ranges from 21 to 337 μm, and in this study blots were imaged at 169 μm. Quantification of fluorescent signals was performed on single channels using Image Studio Lite software (LI-COR) according to the manufacturer’s instructions. Primary antibodies used are listed in the S2 Table.

### Statistical analysis

Data were expressed as means ± standard error (SE) of at least three independent experiments (n ≥ 3), and the statistical significance was calculated using a two-tailed Student’s t test or Mann-Whitney U test as indicated. Data were expressed as means ± standard deviation (SD) from experiments with only 2 replicates. Values were considered significant if the P value was <0.05.

## Supporting information

S1 Fig Wnt response elements are conserved on type 1 and type 2 EBV genomes. **(A)** Mapping of WRE motif (5’-CTTTGWWS-3’) on an EBV type 1 reference genome (NC_007605). Top track depicts the EBV genome. Blue lines are motifs on the plus DNA strand, while red lines show motifs on the minus strand. * indicates motifs only present on the type 1 EBV genome. ^@^ indicates motif not conserved on the Akata EBV genome. ^#^ indicates 2 adjacent motifs at positions (36909 and 37005) (**B)** Mapping of WRE motif (5’-CTTTGWWS-3’) on an EBV type 2 reference genome (NC_009334). Top track depicts the EBV genome. Blue lines are motifs on the plus DNA strand, while red lines show motifs on the minus strand.
(TIF)

S2 Fig LEF1 depletion does not induce spontaneous reactivation in latently infected EBV+NOK. **(A)** EBV+NOK were transfected with siRNAs to specifically deplete the full-length LEF1 isoforms (siLEF1 FL) and all LEF1 isoforms (siLEF1 FL+DN). Shown is the immunofluorescence analysis performed for LEF1 and BZLF1 at 96 hours post transfection. **(B)** BZLF1-positive cells were quantified at 48- and 96-hours post-siRNA transfection. The mean and standard deviation from the mean is shown for two independent experiments.
(TIF)

S3 Fig Transient expression of ΔN LEF1 reduced EBV reactivation compared to the full-length LEF1 isoform 1. **(A)** Ectopic expression of LEF1 in EBV+NOK following transient transfection of Myc-tagged LEF1 isoforms (LEF1 FL isoform 1 and **ΔN** LEF1. Cells were treated with calcium and serum for 48 hours to induce differentiation. Immunofluorescence co-staining detected BZLF1 (red), LEF1-myc epitope (green) and merge/Hoeschst (blue). White arrows point to LEF1(myc-tag)/BZLF1 double positive cells **(B)** Transfection efficiency measuring number cells expressing exogenous LEF1 (myc-tag). The mean and standard deviation from the mean is shown for two independent experiments. **(C)** Quantitation of the number of double positive cells expressing Z and LEF1 (myc epitope tag). Six random images for each transfection condition were counted. Shown are the mean and standard deviation for two independent experiments.
(TIF)

S4 Fig LEF1 expression in EBV+ Burkitt’s lymphoma cell lines. RT-qPCR analysis using primers to detect LEF1 transcripts in Akata BX1, Glor, Kem-I, Mutu-I, P3HR1 cl16, Salina, and Tiazaru. Shown is the LEF1 transcript level relative to cyclophillin (PPIA) (n = 1).
(TIF)

S5 Fig Overexpression of ΔN LEF1 does alter host responsiveness to differentiation. EBV+NOK overexpressing LEF1 variant 4 (ΔN) were induced with calcium and serum for 48 hours. Cells transfects with pCVM6 served as the vector control, and ΔN refers to cells transfected with pCMV6-LEF1ΔN. Immunofluorescence analysis was performed for (**A, E**) PRDM1/BLIMP1; (**B, F**) KLF4; (**C**) involucrin. Shown are images used for quantitation. (**D**) Percentage of positive cells for KLF4, PRDM1/BLIMP1, and involucrin (n = 1) in EBV+NOK overexpressing LEF1 variant 4. The mean and standard deviation is shown.
(TIF)

S6 Fig Genome-wide hyperacetylation following ΔN LEF1 knockdown. CUT&RUN-seq bedgraph profiles for **(A)** H3K9ac and **(B)** H3K27ac following transfection of EBV+NOK with siNT (dark blue), siLEF1 FL (light blue or siLEF1 FL+ΔN (pink). The predicted WRE (CTTTGWWS) motif is shown on the plus DNA strand (blue) and minus DNA strand (red). The gene annotation for the EBV Akata genome (KC 207813) is shown on the bottom track.
(TIF)

S1 Table Position of LEF1 peaks detected on the EBV genome. (DOCX)

S2 Table Primary antibodies used in this study. (DOCX)

S3 Table Primers used in this study. (DOCX)

S4 Table EBV mapped reads from CUT&RUN sequencing. (DOCX)

S1 Data Excel spreadsheet with data and statistical analysis shown in the figures. Each worksheet is labeled according to the respective figure panel.
(XLSX)

S2 Data Supporting raw image files for immunoblots used for quantitation. The files are arranged in folders labeled according to the respective figure.
(ZIP)
