# Supplementary figures and images for: A distinct isoform of lymphoid enhancer binding factor 1 (LEF1) epigenetically restricts EBV reactivation to maintain viral latency
Source: PLoS Pathog. 2023 Dec 19;19(12):e1011873. doi: 10.1371/journal.ppat.1011873 (PMC10763950; doi:10.1371/journal.ppat.1011873)

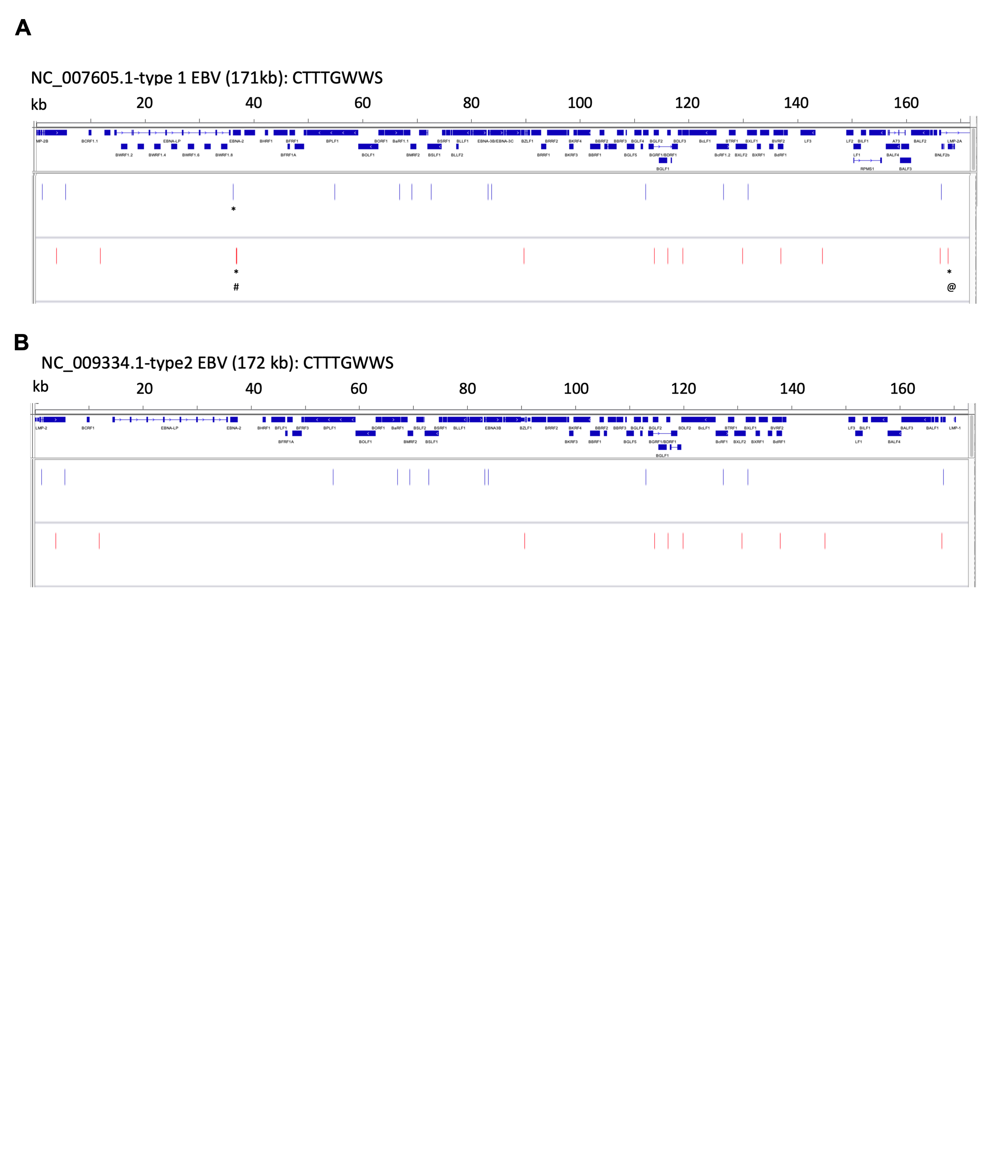

Supplement: S1 Fig — (A) Mapping of WRE motif (5’-CTTTGWWS-3’) on an EBV type 1 reference genome (NC_007605). Top track depicts the EBV genome. Blue lines are motifs on the plus DNA strand, while red lines show motifs on the minus strand. * indicates motifs only present on the type 1 EBV genome. @ indicates motif not conserved on the Akata EBV genome. # indicates 2 adjacent motifs at positions (36909 and 37005) (B) Mapping of WRE motif (5’-CTTTGWWS-3’) on an EBV type 2 reference genome (NC_009334). Top track depicts the EBV genome. Blue lines are motifs on the plus DNA strand, while red lines show motifs on the minus strand. (TIF) [file ppat.1011873.s001.tif]

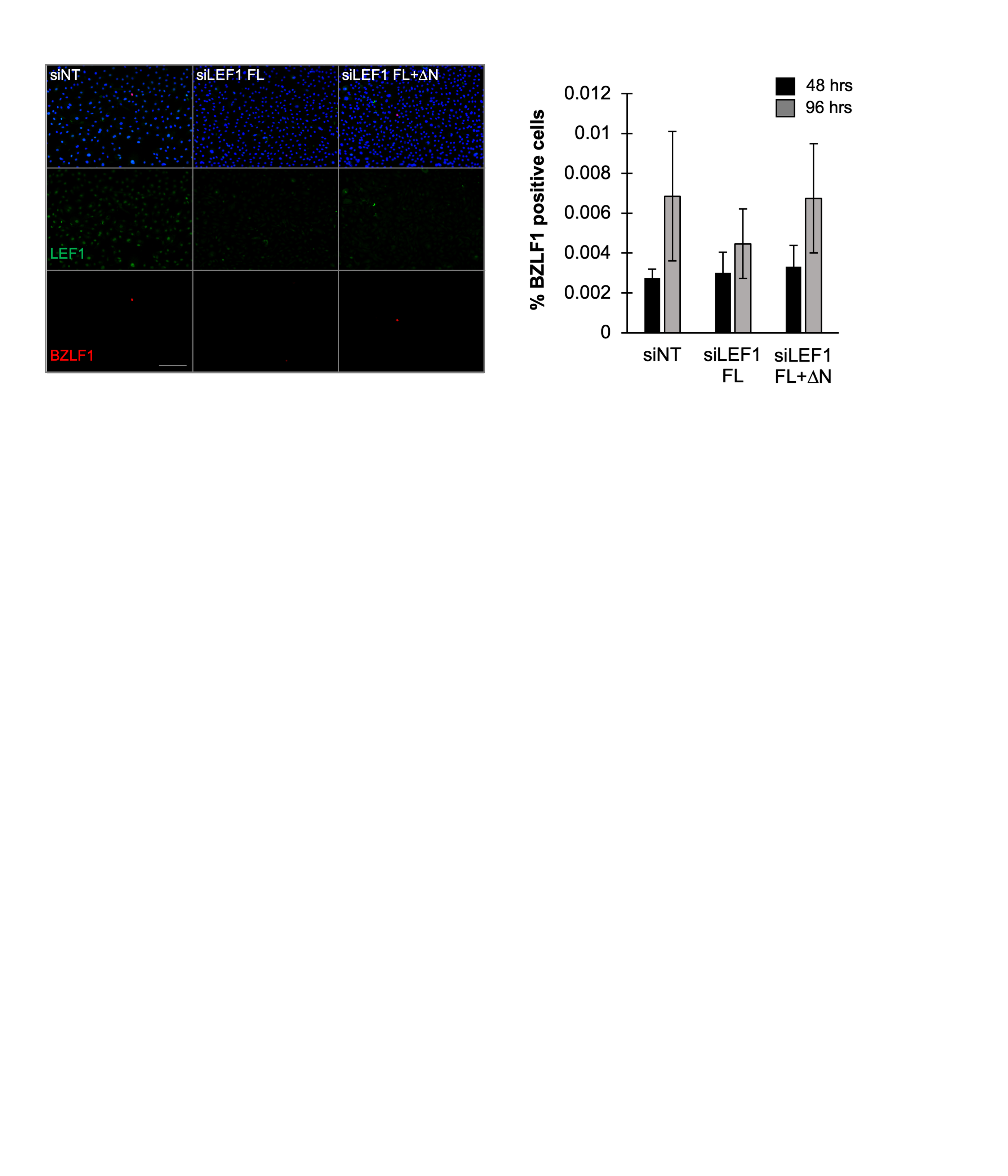

Supplement: S2 Fig — (A) EBV+NOK were transfected with siRNAs to specifically deplete the full-length LEF1 isoforms (siLEF1 FL) and all LEF1 isoforms (siLEF1 FL+DN). Shown is the immunofluorescence analysis performed for LEF1 and BZLF1 at 96 hours post transfection. (B) BZLF1-positive cells were quantified at 48- and 96-hours post-siRNA transfection. The mean and standard deviation from the mean is shown for two independent experiments. (TIF) [file ppat.1011873.s002.tif]

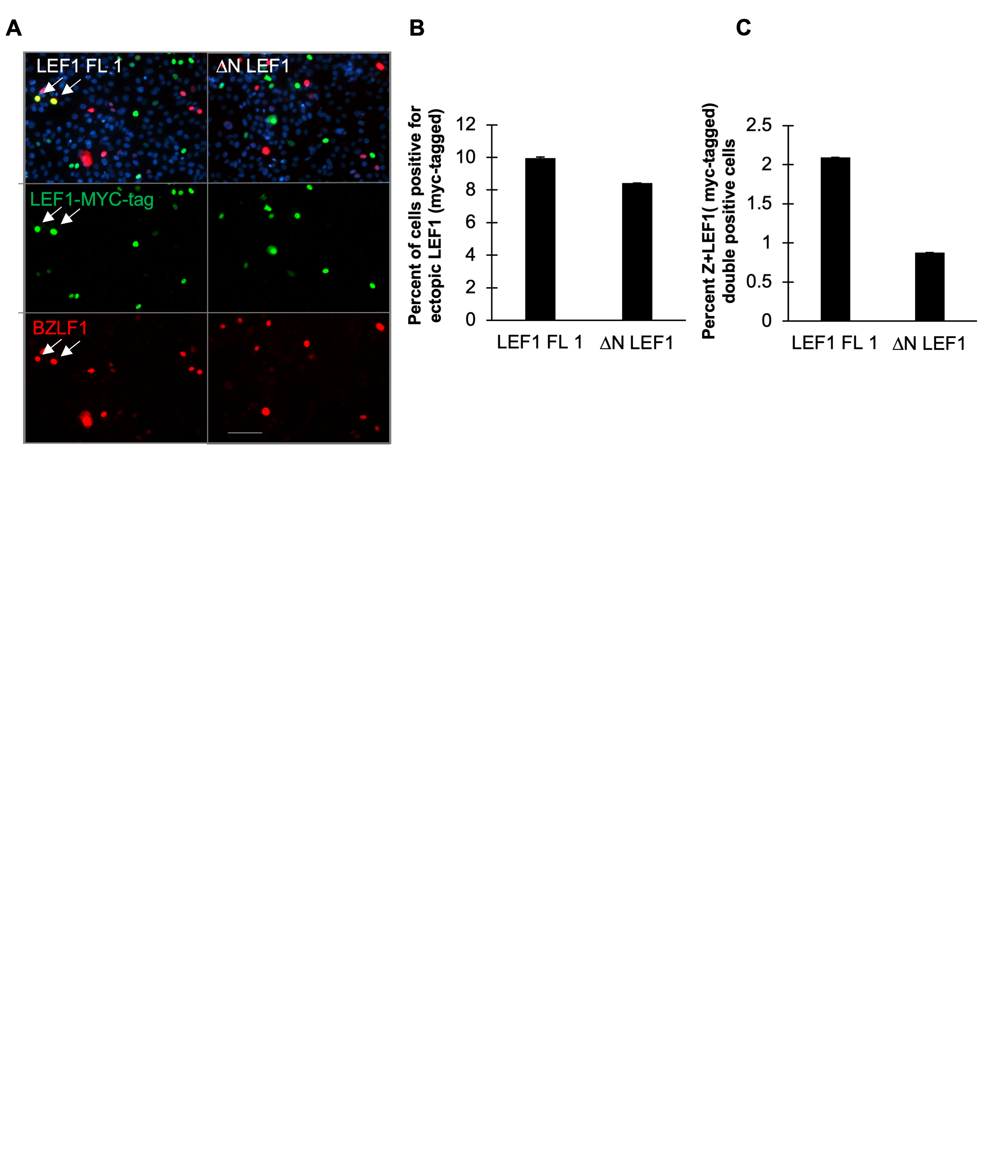

Supplement: S3 Fig — (A) Ectopic expression of LEF1 in EBV+NOK following transient transfection of Myc-tagged LEF1 isoforms (LEF1 FL isoform 1 and ΔN LEF1. Cells were treated with calcium and serum for 48 hours to induce differentiation. Immunofluorescence co-staining detected BZLF1 (red), LEF1-myc epitope (green) and merge/Hoeschst (blue). White arrows point to LEF1(myc-tag)/BZLF1 double positive cells (B) Transfection efficiency measuring number cells expressing exogenous LEF1 (myc-tag). The mean and standard deviation from the mean is shown for two independent experiments. (C) Quantitation of the number of double positive cells expressing Z and LEF1 (myc epitope tag). Six random images for each transfection condition were counted. Shown are the mean and standard deviation for two independent experiments. (TIF) [file ppat.1011873.s003.tif]

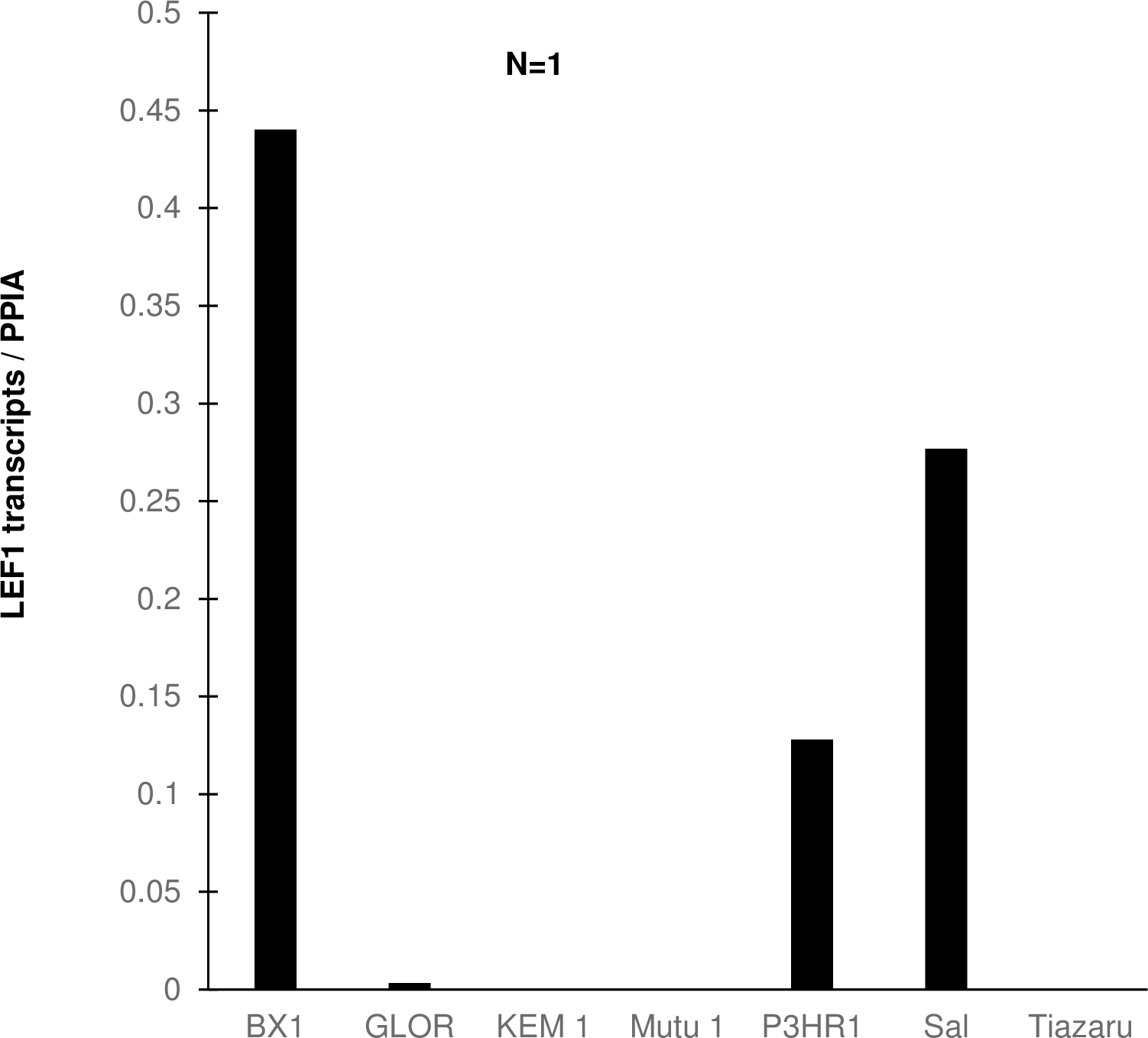

Supplement: S4 Fig — RT-qPCR analysis using primers to detect LEF1 transcripts in Akata BX1, Glor, Kem-I, Mutu-I, P3HR1 cl16, Salina, and Tiazaru. Shown is the LEF1 transcript level relative to cyclophillin (PPIA) (n = 1). (TIF) [file ppat.1011873.s004.tif]

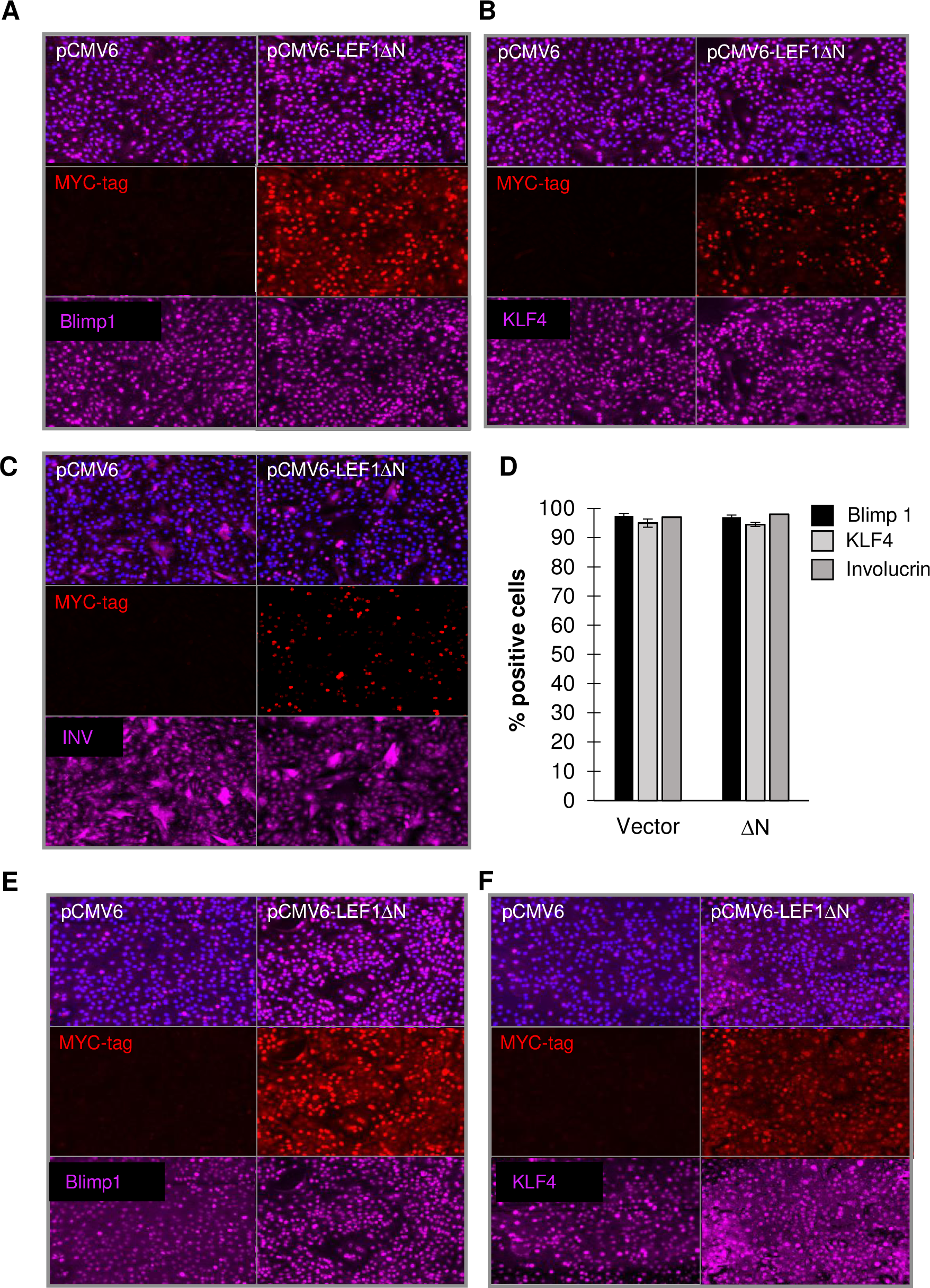

Supplement: S5 Fig — EBV+NOK overexpressing LEF1 variant 4 (ΔN) were induced with calcium and serum for 48 hours. Cells transfects with pCVM6 served as the vector control, and ΔN refers to cells transfected with pCMV6-LEF1ΔN. Immunofluorescence analysis was performed for (A, E) PRDM1/BLIMP1; (B, F) KLF4; (C) involucrin. Shown are images used for quantitation. (D) Percentage of positive cells for KLF4, PRDM1/BLIMP1, and involucrin (n = 1) in EBV+NOK overexpressing LEF1 variant 4. The mean and standard deviation is shown. (TIF) [file ppat.1011873.s005.tif]

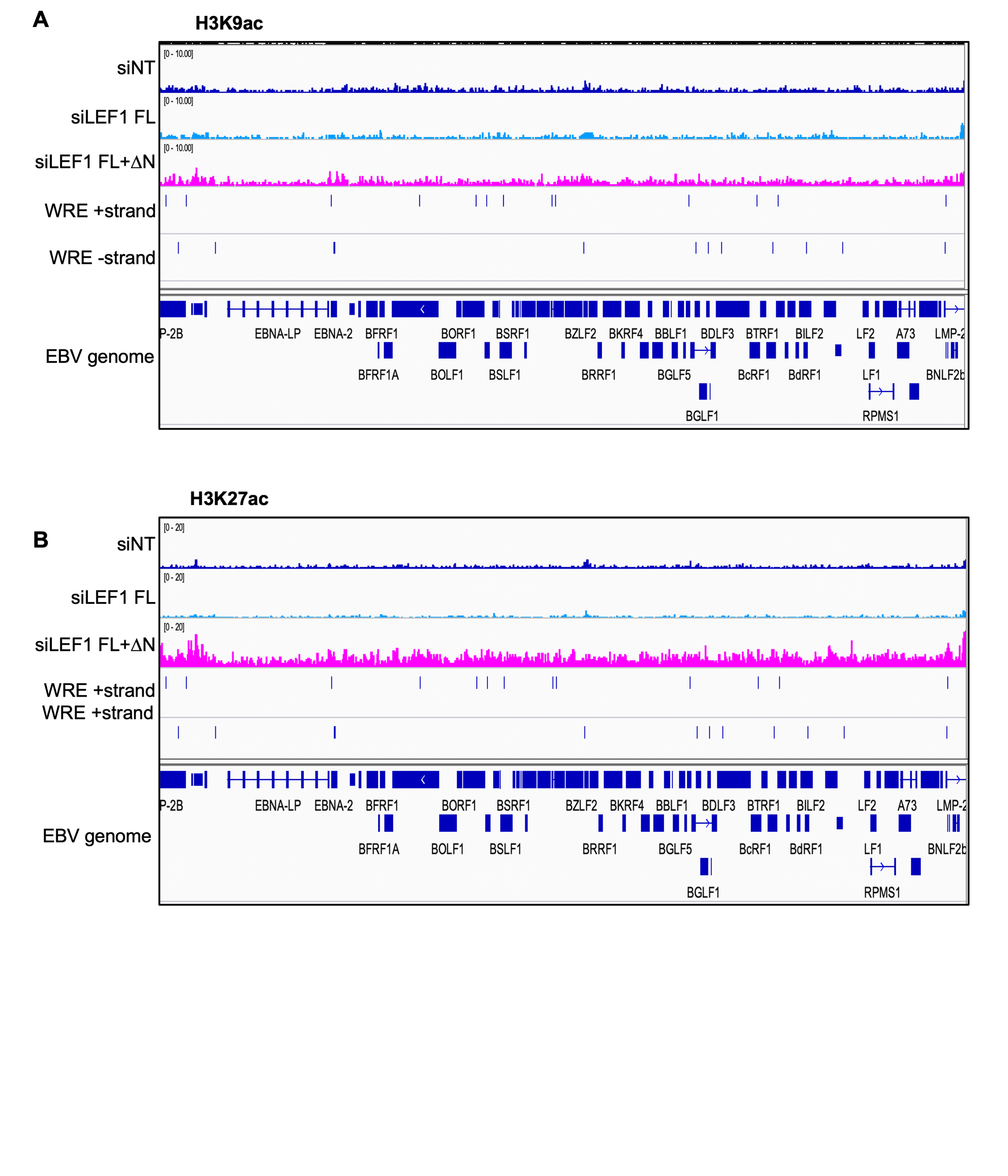

Supplement: S6 Fig — CUT&RUN-seq bedgraph profiles for (A) H3K9ac and (B) H3K27ac following transfection of EBV+NOK with siNT (dark blue), siLEF1 FL (light blue or siLEF1 FL+ΔN (pink). The predicted WRE (CTTTGWWS) motif is shown on the plus DNA strand (blue) and minus DNA strand (red). The gene annotation for the EBV Akata genome (KC 207813) is shown on the bottom track. (TIF) [file ppat.1011873.s006.tif]

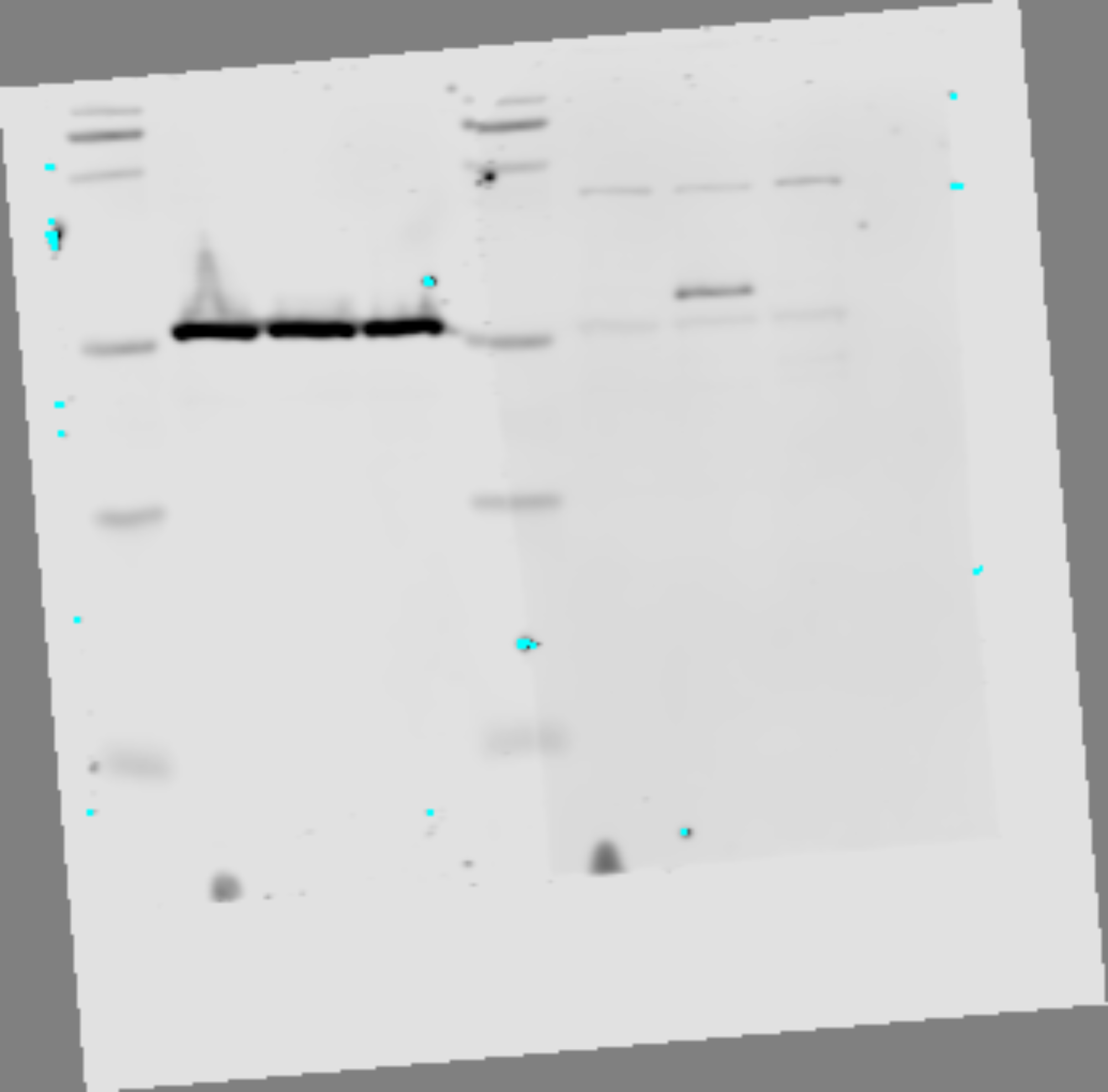

Supplement: S2 Data — The files are arranged in folders labeled according to the respective figure. (ZIP) [file ppat.1011873.s012.zip › WesternBlot Compilation/Figure Blots/Fig 4/Image_Fig4E_tubulin.tif]

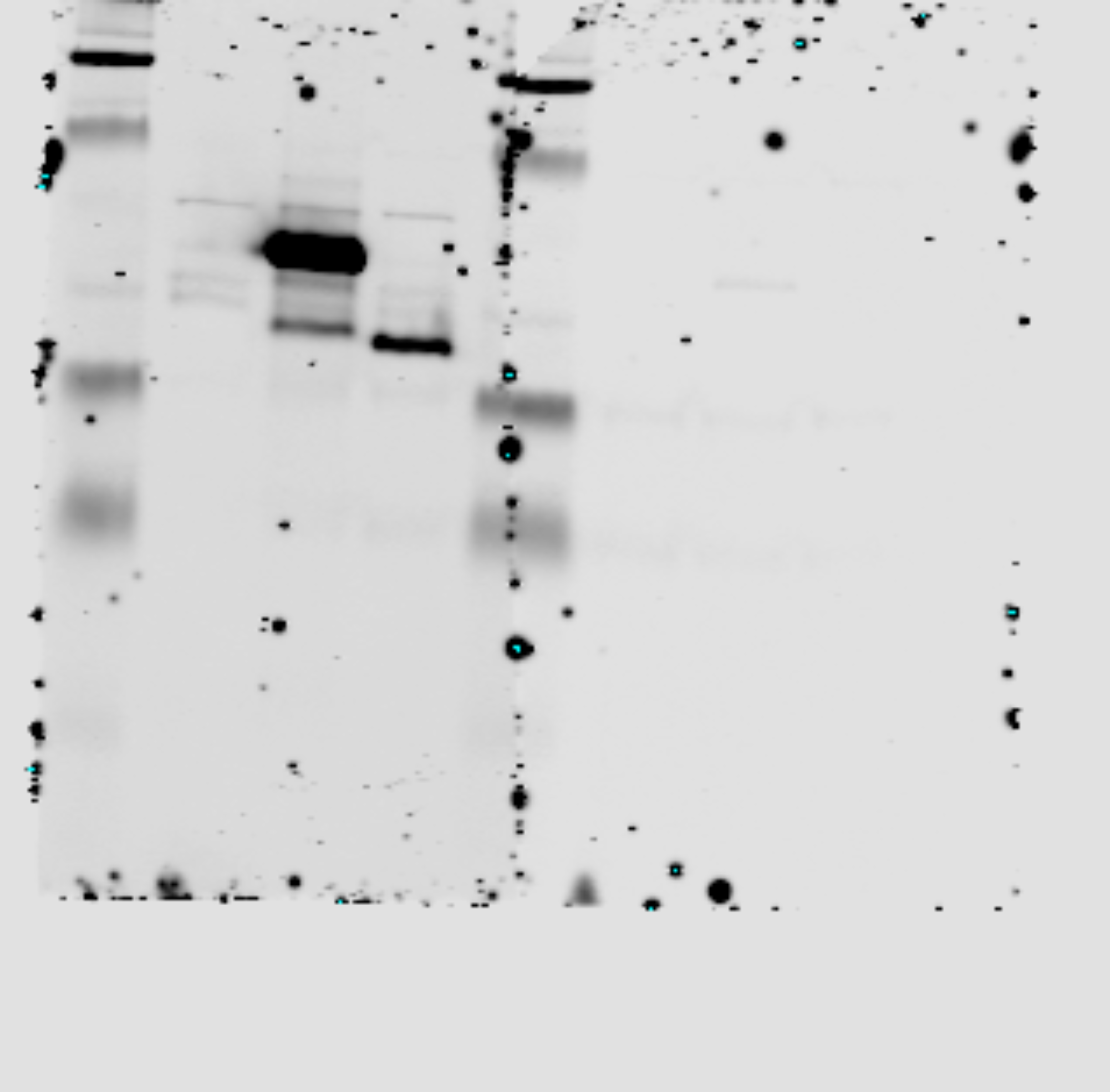

Supplement: S2 Data — The files are arranged in folders labeled according to the respective figure. (ZIP) [file ppat.1011873.s012.zip › WesternBlot Compilation/Figure Blots/Fig 4/Image_Fig4E_LEF1_myc.tif]

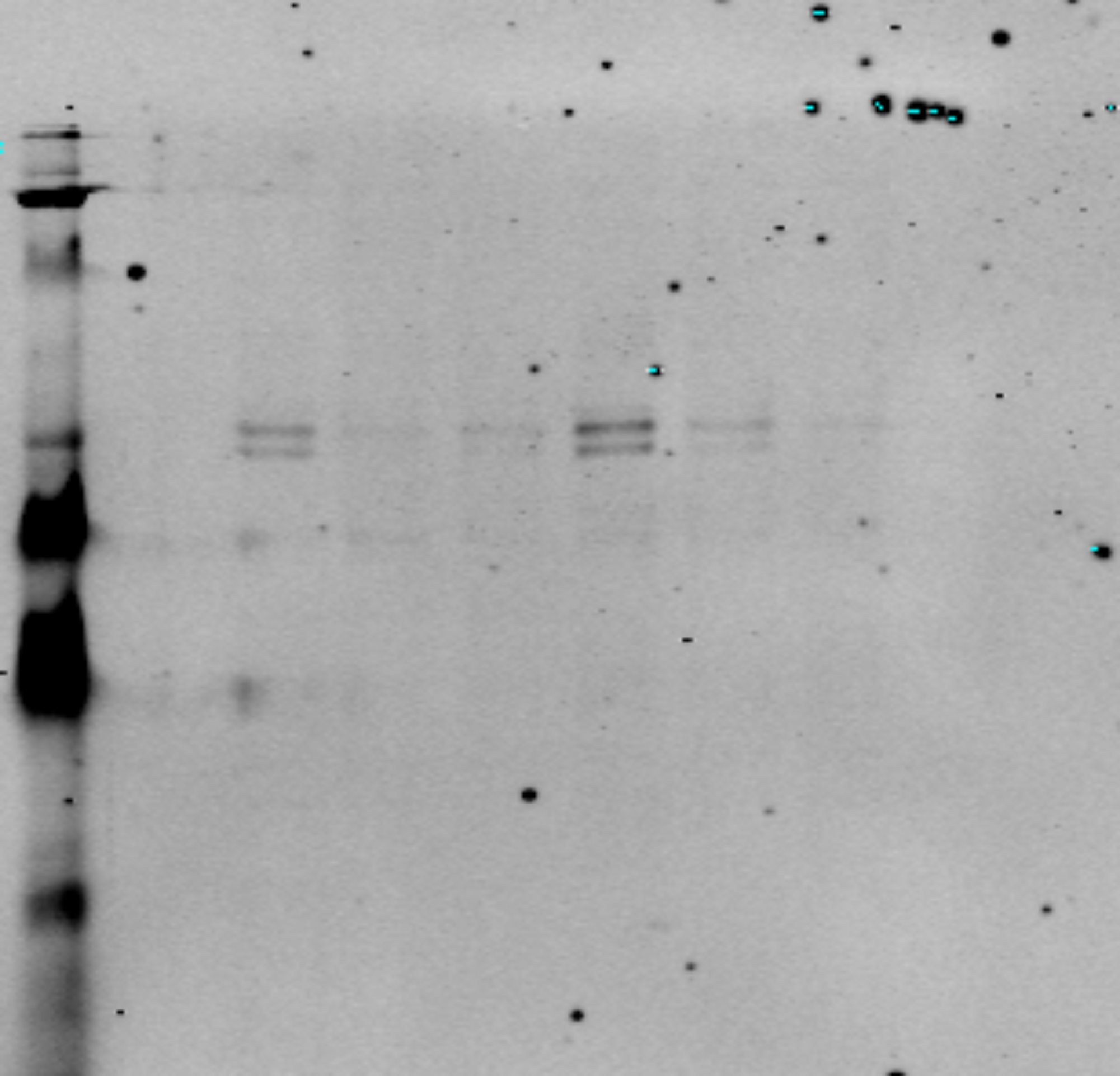

Supplement: S2 Data — The files are arranged in folders labeled according to the respective figure. (ZIP) [file ppat.1011873.s012.zip › WesternBlot Compilation/Figure Blots/Fig 3/Image_Fig3A_LEF1.tif]

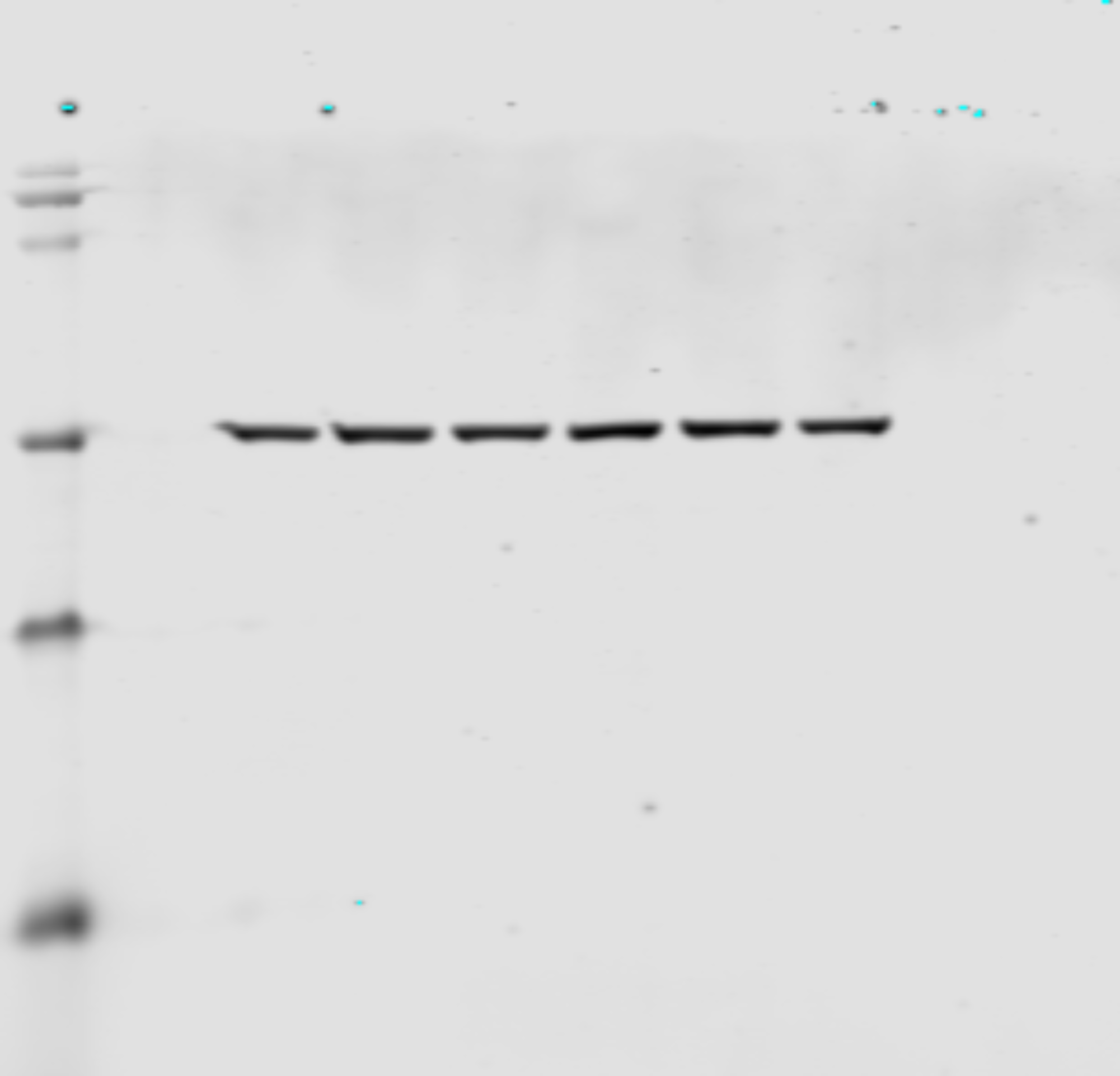

Supplement: S2 Data — The files are arranged in folders labeled according to the respective figure. (ZIP) [file ppat.1011873.s012.zip › WesternBlot Compilation/Figure Blots/Fig 3/Image_FIG3A_tubulin.tif]

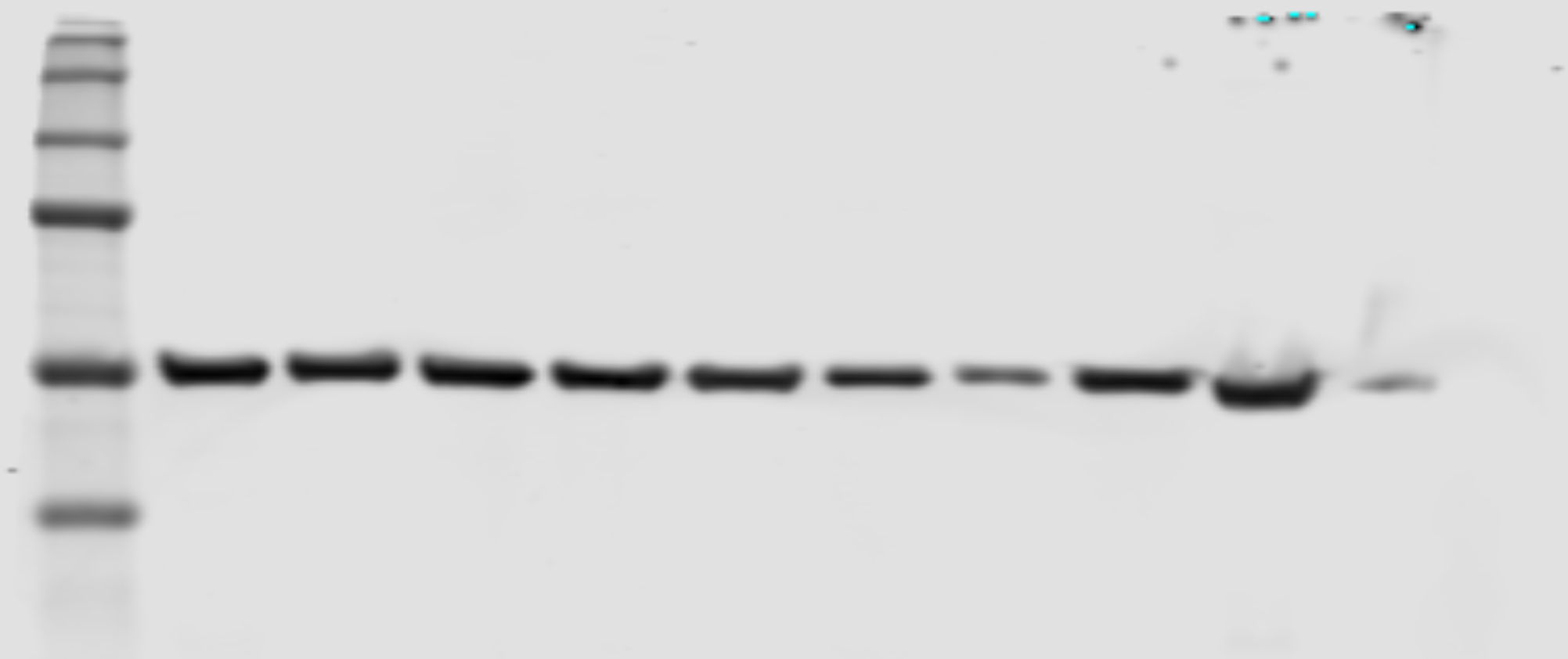

Supplement: S2 Data — The files are arranged in folders labeled according to the respective figure. (ZIP) [file ppat.1011873.s012.zip › WesternBlot Compilation/Figure Blots/Fig 2/_Fig2C_tubulin.tif]

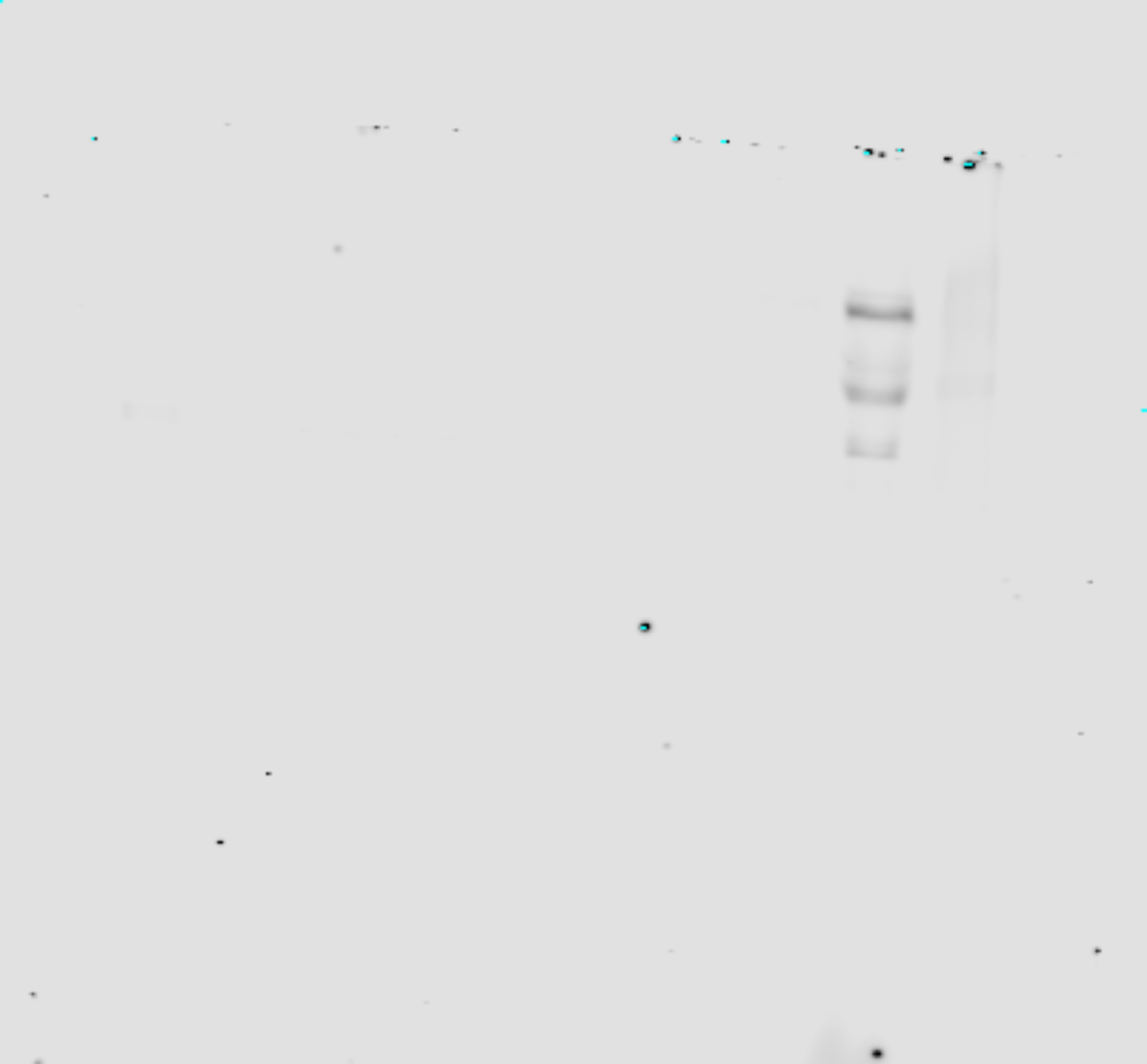

Supplement: S2 Data — The files are arranged in folders labeled according to the respective figure. (ZIP) [file ppat.1011873.s012.zip › WesternBlot Compilation/Figure Blots/Fig 2/_Fig2C_TCF4.tif]

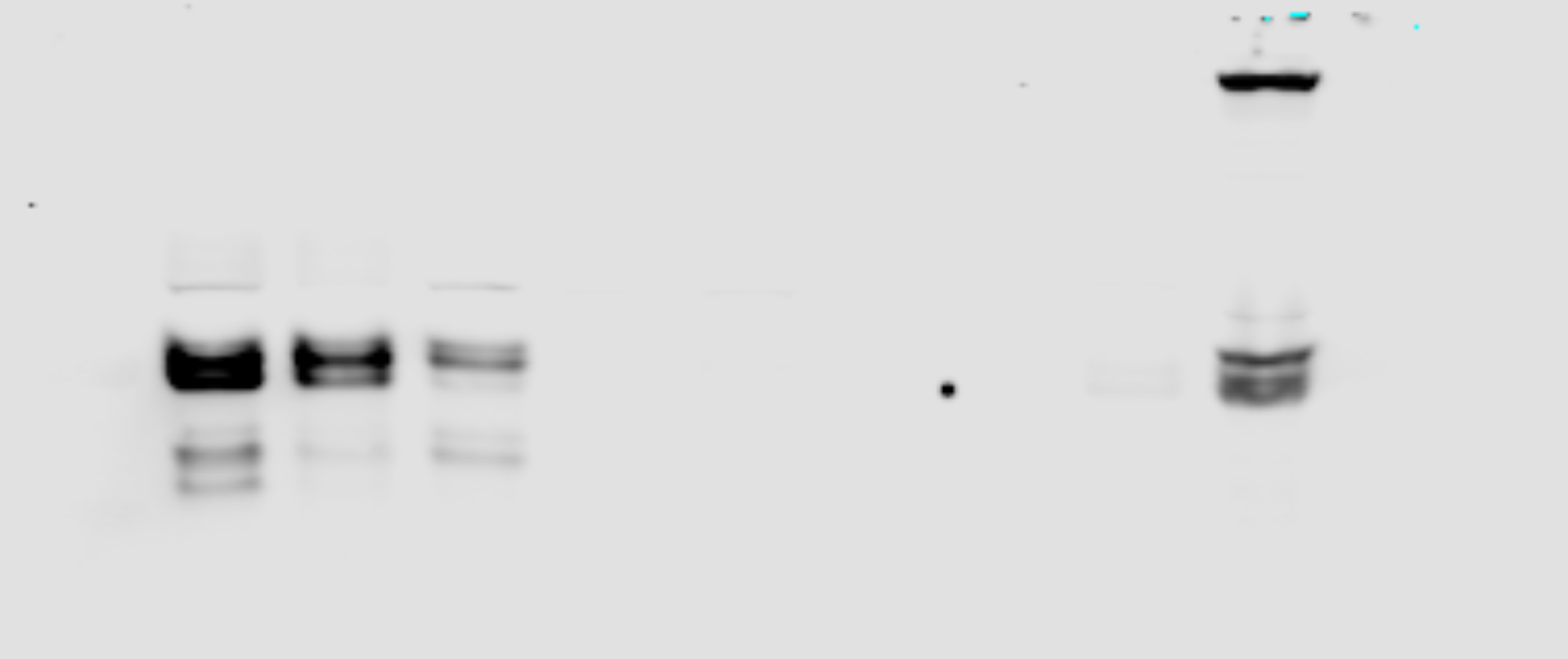

Supplement: S2 Data — The files are arranged in folders labeled according to the respective figure. (ZIP) [file ppat.1011873.s012.zip › WesternBlot Compilation/Figure Blots/Fig 2/_Fig2C_LEF1.tif]

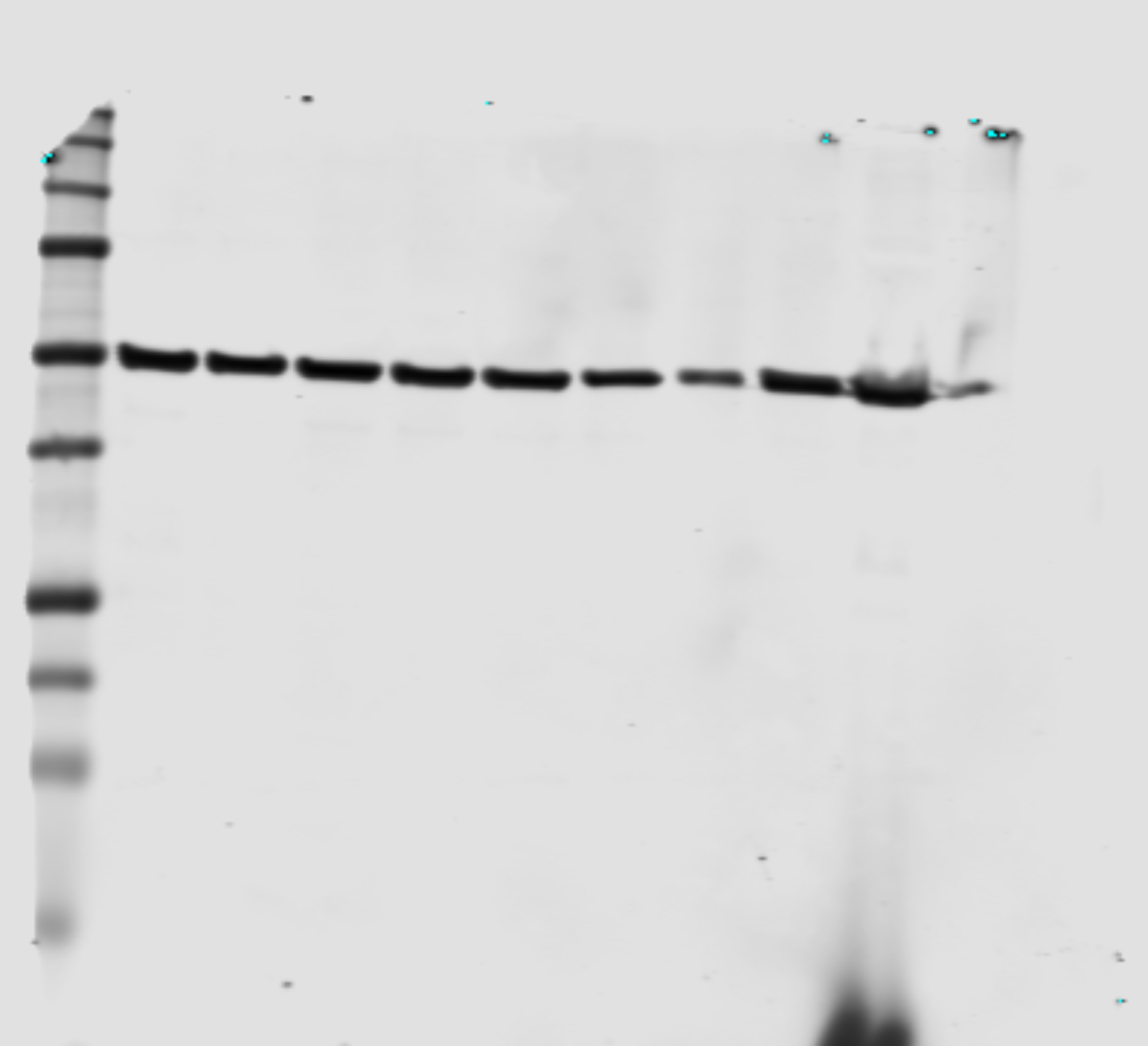

Supplement: S2 Data — The files are arranged in folders labeled according to the respective figure. (ZIP) [file ppat.1011873.s012.zip › WesternBlot Compilation/Figure Blots/Fig 2/_Fig2C_TCF1tubulin.tif]

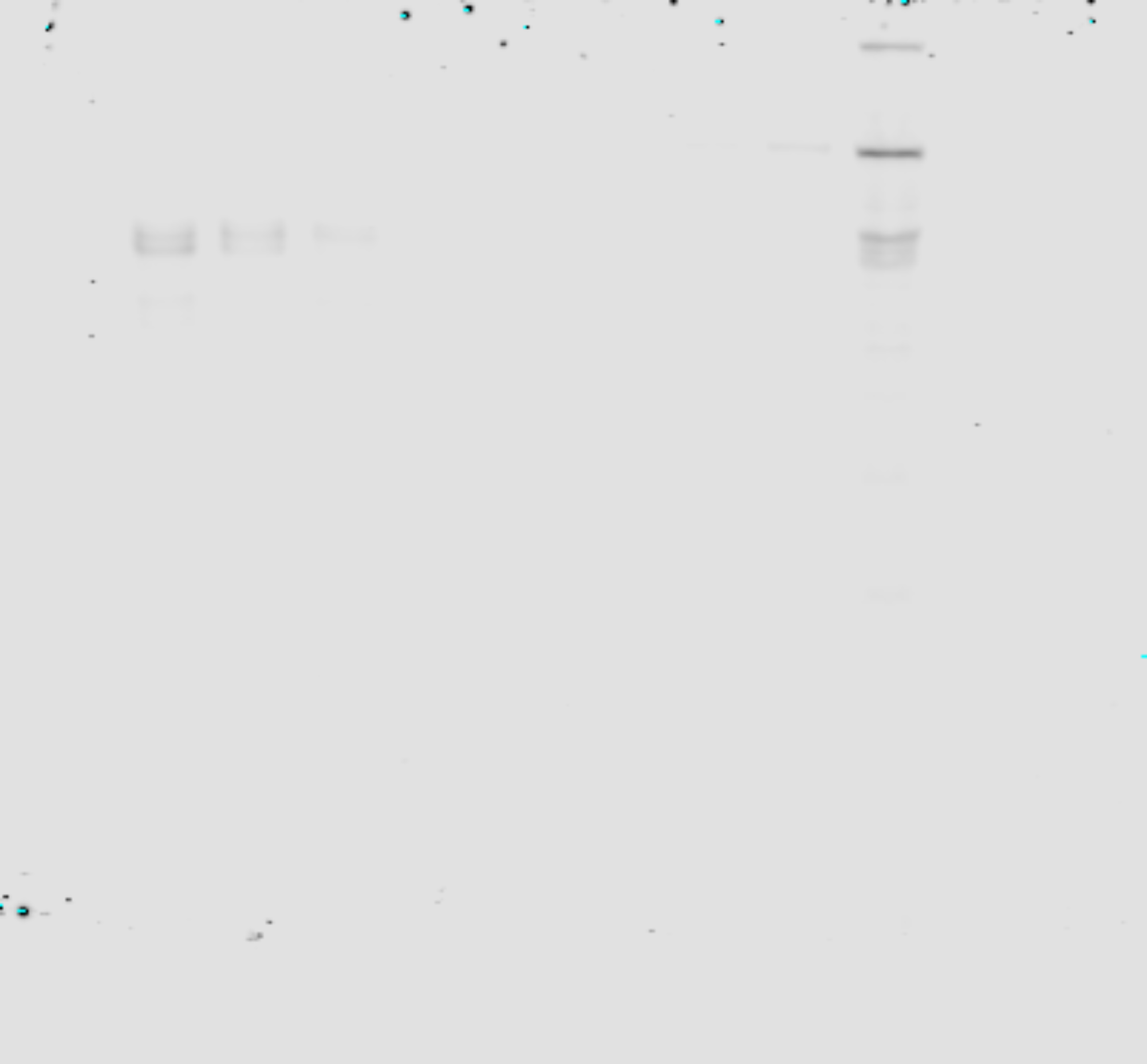

Supplement: S2 Data — The files are arranged in folders labeled according to the respective figure. (ZIP) [file ppat.1011873.s012.zip › WesternBlot Compilation/Figure Blots/Fig 2/_Fig2C_TCF3.tif]

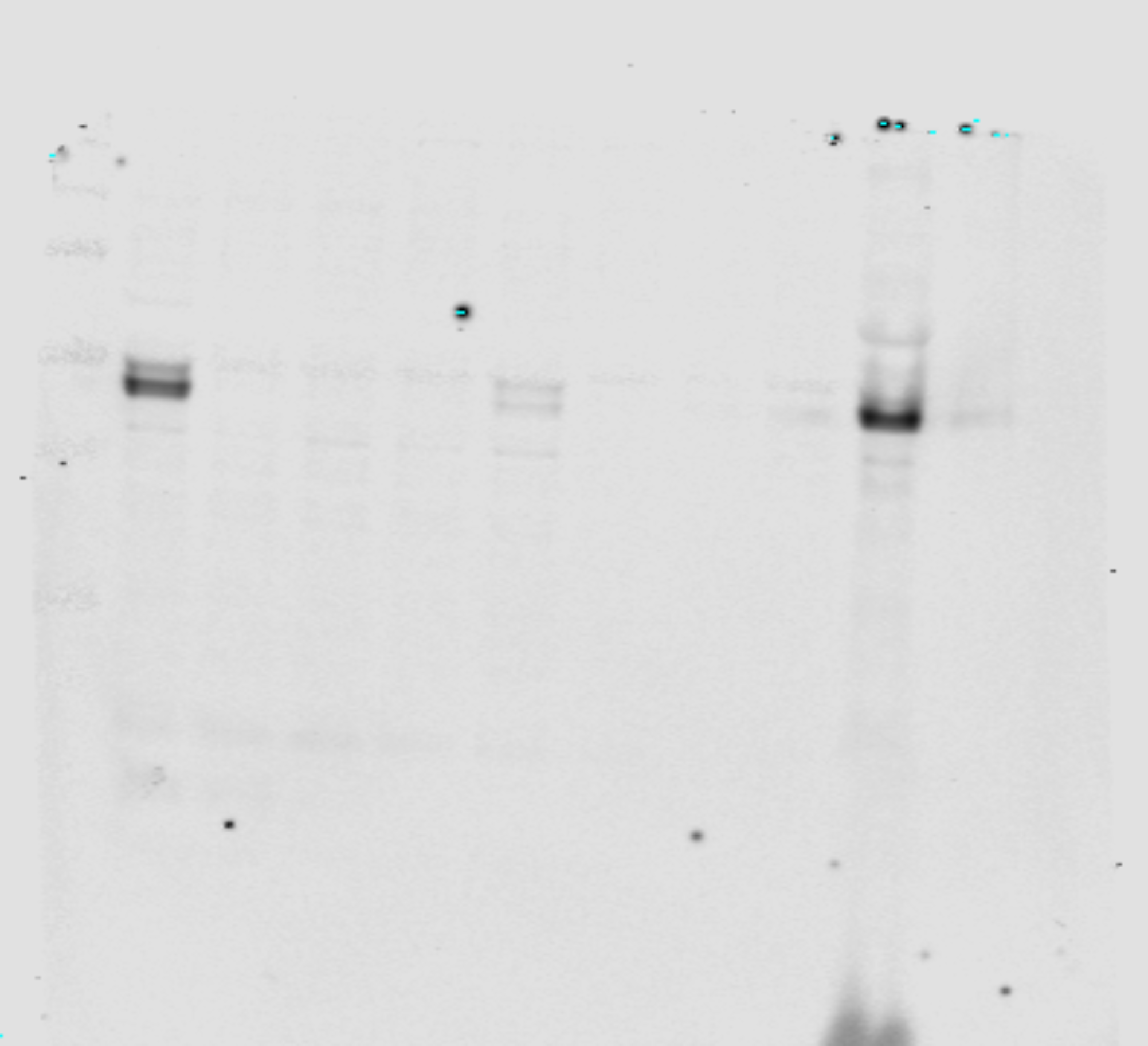

Supplement: S2 Data — The files are arranged in folders labeled according to the respective figure. (ZIP) [file ppat.1011873.s012.zip › WesternBlot Compilation/Figure Blots/Fig 2/_Fig2C_TCF1.tif]

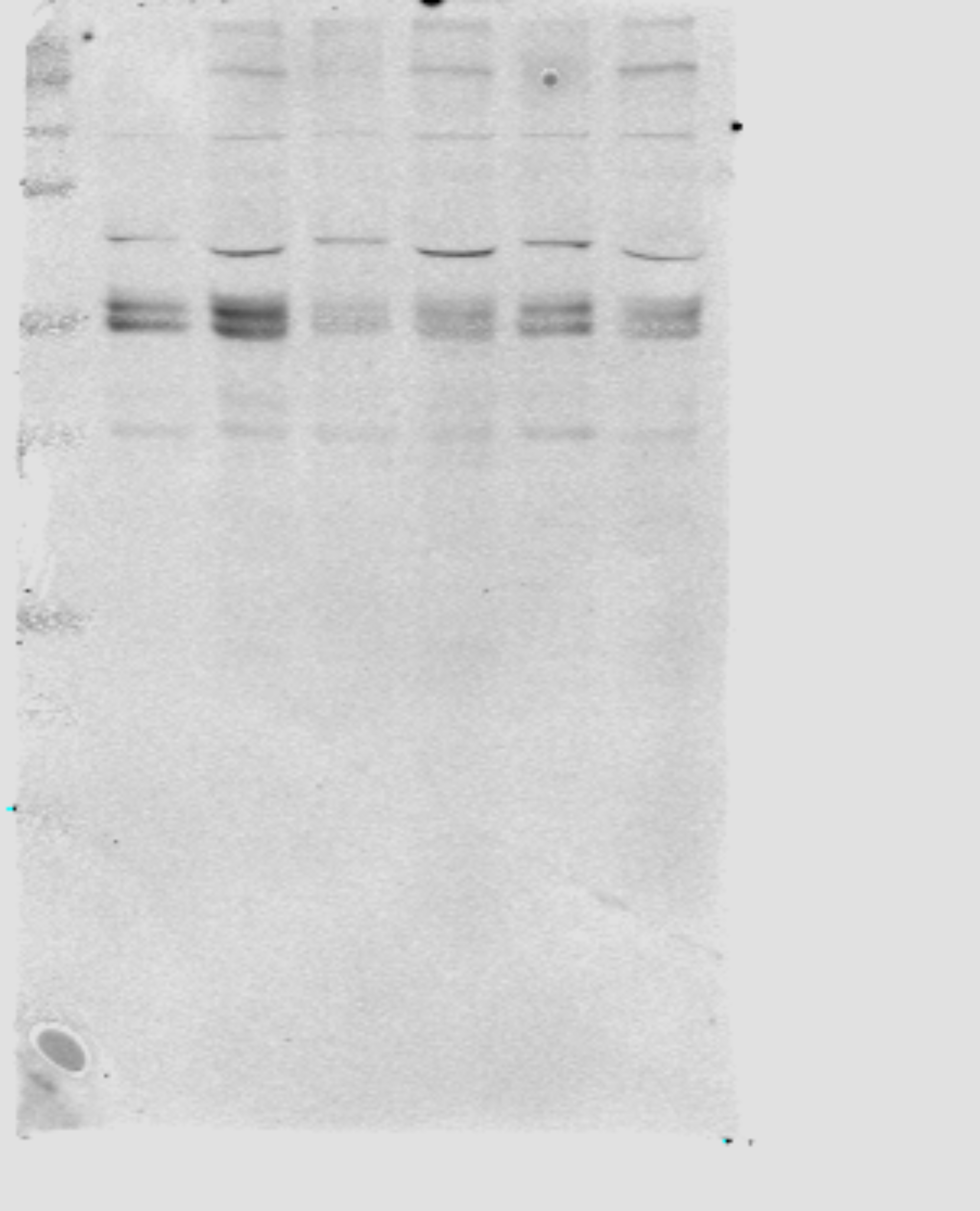

Supplement: S2 Data — The files are arranged in folders labeled according to the respective figure. (ZIP) [file ppat.1011873.s012.zip › WesternBlot Compilation/Figure Blots/Fig 5/Fig5C_LEF1.tif]

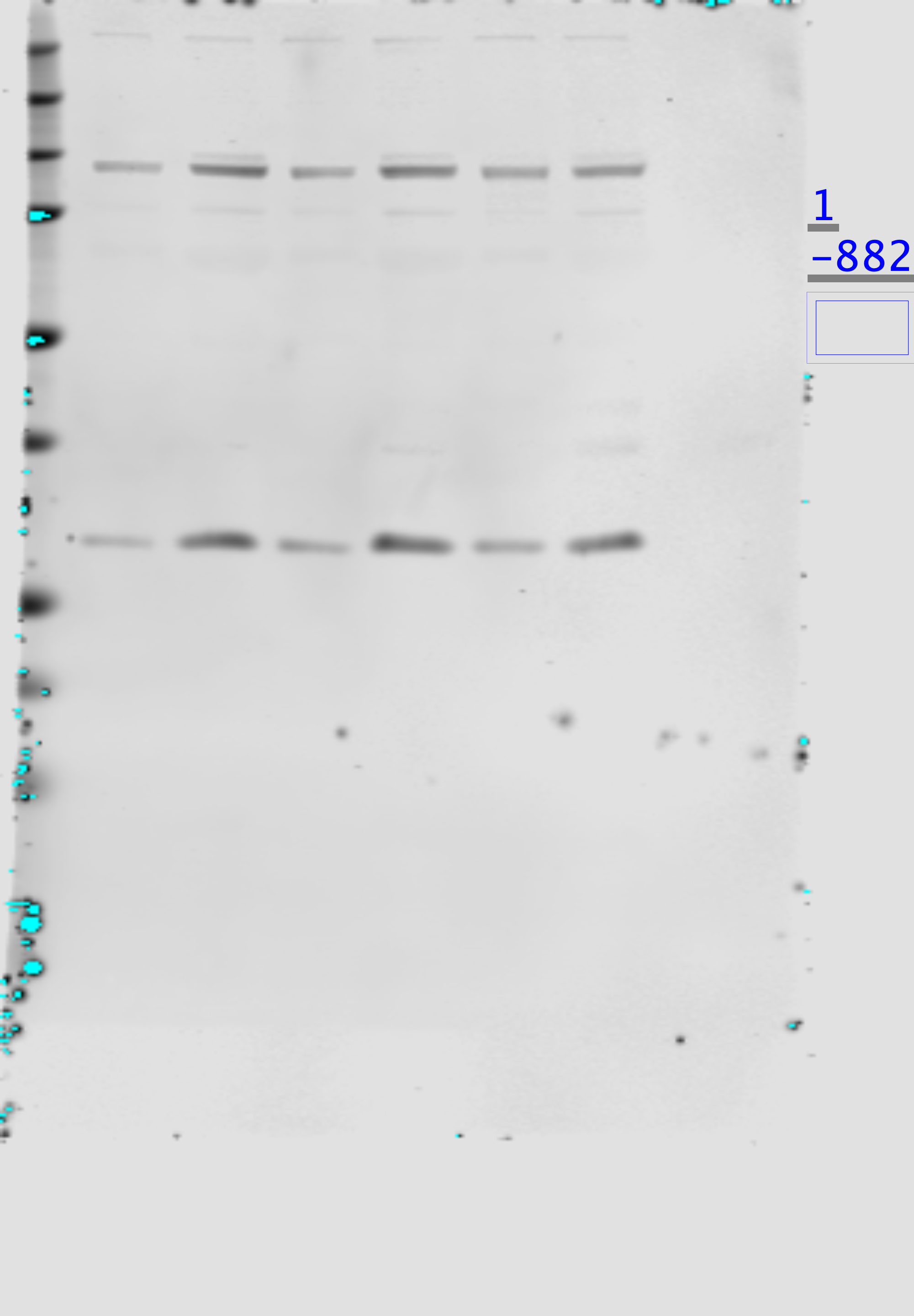

Supplement: S2 Data — The files are arranged in folders labeled according to the respective figure. (ZIP) [file ppat.1011873.s012.zip › WesternBlot Compilation/Figure Blots/Fig 5/Fig5C_Blimp1.tif]

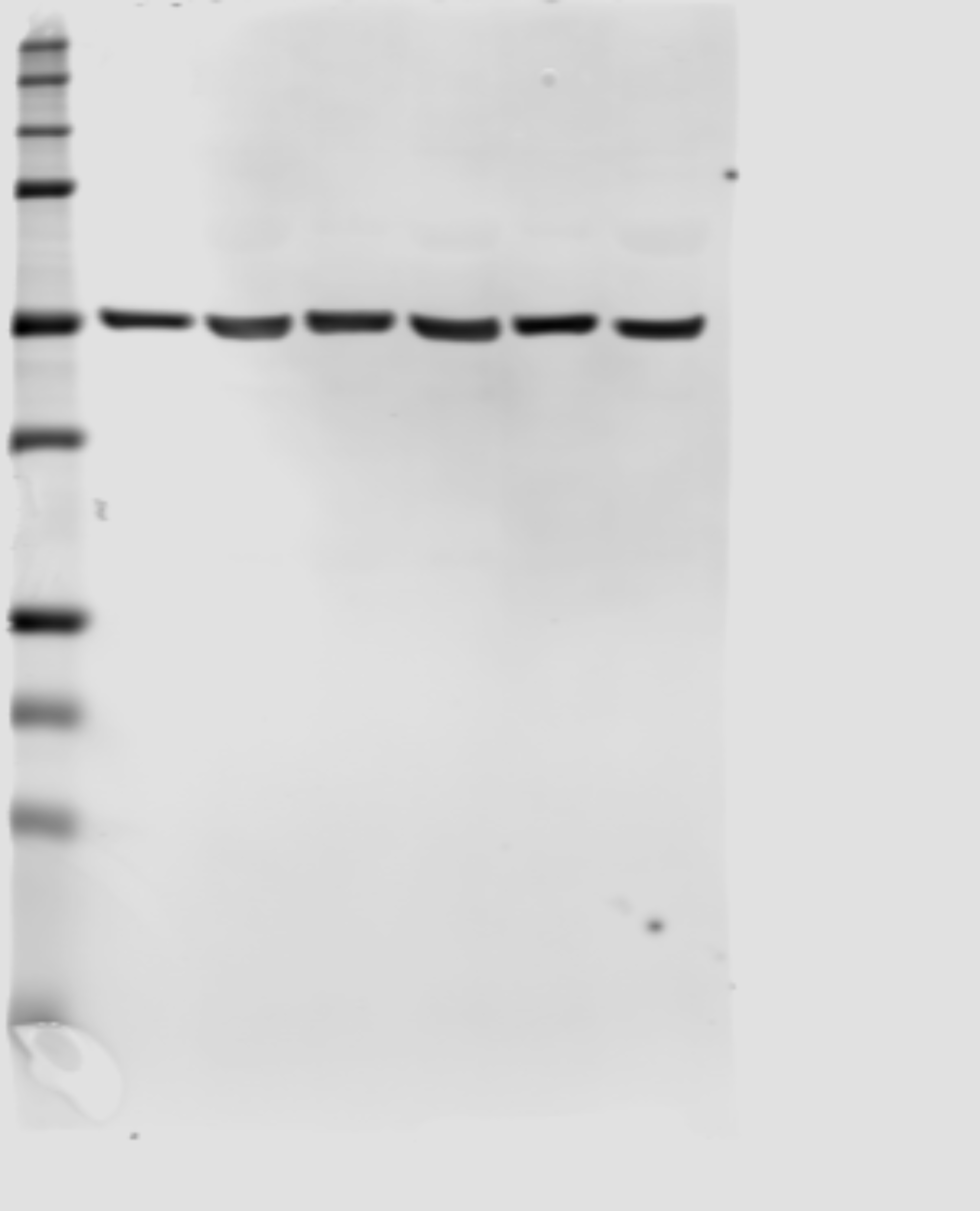

Supplement: S2 Data — The files are arranged in folders labeled according to the respective figure. (ZIP) [file ppat.1011873.s012.zip › WesternBlot Compilation/Figure Blots/Fig 5/Fig5C_LEF1_INVL_loading.tif]

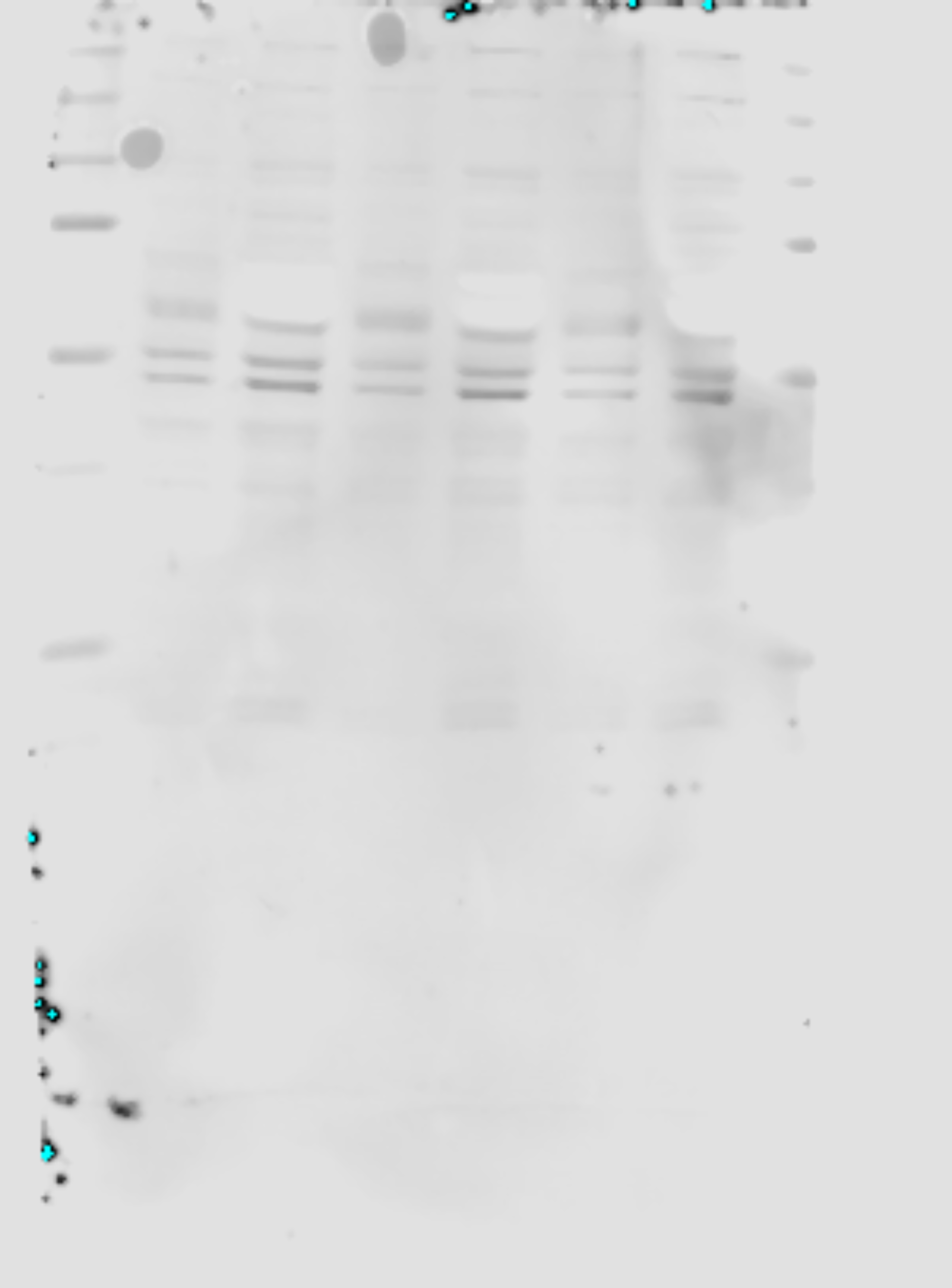

Supplement: S2 Data — The files are arranged in folders labeled according to the respective figure. (ZIP) [file ppat.1011873.s012.zip › WesternBlot Compilation/Figure Blots/Fig 5/Fig5C_KLF4.tif]

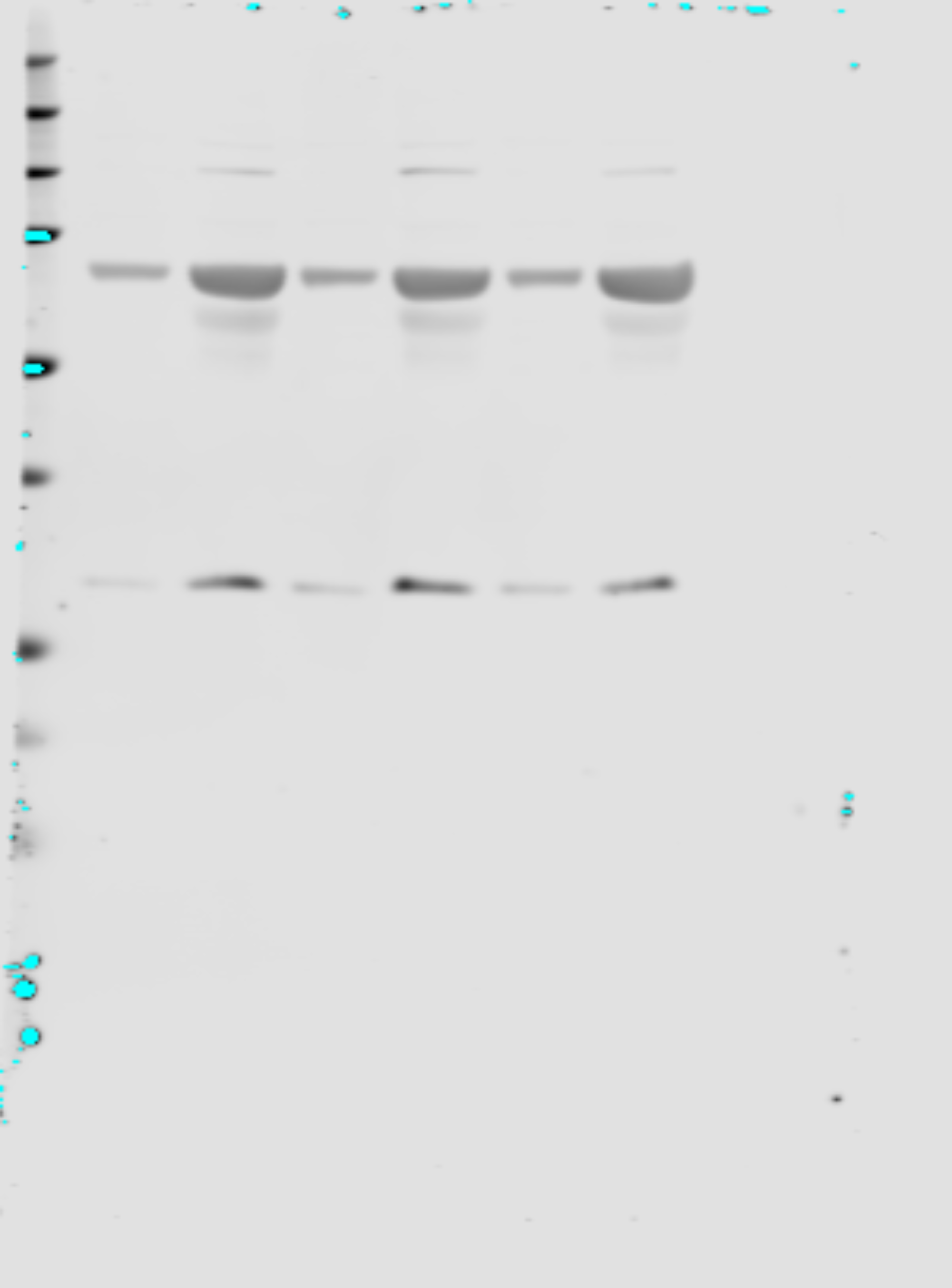

Supplement: S2 Data — The files are arranged in folders labeled according to the respective figure. (ZIP) [file ppat.1011873.s012.zip › WesternBlot Compilation/Figure Blots/Fig 5/Fig5C_Involucrin.tif]

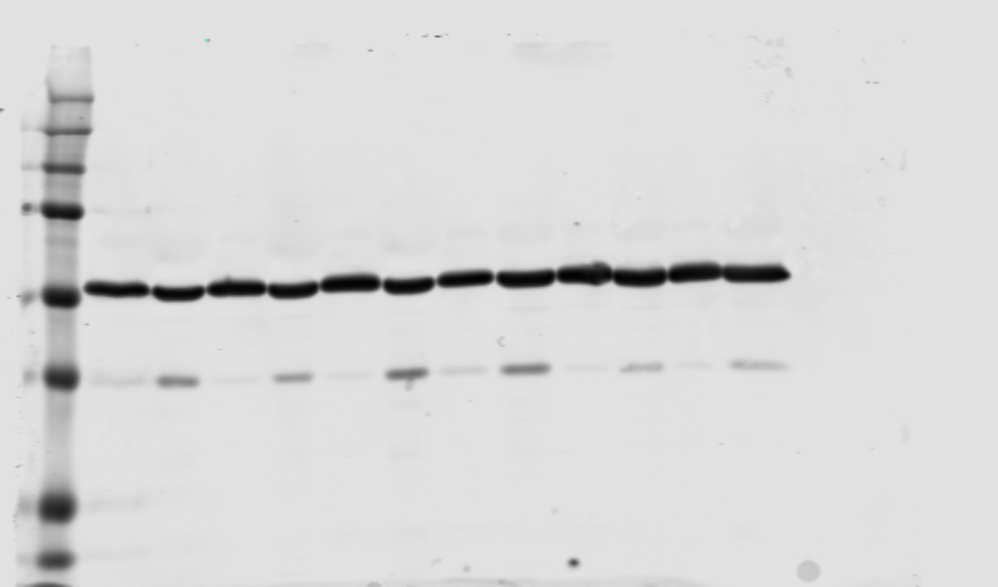

Supplement: S2 Data — The files are arranged in folders labeled according to the respective figure. (ZIP) [file ppat.1011873.s012.zip › WesternBlot Compilation/Figure Blots/Fig 5/_Fig5C BZLF1.png]

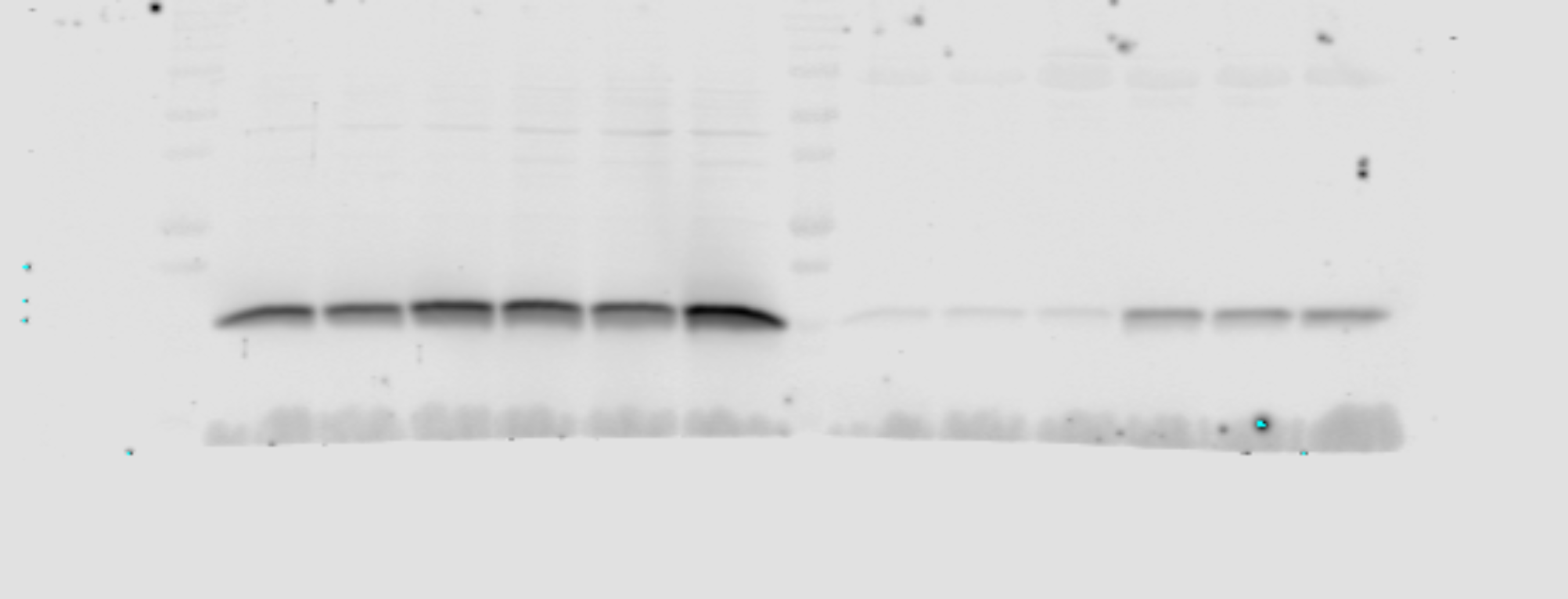

Supplement: S2 Data — The files are arranged in folders labeled according to the respective figure. (ZIP) [file ppat.1011873.s012.zip › WesternBlot Compilation/Figure Blots/FIg 7/Image_Fig7panH3ac_withreplicate.tif]

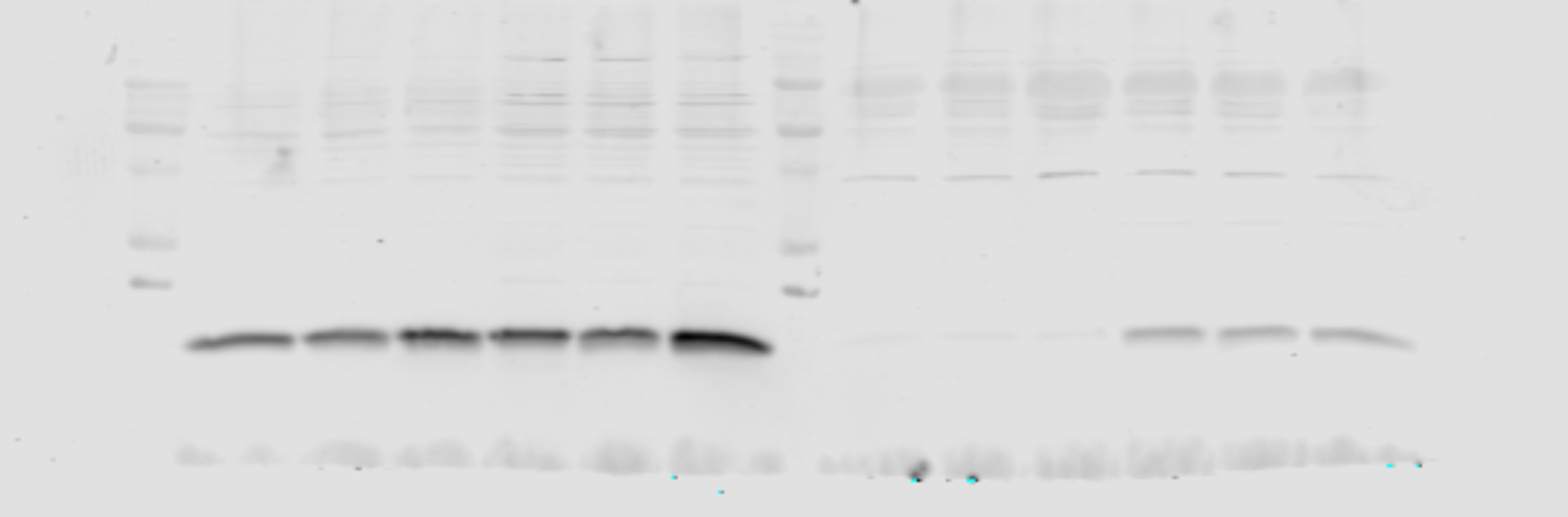

Supplement: S2 Data — The files are arranged in folders labeled according to the respective figure. (ZIP) [file ppat.1011873.s012.zip › WesternBlot Compilation/Figure Blots/FIg 7/Image_Fig7A_H3K9ac_withreplicate.tif]

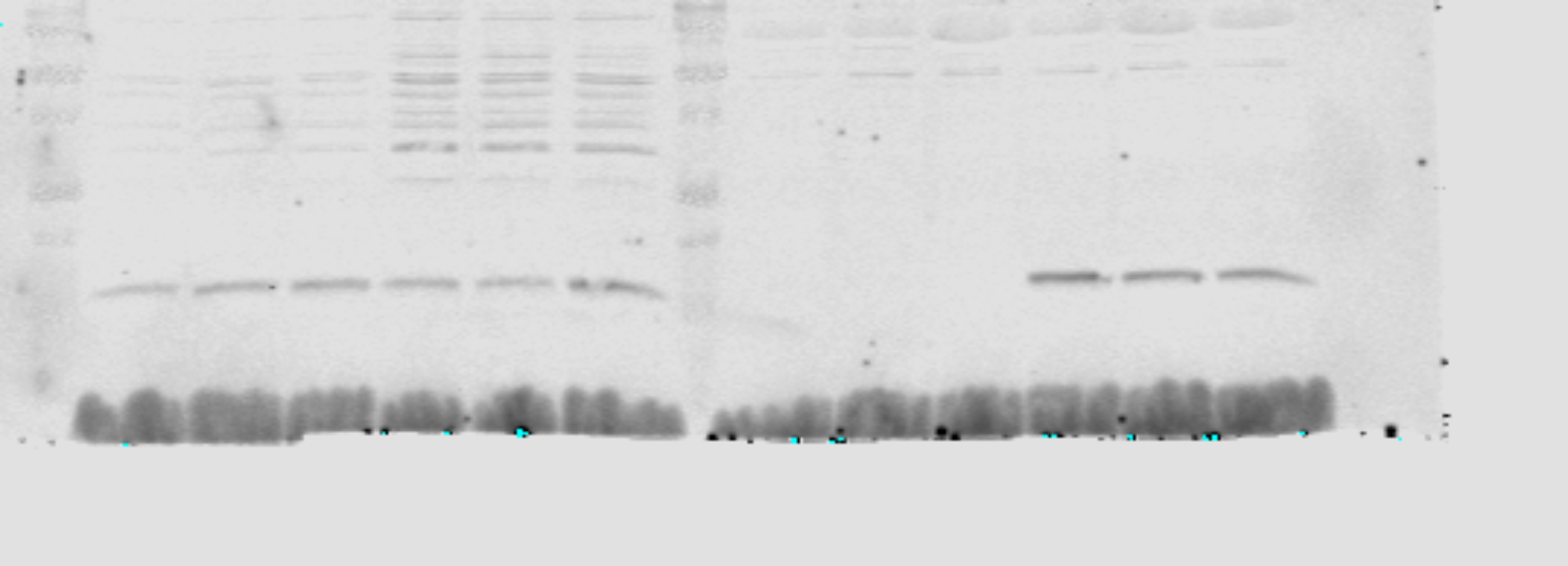

Supplement: S2 Data — The files are arranged in folders labeled according to the respective figure. (ZIP) [file ppat.1011873.s012.zip › WesternBlot Compilation/Figure Blots/FIg 7/Image_Fig7A_H3K9me2_withreplicate.tif]

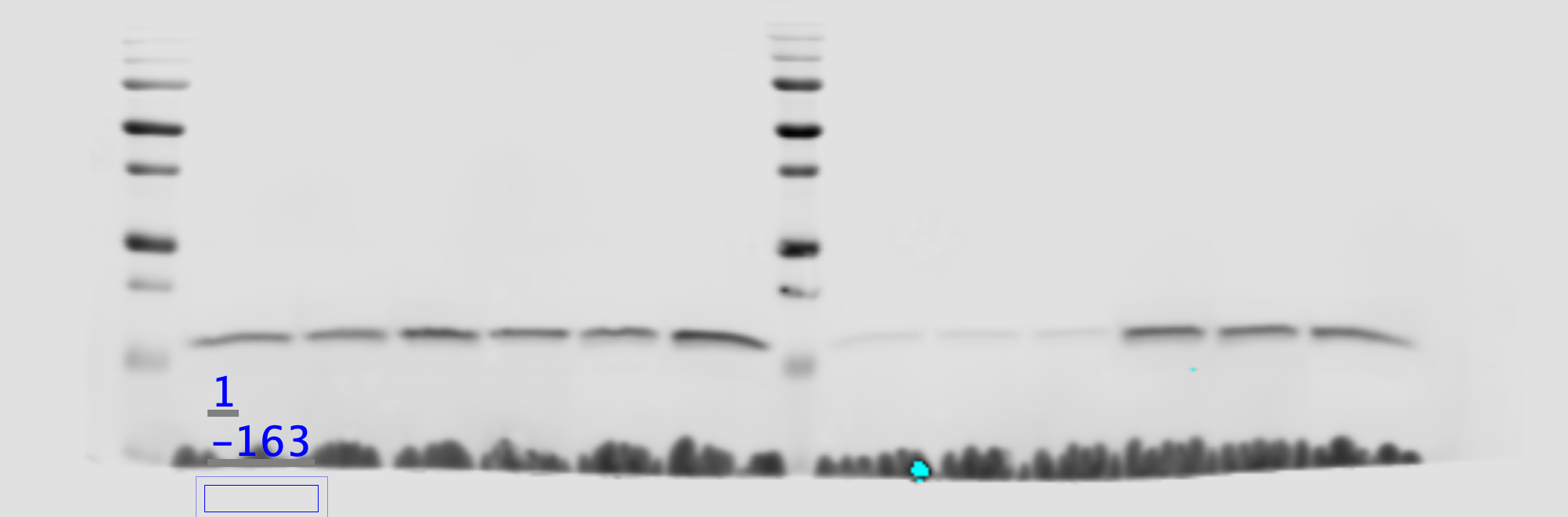

Supplement: S2 Data — The files are arranged in folders labeled according to the respective figure. (ZIP) [file ppat.1011873.s012.zip › WesternBlot Compilation/Figure Blots/FIg 7/Image_Fig7A_H3pan_withreplicate.tif]

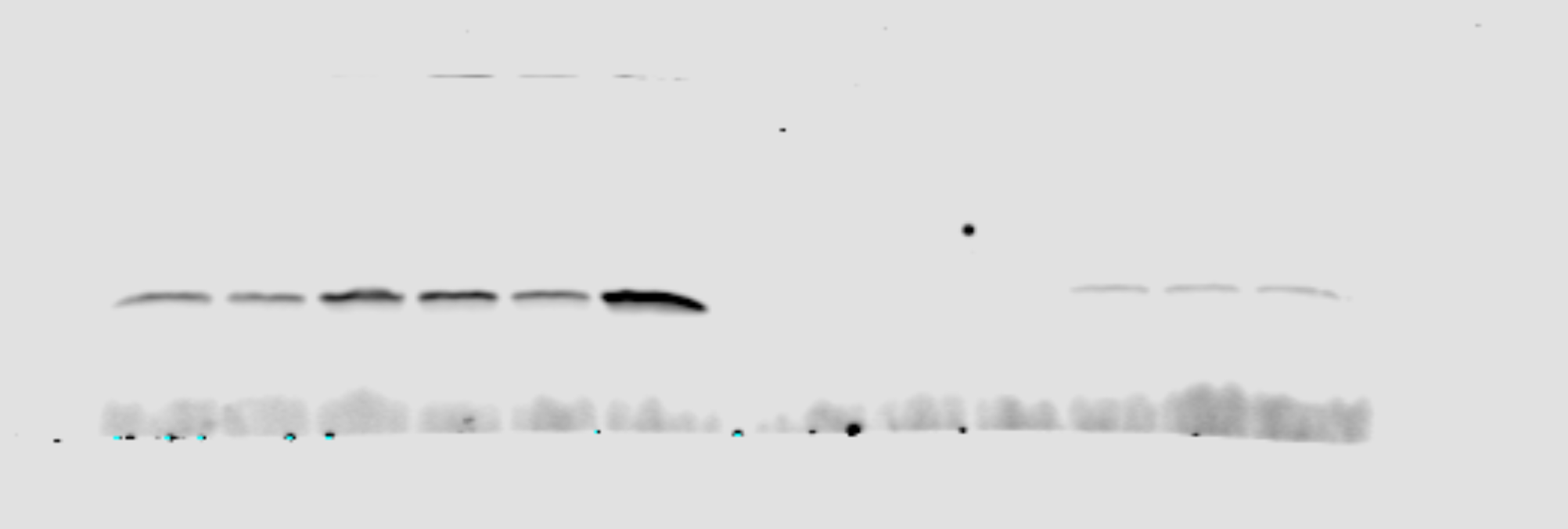

Supplement: S2 Data — The files are arranged in folders labeled according to the respective figure. (ZIP) [file ppat.1011873.s012.zip › WesternBlot Compilation/Figure Blots/FIg 7/Image_Fig7A_H3K27ac_withreplicate.tif]

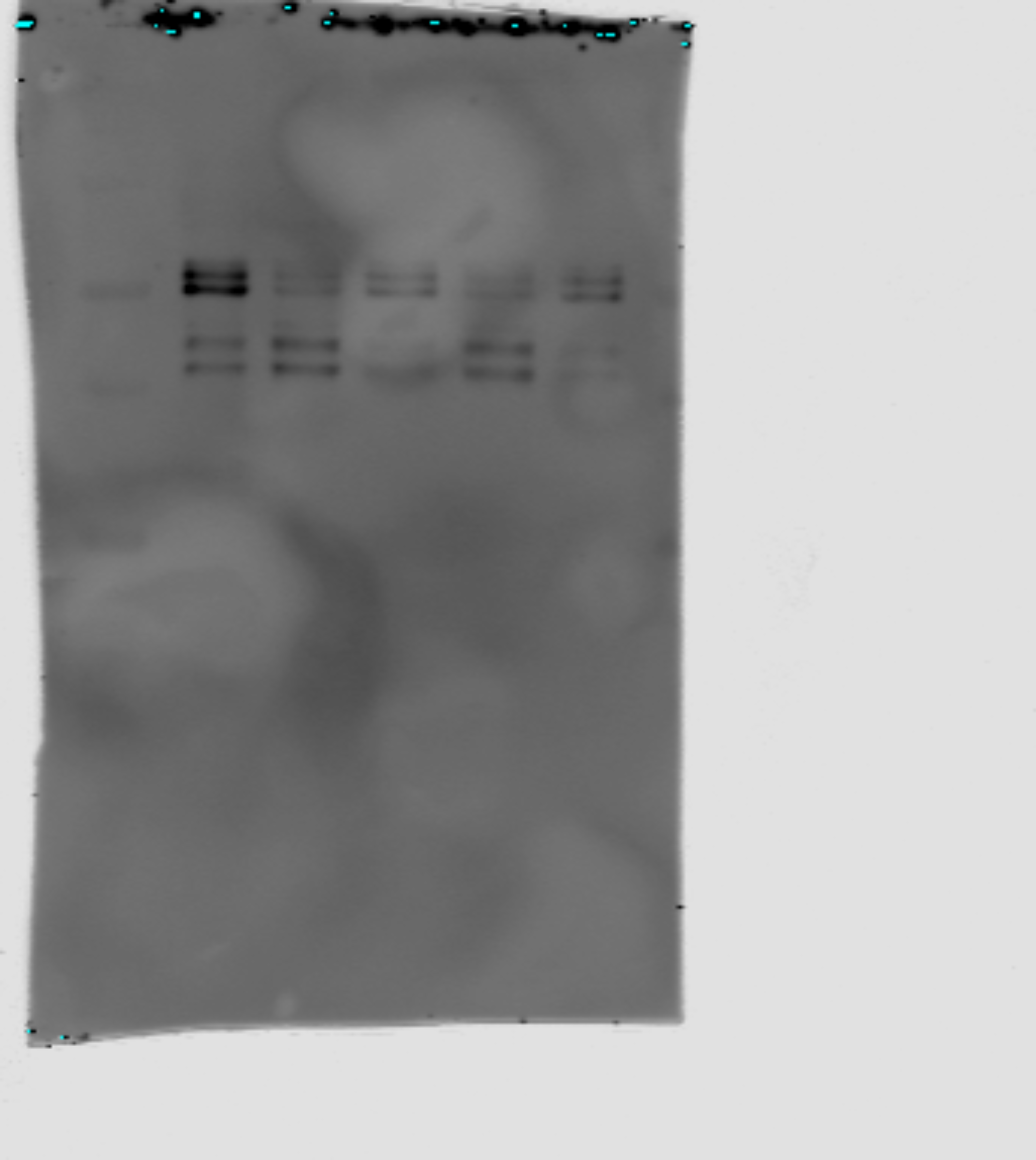

Supplement: S2 Data — The files are arranged in folders labeled according to the respective figure. (ZIP) [file ppat.1011873.s012.zip › WesternBlot Compilation/Figure Blots/Fig 6/Fig6A_CUTRUN_LEF1.tif]

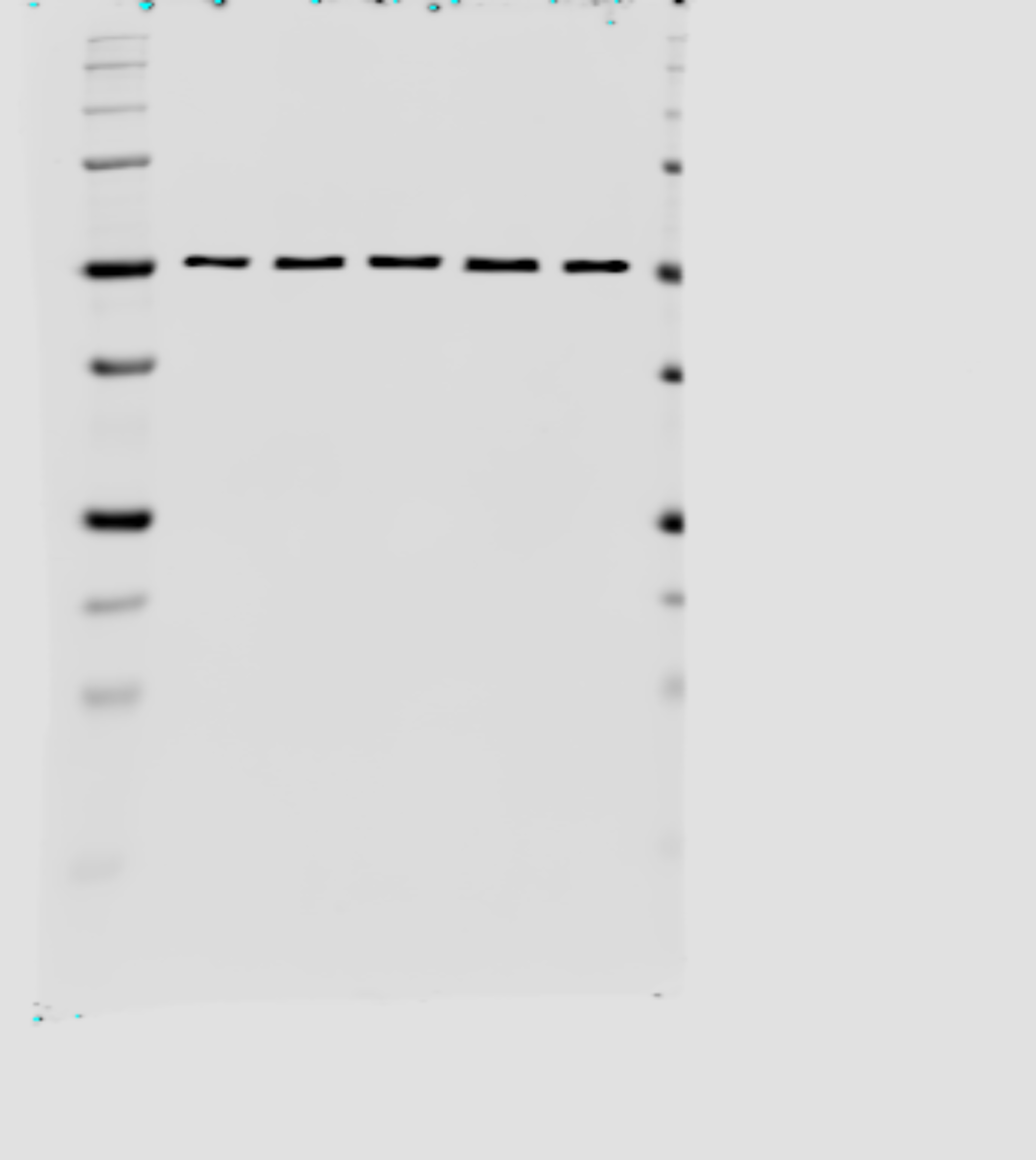

Supplement: S2 Data — The files are arranged in folders labeled according to the respective figure. (ZIP) [file ppat.1011873.s012.zip › WesternBlot Compilation/Figure Blots/Fig 6/Fig6A_CUTRUN_tubulin.tif]

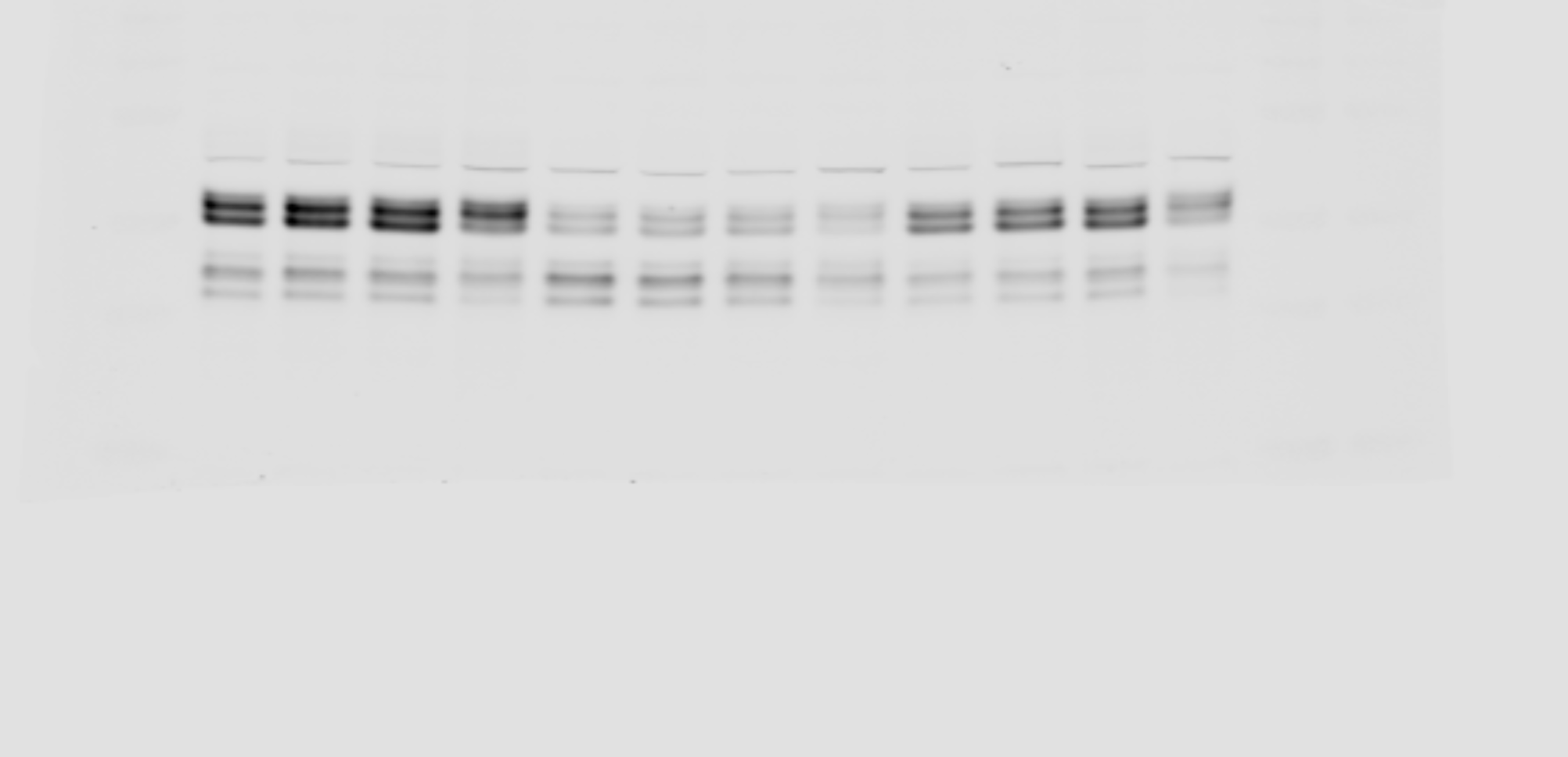

Supplement: S2 Data — The files are arranged in folders labeled according to the respective figure. (ZIP) [file ppat.1011873.s012.zip › WesternBlot Compilation/Figure Blots/Fig 8/Image_Fig8 LEF1.tif]

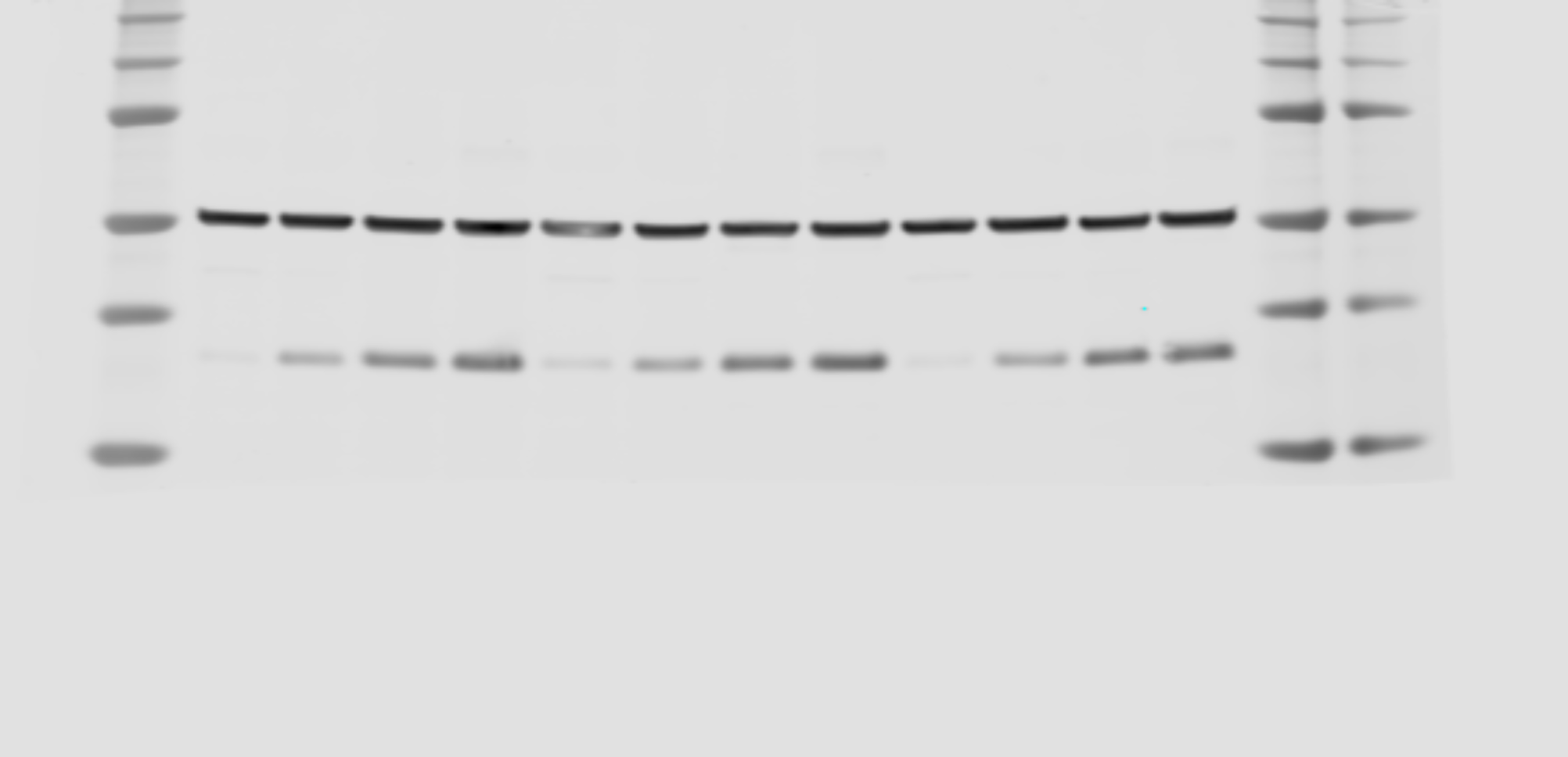

Supplement: S2 Data — The files are arranged in folders labeled according to the respective figure. (ZIP) [file ppat.1011873.s012.zip › WesternBlot Compilation/Figure Blots/Fig 8/Image_Fig8 Z_tubulin.tif]

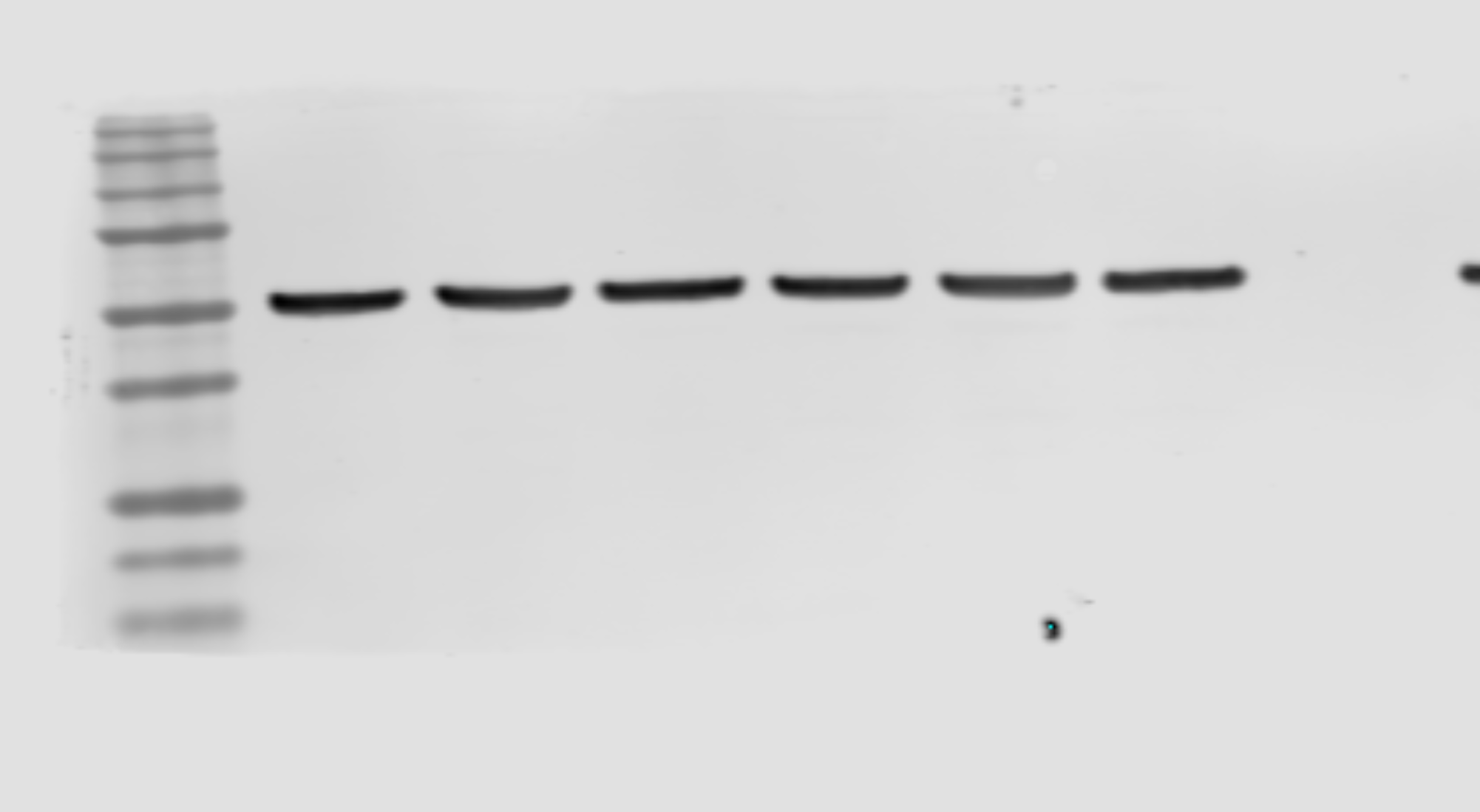

Supplement: S2 Data — The files are arranged in folders labeled according to the respective figure. (ZIP) [file ppat.1011873.s012.zip › WesternBlot Compilation/Quantification/Fig 3/_tubulinreplicate2_3.tif]

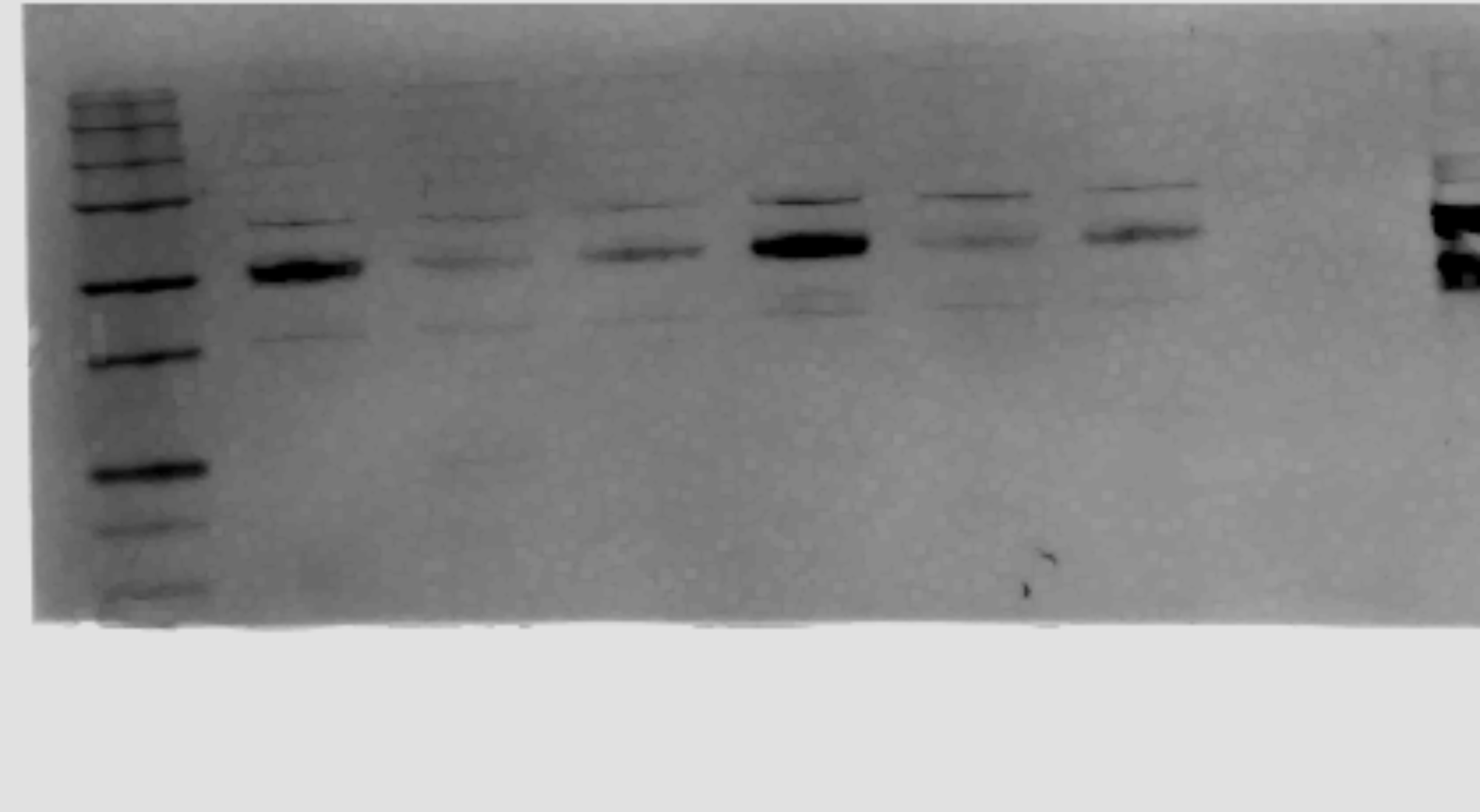

Supplement: S2 Data — The files are arranged in folders labeled according to the respective figure. (ZIP) [file ppat.1011873.s012.zip › WesternBlot Compilation/Quantification/Fig 3/_LEF1_replicate2_3.tif]

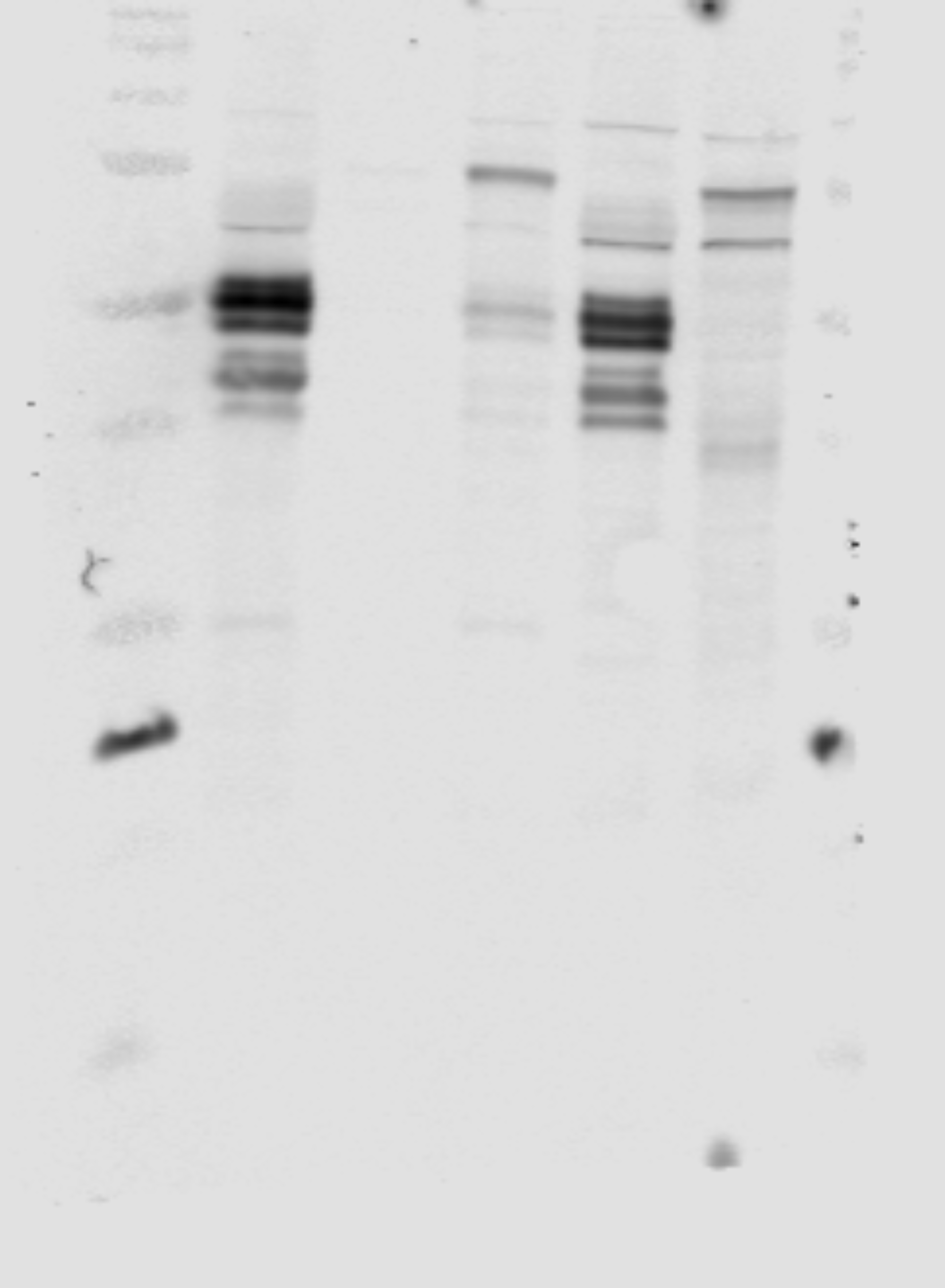

Supplement: S2 Data — The files are arranged in folders labeled according to the respective figure. (ZIP) [file ppat.1011873.s012.zip › WesternBlot Compilation/Quantification/Fig 2/Fig2_LEF1_TCF3_replicate.tif]

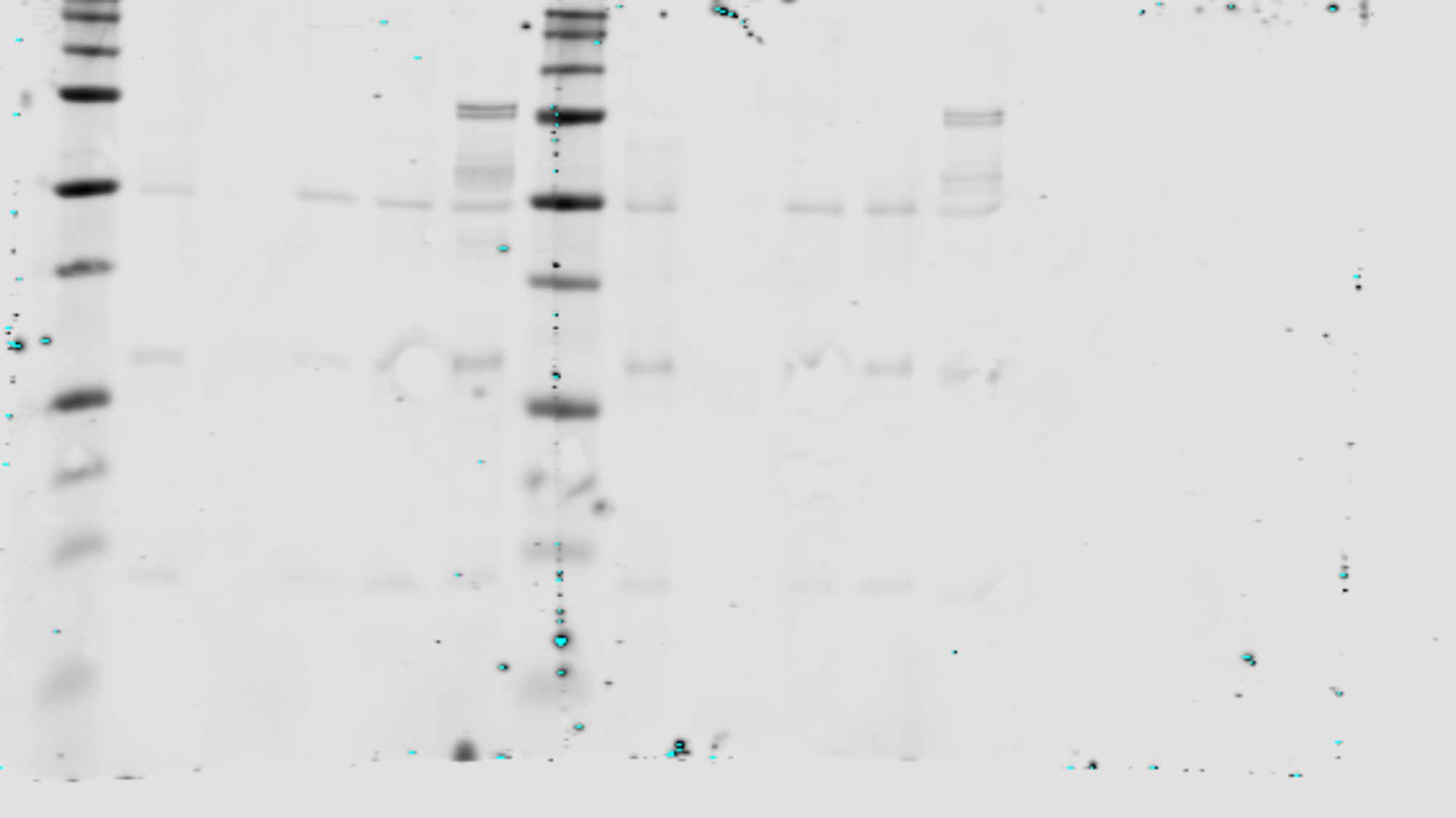

Supplement: S2 Data — The files are arranged in folders labeled according to the respective figure. (ZIP) [file ppat.1011873.s012.zip › WesternBlot Compilation/Quantification/Fig 2/Fig2_TCF4_replicate.tif]

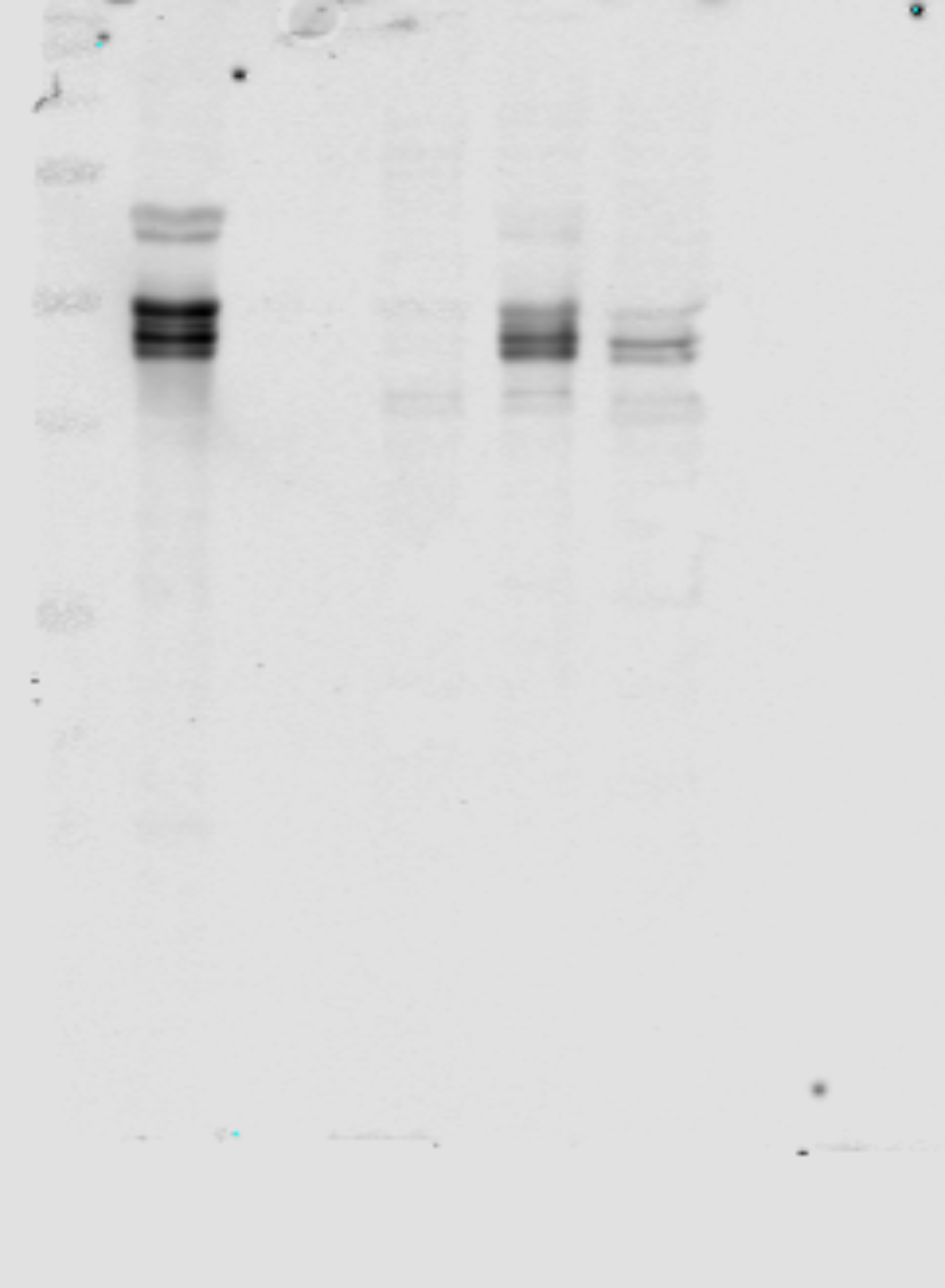

Supplement: S2 Data — The files are arranged in folders labeled according to the respective figure. (ZIP) [file ppat.1011873.s012.zip › WesternBlot Compilation/Quantification/Fig 2/Fig2_TCF1_replicate.tif]

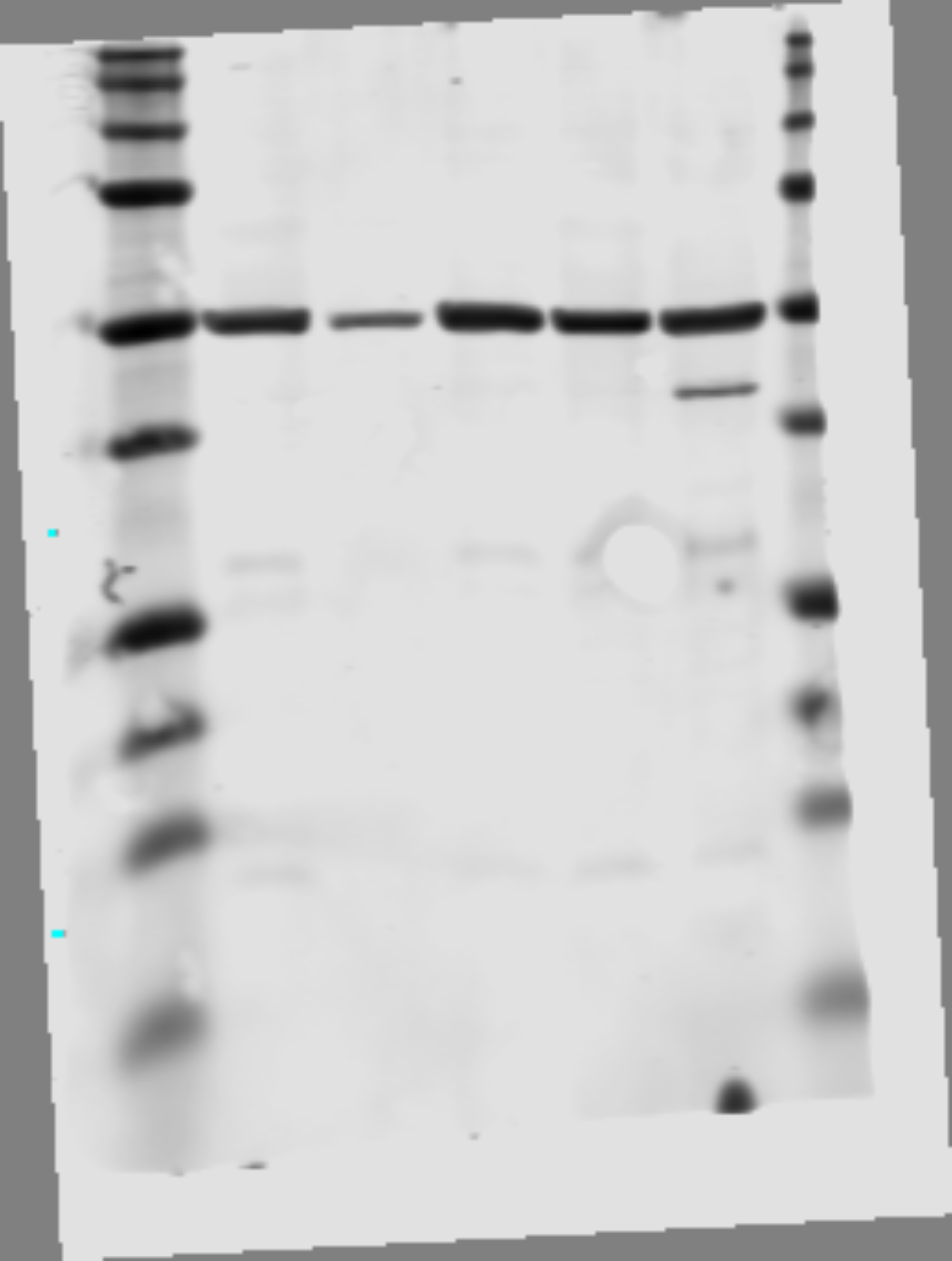

Supplement: S2 Data — The files are arranged in folders labeled according to the respective figure. (ZIP) [file ppat.1011873.s012.zip › WesternBlot Compilation/Quantification/Fig 2/Fig2_tubulin_replicate.tif]

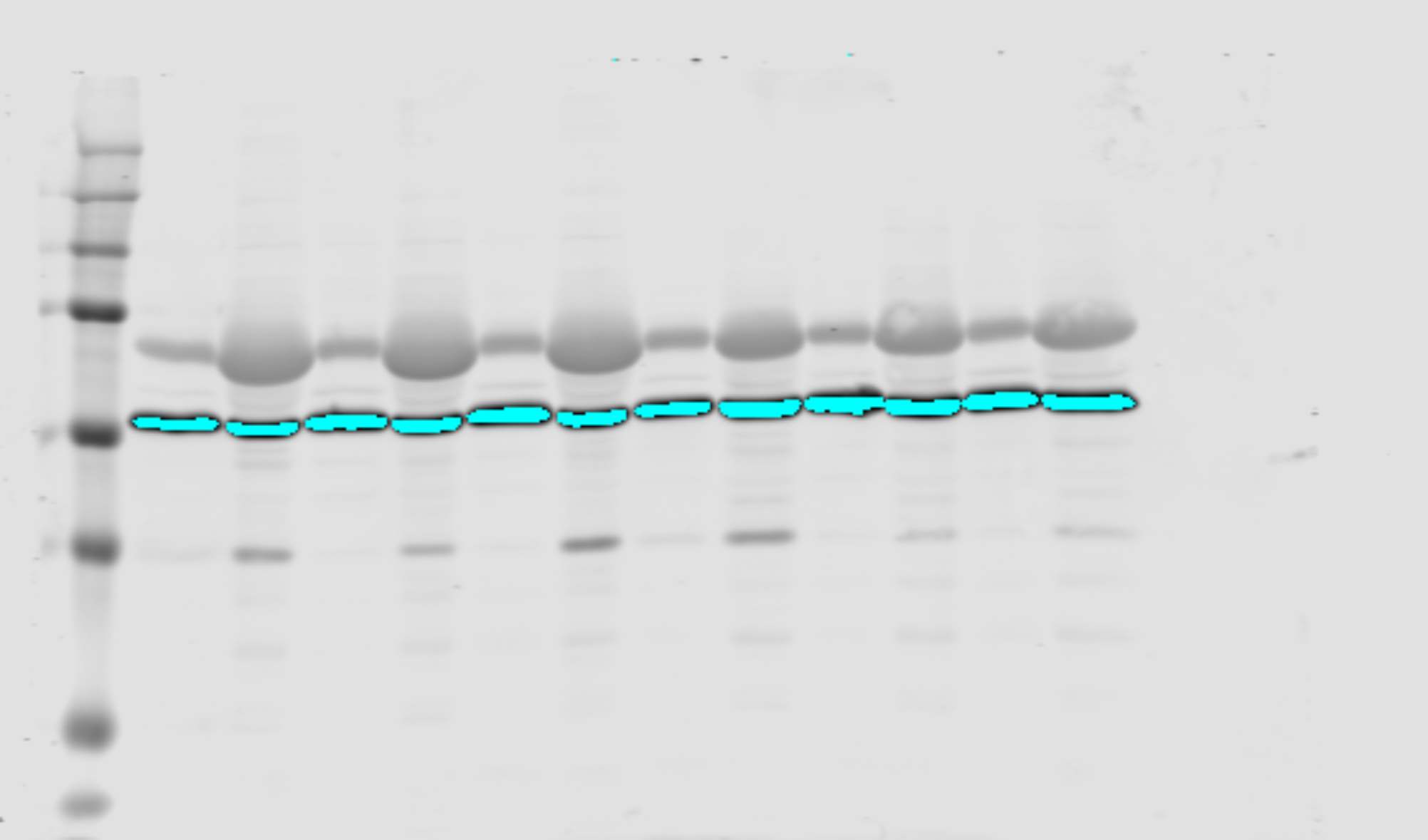

Supplement: S2 Data — The files are arranged in folders labeled according to the respective figure. (ZIP) [file ppat.1011873.s012.zip › WesternBlot Compilation/Quantification/Fig 5/INVL_replicate1.tif]

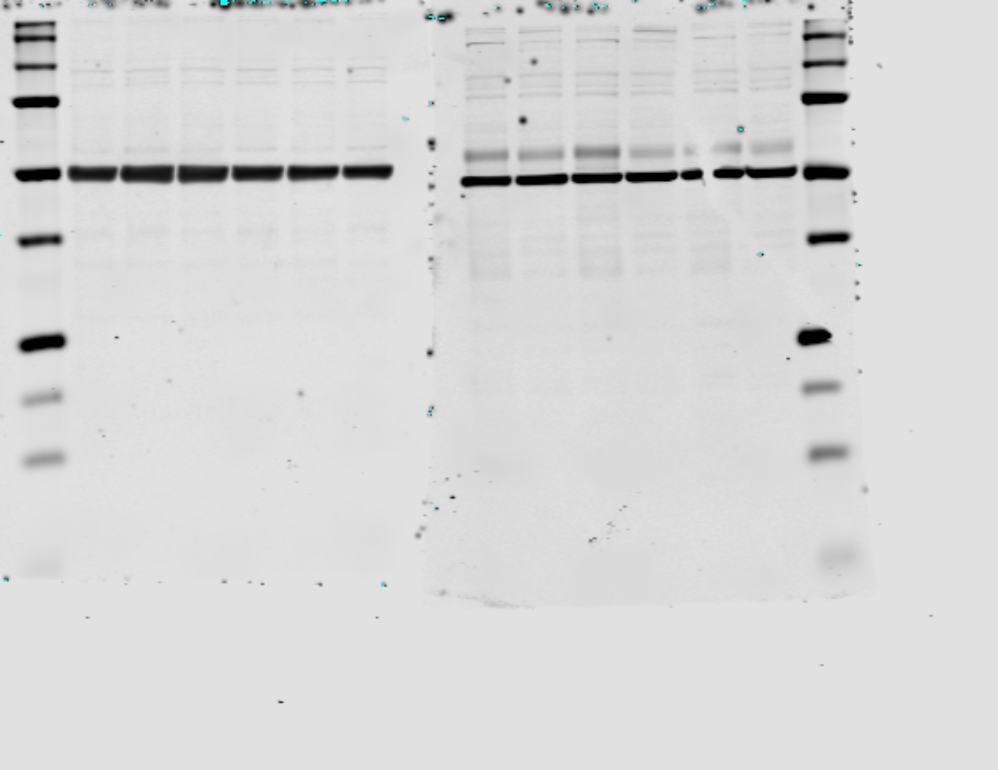

Supplement: S2 Data — The files are arranged in folders labeled according to the respective figure. (ZIP) [file ppat.1011873.s012.zip › WesternBlot Compilation/Quantification/Fig 5/Image_BetaTubulin_replicateB.png]

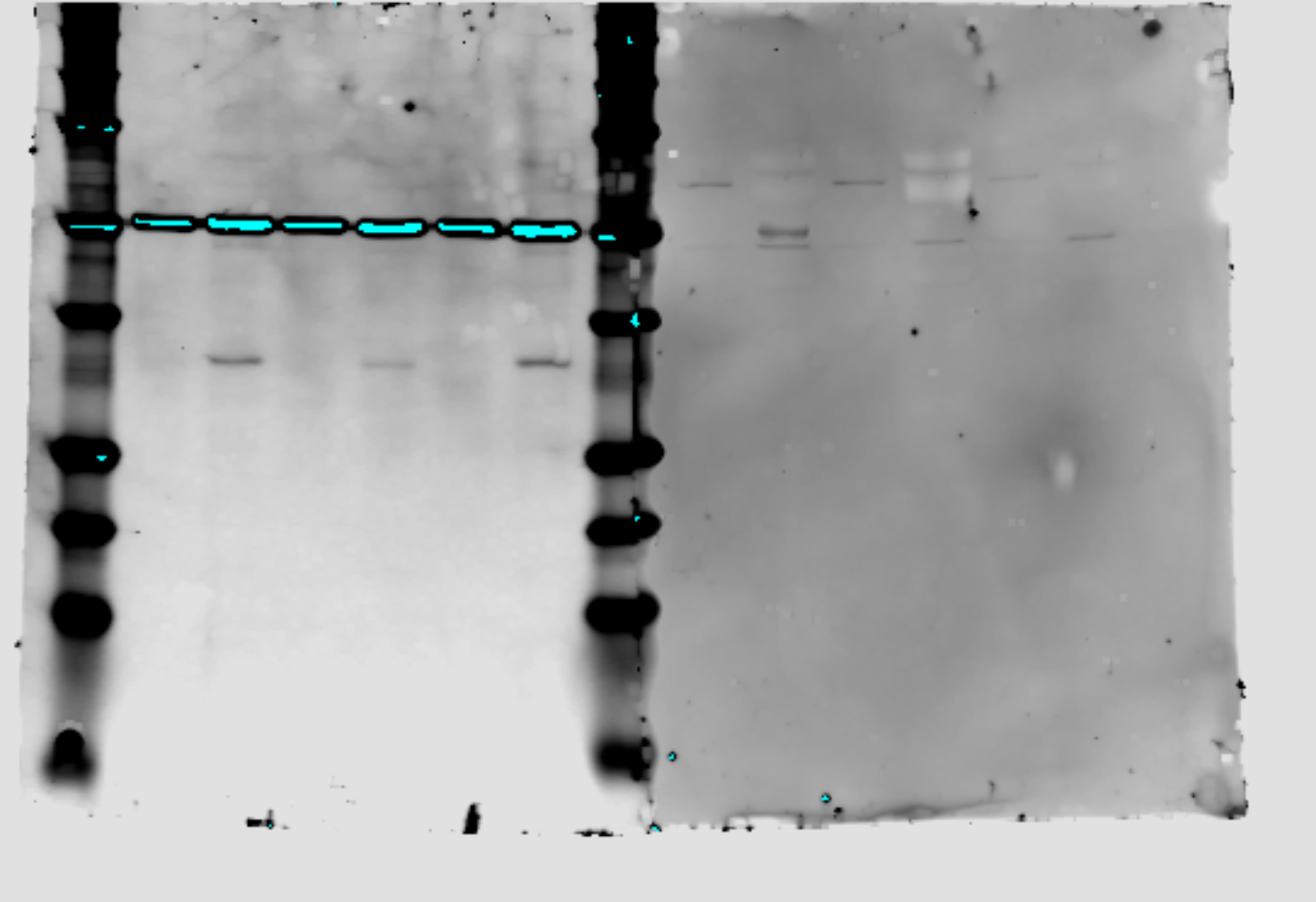

Supplement: S2 Data — The files are arranged in folders labeled according to the respective figure. (ZIP) [file ppat.1011873.s012.zip › WesternBlot Compilation/Quantification/Fig 5/_BZLF1replicate.tif]

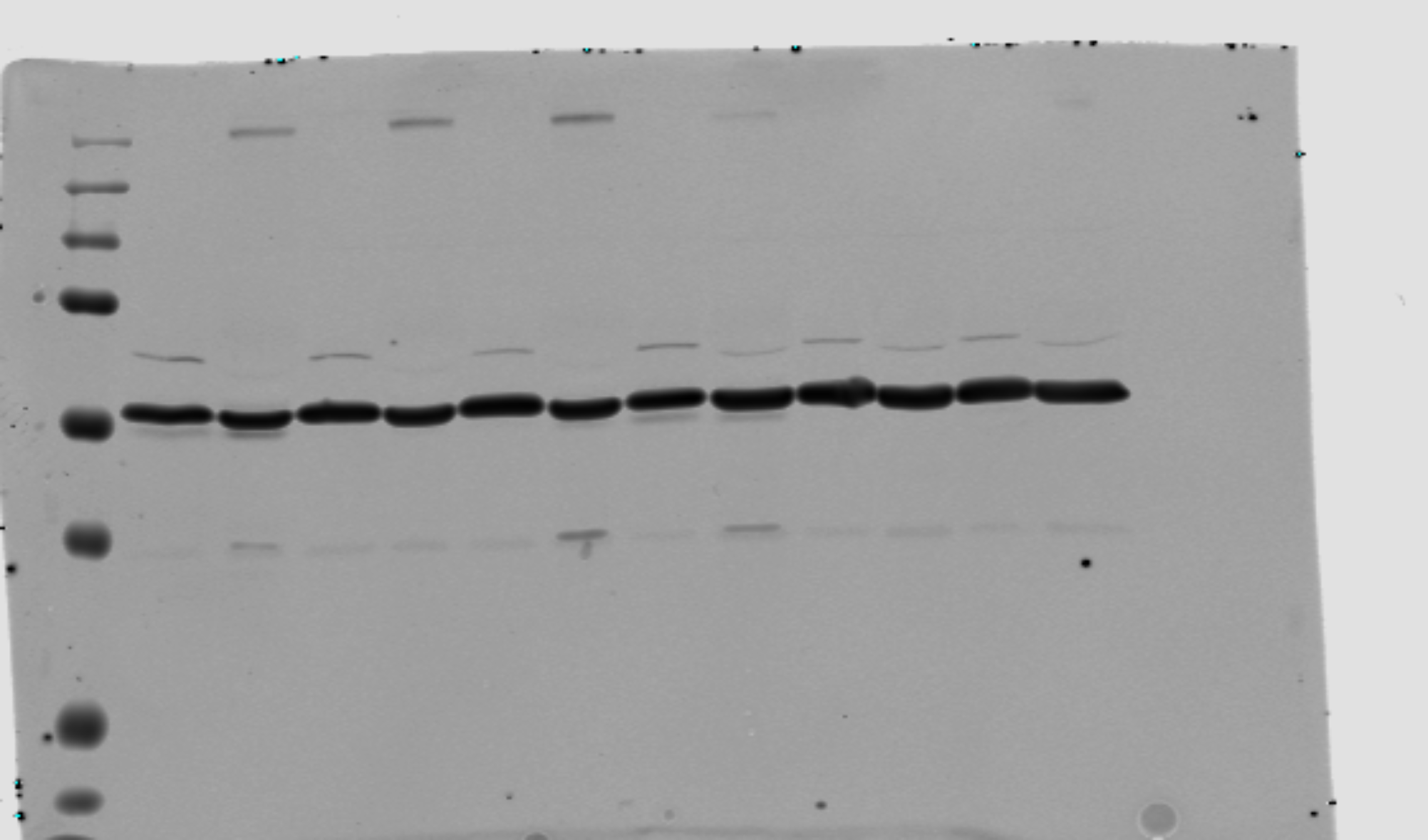

Supplement: S2 Data — The files are arranged in folders labeled according to the respective figure. (ZIP) [file ppat.1011873.s012.zip › WesternBlot Compilation/Quantification/Fig 5/Fig5C_BZLF1_LEF1_tub.tif]

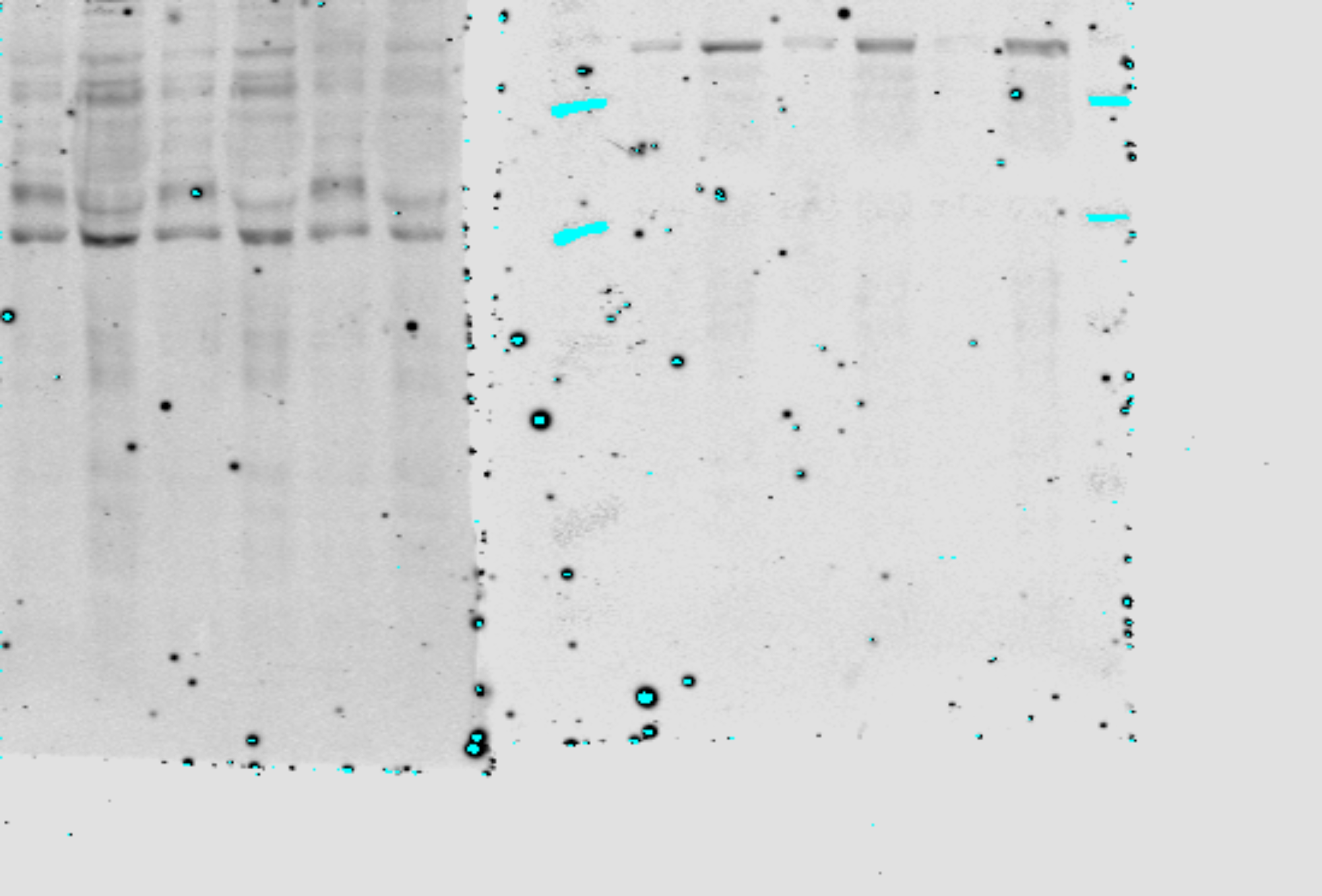

Supplement: S2 Data — The files are arranged in folders labeled according to the respective figure. (ZIP) [file ppat.1011873.s012.zip › WesternBlot Compilation/Quantification/Fig 5/_KLF4right_BLIMP1left_replicate.tif]

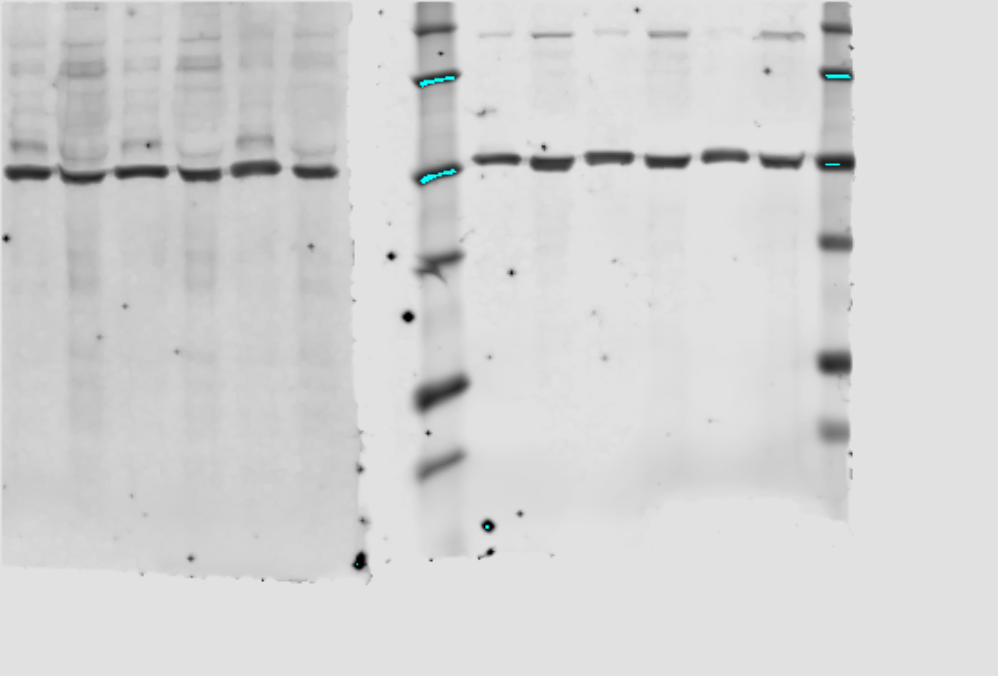

Supplement: S2 Data — The files are arranged in folders labeled according to the respective figure. (ZIP) [file ppat.1011873.s012.zip › WesternBlot Compilation/Quantification/Fig 5/tubulin_Blimp1_replicate 3.png]

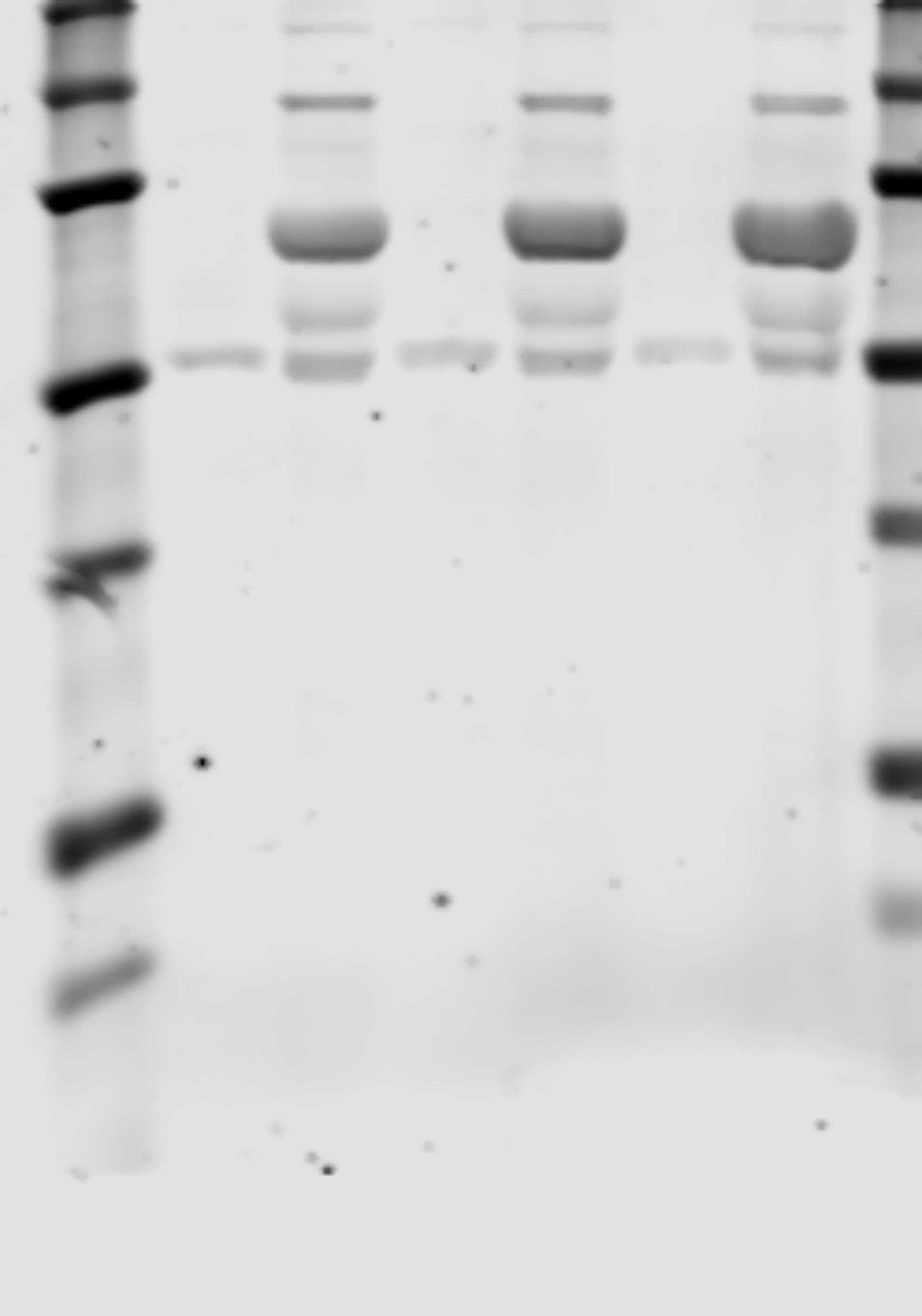

Supplement: S2 Data — The files are arranged in folders labeled according to the respective figure. (ZIP) [file ppat.1011873.s012.zip › WesternBlot Compilation/Quantification/Fig 5/_INVLreplicate.tif]

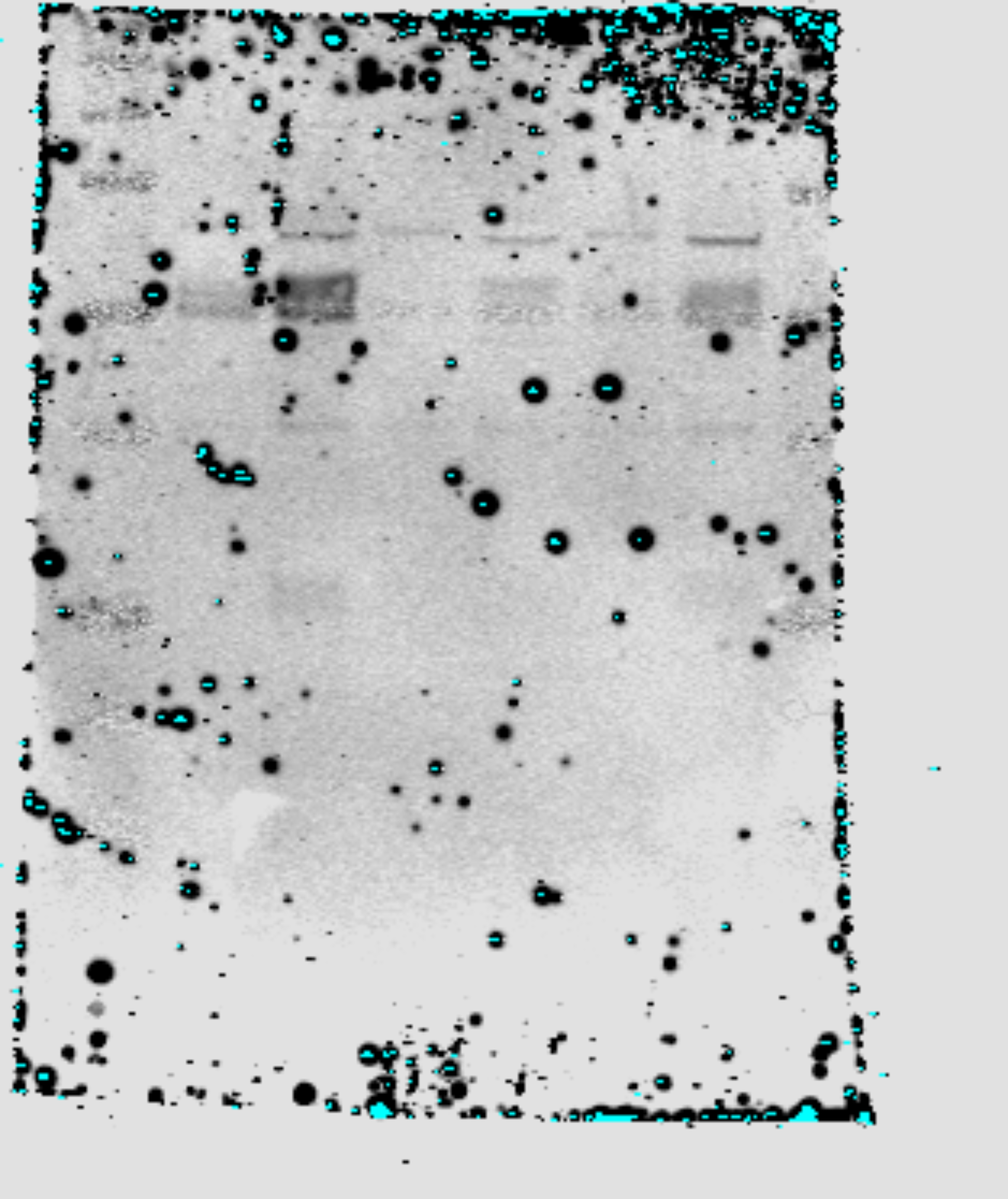

Supplement: S2 Data — The files are arranged in folders labeled according to the respective figure. (ZIP) [file ppat.1011873.s012.zip › WesternBlot Compilation/Quantification/Fig 5/_LEF1replicate.tif]

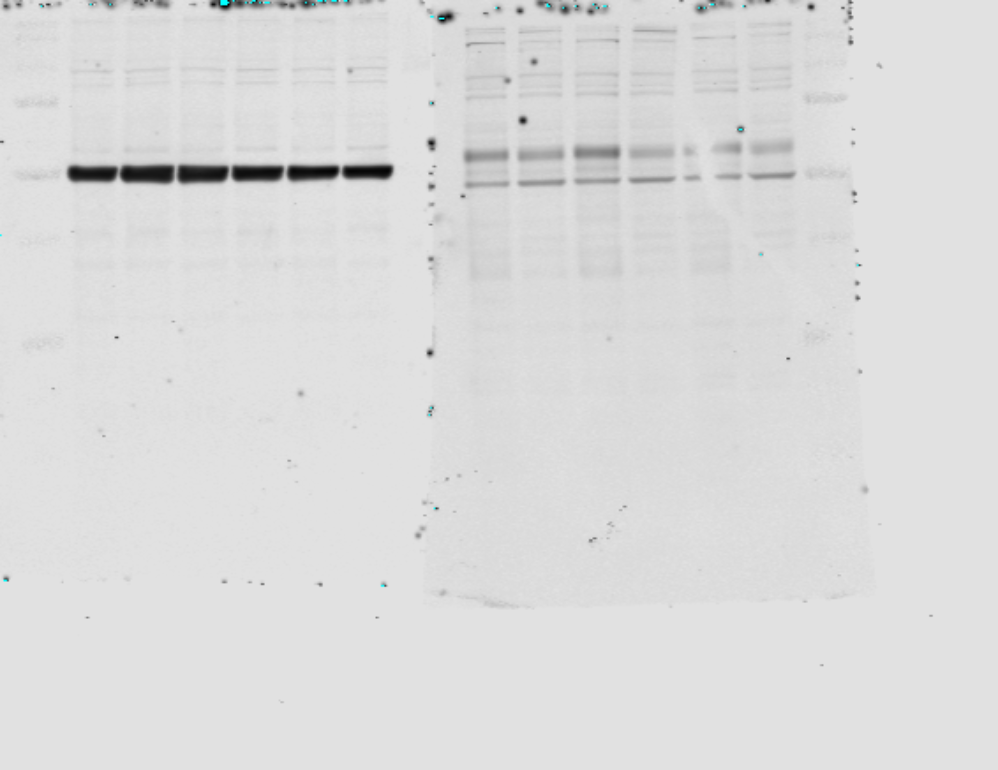

Supplement: S2 Data — The files are arranged in folders labeled according to the respective figure. (ZIP) [file ppat.1011873.s012.zip › WesternBlot Compilation/Quantification/Fig 5/Image_KLF4_replicateB.png]

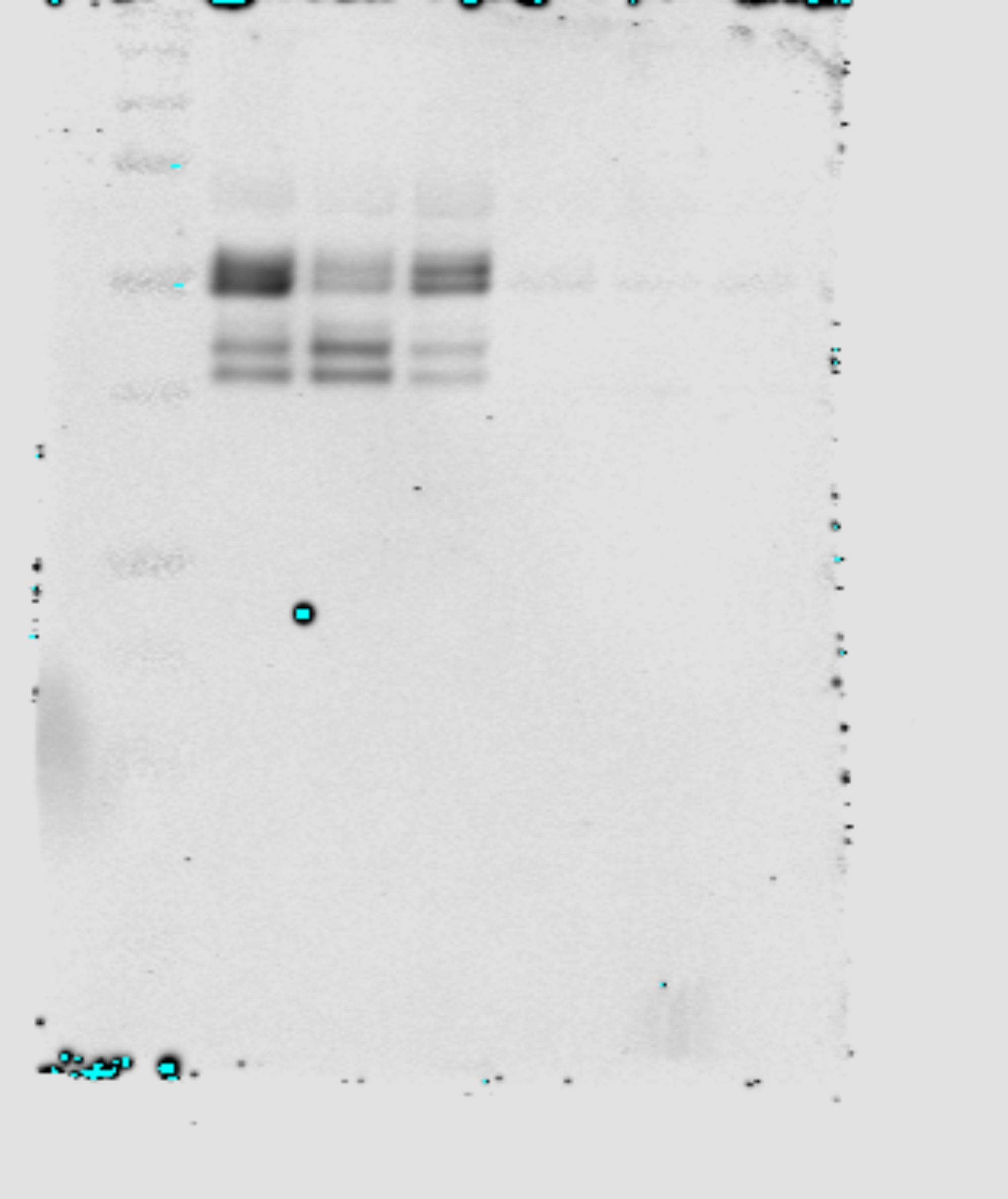

Supplement: S2 Data — The files are arranged in folders labeled according to the respective figure. (ZIP) [file ppat.1011873.s012.zip › WesternBlot Compilation/Quantification/Fig 6/_BX1_LEF1KD_LEF1replicate.tif]

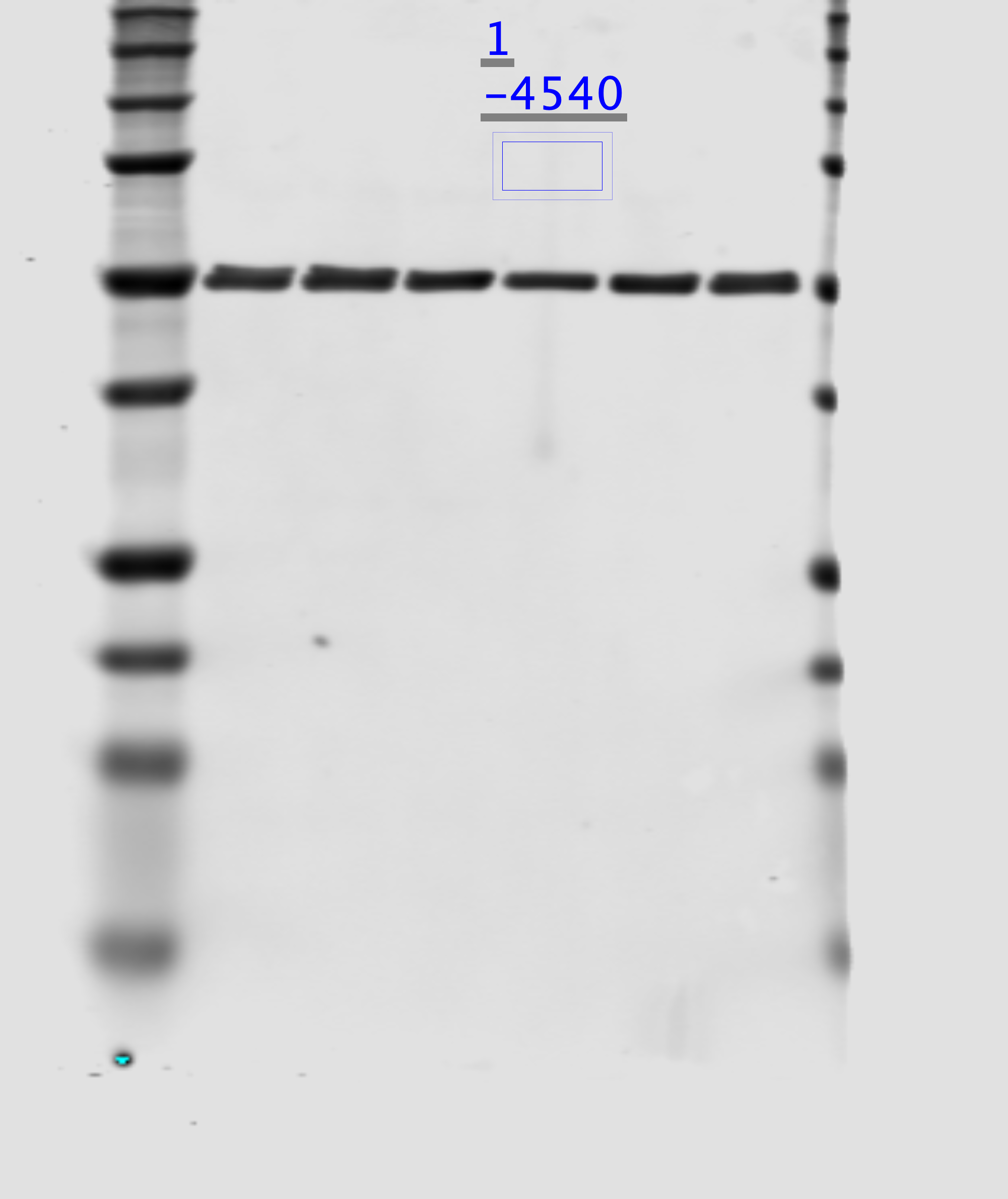

Supplement: S2 Data — The files are arranged in folders labeled according to the respective figure. (ZIP) [file ppat.1011873.s012.zip › WesternBlot Compilation/Quantification/Fig 6/_BX1_LEF1KD_tubulinreplicate.tif]

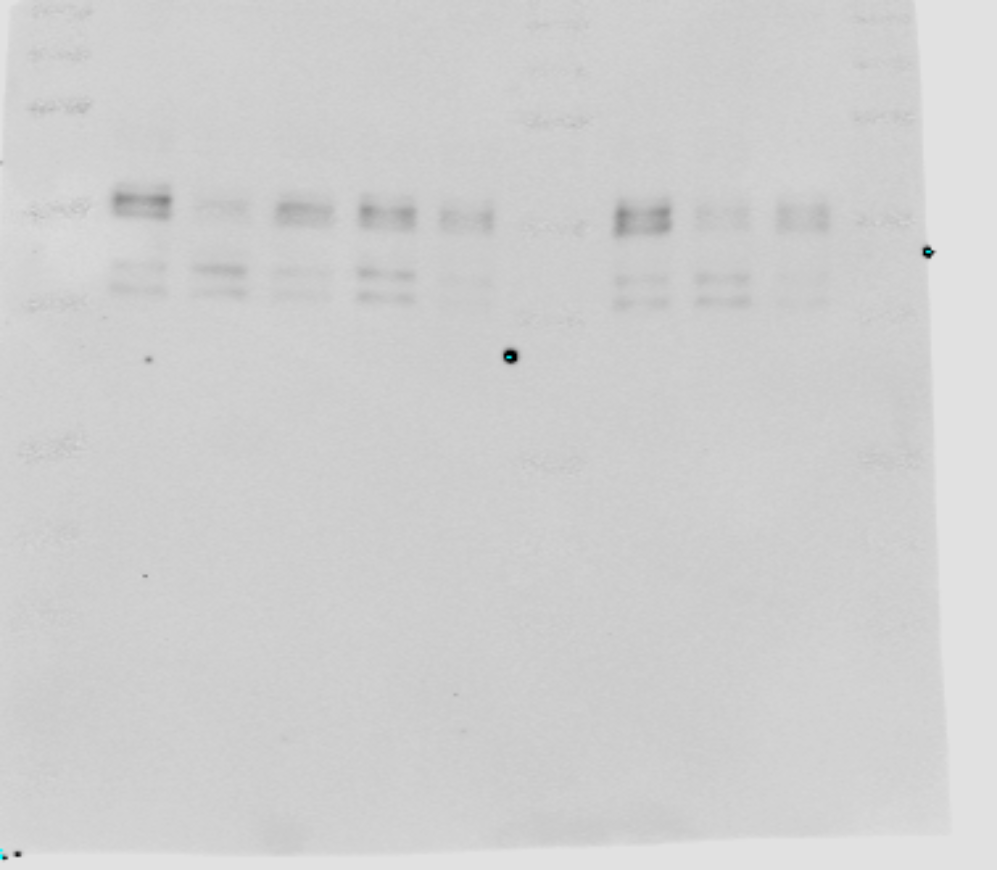

Supplement: S2 Data — The files are arranged in folders labeled according to the respective figure. (ZIP) [file ppat.1011873.s012.zip › WesternBlot Compilation/Quantification/Fig 6/Image_LEF1_replicate2.png]

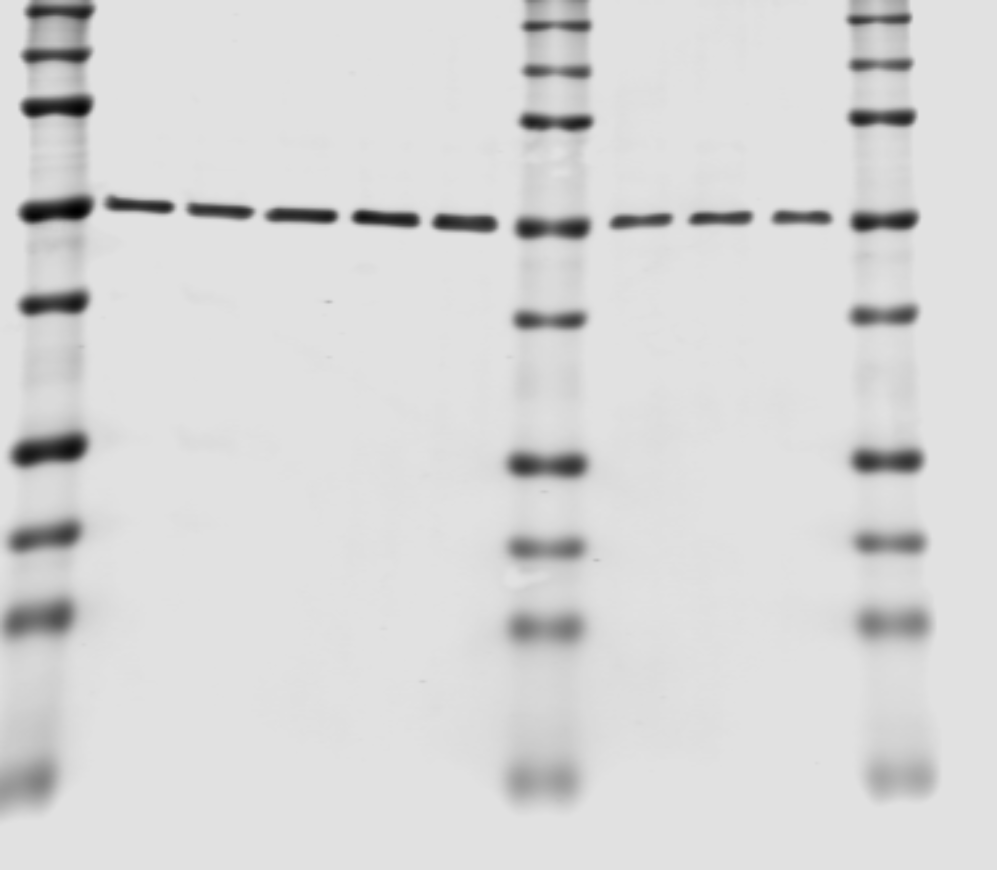

Supplement: S2 Data — The files are arranged in folders labeled according to the respective figure. (ZIP) [file ppat.1011873.s012.zip › WesternBlot Compilation/Quantification/Fig 6/Image_tubulin_replicate2.png]
