# Supplementary material for: A distinct isoform of lymphoid enhancer binding factor 1 (LEF1) epigenetically restricts EBV reactivation to maintain viral latency
Source: PLoS Pathog. 2023 Dec 19;19(12):e1011873. doi: 10.1371/journal.ppat.1011873 (PMC10763950; doi:10.1371/journal.ppat.1011873)
Supplement: S1 Table — (DOCX) [file ppat.1011873.s007.docx]

**S1 Table. Position of LEF1 peaks detected on the EBV genome**

| Peak positions | Peak motif  -/+ sense strand | | Viral Gene | Gene position | CTTTGWWSMatch | Cut&Run  detection^*^ |
| --- | --- | --- | --- | --- | --- | --- |
| 65,867 | CTTTGAAT + | | BORF2 | Exon | NO | 2/3 |
| 69,052 | CTTTGAAC + | | BMRF1/ BMRF2 | 3’ UTR/Exon | YES | 3/3 |
| 72,560 | CTTTGATC + | | BSLF1 | Exon | YES | 3/3 +qPCR |
| 82,870 | CTTTGAAC + | | EBNA 3B/3C | Exon | YES | 3/3 |
| 87,313 | CTTTGGTG - | | EBNA3B/3C | Exon | NO | 2/3 |
| 89,496 | CTTTGAAG - | | BZLF1/BRFL1 | 3’ gene | YES | 1/3 +qPCR |
| 90,624 | CTTTAAAG + | | BZLF1 | TATA box | NO | 2/3 |
| 113,905 | CATTGATG + | | BGRF1/BDRF1/BGLF2 | Intron/Exon | NO | 2/3 |
| 115,875 | CTTTGATC - | | BDLF4 | Exon | YES | 2/3 |
| 118,745 | ATTTACAG - | | BDLF2 | Exon | NO | 2/3 |
| 136,663 | CTTTGATG - | | BdRF1 | Exon | YES | 0/3 +qPCR |
| 165,900 | CTTTGATC - | | LMP-2A | Exon | YES | 2/3 |
|  | | ^*^ Represents the number of replicates out of 3 in which the CUT&RUN peak was detected | | | | |
