# Supplementary material for: A distinct isoform of lymphoid enhancer binding factor 1 (LEF1) epigenetically restricts EBV reactivation to maintain viral latency
Source: PLoS Pathog. 2023 Dec 19;19(12):e1011873. doi: 10.1371/journal.ppat.1011873 (PMC10763950; doi:10.1371/journal.ppat.1011873)
Supplement: S2 Table — (DOCX) [file ppat.1011873.s008.docx]

**S2 Table. Primary antibodies used in this study**

| **Antibody** | **Manufacturer** | **Catalog#** | **Application** |
| --- | --- | --- | --- |
| LEF1 (C12A5) | Cell Signaling Technology | 2230 | WB, IF, ChIP, CUT&RUN |
| IgG (DA1E) Isotype ctrl | Cell Signaling Technology | 66362S | ChIP, CUT&RUN |
| TCF7 (C63D9) | Cell Signaling Technology | 2203 | WB |
| TCF7L1 (D15G11) | Cell Signaling Technology | 2883 | WB |
| TCF7L2 (C48H11) | Cell Signaling Technology | 2569 | WB |
| Alpha tubulin (B-7) | Santa Cruz Biotechnology | sc-5286 | WB |
| EBV BZLF1 (BZ1) | Santa Cruz Biotechnology | sc-53904 | WB, IF, CUT&RUN |
| EBV BMRF1/Ea-D | EMD Millipore | MAB8186 | WB, IF |
| pan H3 (1B1B2) | Cell Signaling Technology | 14269S | WB |
| Acetyl-histone H3 | EMD Millipore | 06-599 | WB |
| H3K9ac | EMD Millipore | 06-942 | WB, CUT&RUN |
| H3K27ac | Abcam | ab4729 | WB, CUT&RUN |
| H3K9me2 | Upstate cell signaling solutions | 07-441 | WB |
| Involucrin (SY5) | Invitrogen | MA5-11803 | WB, IF |
| Blimp1/PRDI-BF1 (C14A4) | Cell Signaling Technology | 9115 | WB, IF |
| KLF4 | Sigma Prestige Antibodies | HPA002926 | WB, IF |
| Myc-Tag (9B11) | Cell Signaling Technology | 2276 | IF |
| WB: Western Blotting ; IF: Immunofluorescence Microscopy ; ChIP : Chromatin Immunoprecipitation ; CUT&RUN: Cleavage Under Targets and Release Using Nuclease | | | |
