# Supplementary material for: A distinct isoform of lymphoid enhancer binding factor 1 (LEF1) epigenetically restricts EBV reactivation to maintain viral latency
Source: PLoS Pathog. 2023 Dec 19;19(12):e1011873. doi: 10.1371/journal.ppat.1011873 (PMC10763950; doi:10.1371/journal.ppat.1011873)
Supplement: S3 Table — (DOCX) [file ppat.1011873.s009.docx]

**S3 Table. Primers used in this study**

| **Gene** | **Primer sequences** | **Application*** |
| --- | --- | --- |
| BZLF1 | R: 5’-AATGCCGGGCCAAGTTTAAGCAAC-3’  F: 5’-TTGGGCACATCTGCTTCAACAGGA-3’ | RT-qPCR |
| BRLF1 | R: 5’-TGGCTTGGAAGACTTTCTGAGGCT-3’  F: 5’- AATCTCCACACTCCCGGCTGTAAA-3’ | RT-qPCR |
| BMRF1 | R: 5’-CAACGAGGAAGCCGTCTT-3’  F: 5’-CAGGCTGAGGAACGAGCA- 3’ | RT-qPCR |
| BALF5 | R: 5’-GCTGACGGACGGCAAGACCC-3’’  F: 5’-GGGCAGTTCCTCGTTGCGCT-3’ | RT-qPCR |
| LEF1 total | R: 5’-ATCCCTCATCCAGCTATTGTAAC-3’  F: 5’-CTCCTGCTCCTTTCTCTGTTC-3’ | RT-qPCR |
| LEF1 variant 1 | R: 5’-CCCCCTCATCACTTACAGT-3’  F: 5’-GGAGACAAGGGATAAAAAGTAGGG-3’ | RT-qPCR |
| LEF1 variant 4 | R: 5’-CCCCCAAAAAGAAAGTGTGTG-3’  F: 5’-CTAGGTTTGTGCTTGTC-3’ | RT-qPCR |
| Cyclophillin A | R: 5’-GCAGGAACCCTTATAACCAAATCC- 3’  F: 5’-CTTGGGCCGCGTCTCC-3’ | RT-qPCR |
| BSLF1 | R: 5’-GTACAAGGGTTTGTGTCTGT-3’  F: 5’-GCCCCAATCTCTTTGATCTT-3’ | CUT&RUN-qPCR  ChIP-qPCR  RT-qPCR |
| BdRF1 | R: 5’-CTGCCTGGTGAGAAGTTG-3’  F: 5’-CGGATTTCAGCCTCATCAA-3’ | CUT&RUN-qPCR  ChIP-qPCR |
| BZLF1 | R: 5’-CGCCTCGTTTACTAATGGAATA-3’  F: 5’–ATGCCAGGAGTAGAACAATAAC-3’ | CUT&RUN-qPCR  ChIP-qPCR |
| LEF1 null | R: 5’-GACTAGCGGATGCAGCAT-3’  F: 5’-CGGTAGGGTTCGAATGG-3’ | CUT&RUN-qPCR  ChIP-qPCR |
| Simple ChIP Axin2 intron 1 | Cell signaling technology #8973 | CUT&RUN-qPCR  ChIP-qPCR |
| Simple ChIP RPL30 exon 3 | Cell signaling technology #7014 | CUT&RUN-qPCR |

*RT-qPCR=reverse transcription PCR
