# Supplementary material for: A distinct isoform of lymphoid enhancer binding factor 1 (LEF1) epigenetically restricts EBV reactivation to maintain viral latency
Source: PLoS Pathog. 2023 Dec 19;19(12):e1011873. doi: 10.1371/journal.ppat.1011873 (PMC10763950; doi:10.1371/journal.ppat.1011873)
Supplement: S4 Table — (DOCX) [file ppat.1011873.s010.docx]

**S4 Table. EBV mapped reads from CUT&RUN sequencing**

| LEF1 Occupancy on  latent EBV Genome  (Akata BX1 BL) | Total Paired-end reads | EBV mapped Reads | Data shown  in Figure |
| --- | --- | --- | --- |
| IgG Exp 1 | 4,696,160 | 17502 | 1 |
| LEF1 Exp 1 | 6,136,490 | 22788 | 1 |
| IgG Exp 2 | 6,488,084 | 54506 | 1 |
| LEF1 Exp 2 | 6,573,154 | 49302 | 1 |
| IgG Exp 3 | 4,622,236 | 31068 | 1 |
| LEF1 Exp 3 | 4,627,504 | 59414 | 1 |
|  |  |  |  |
| siNT IgG | 6,488,084 | 54506 | 6 |
| siNT LEF1 | 6,573,154 | 49302 | 6 |
| siLEF1 FL LEF1 | 7,444,950 | 82496 | 6 |
| siLEF1 FL+∆N LEF | 5,856,328 | 39926 | 6 |
|  |  |  |  |
| LEF1 mediated histone acetylation (EBV+NOK) |  |  |  |
| siNT IgG | 9,106,928 | 14642 | 7 |
| siNT H3K9ac | 3,419,604 | 1922 | 7 |
| siLEF1 FL H3K9ac | 4,136,878 | 1850 | 7 |
| siLEF1 FL+∆N H3K9ac | 5,171,898 | 4420 | 7 |
| siNT H3K27ac | 3,504,130 | 2664 | 7 |
| siLEF1 FL H3K27ac | 3,804,784 | 1866 | 7 |
| siLEF1 FL+∆N H3K27ac | 4,845,136 | 9136 | 7 |
|  |  |  |  |
| LEF1 occupancy following reactivation of EBV  (Akata BX1 BL) |  |  |  |
| Uninduced IgG | 4,241,698 | 13692 | 8 |
| Uninduced LEF1 | 4,530,120 | 18212 | 8 |
| Induced LEF1 2 hrs | 4,107,074 | 14866 | 8 |
| Induced LEF1 4 hrs | 5,081,678 | 61072 | 8 |
|  |  |  |  |

AK=AkataBX BL; NOK=normal oral keratinocytes
